# Thrombus on Mitral Annular Calcification: A Systematic Review of Management and Outcomes

**DOI:** 10.1016/j.cjco.2024.09.001

**Published:** 2024-09-17

**Authors:** Amber Cintosun, David Belzile, Maala Sooriyakanthan, Ani Orchanian-Cheff, Wendy Tsang

**Affiliations:** a Department of Medicine, University of Toronto, Toronto, Ontario, Canada; b Division of Cardiology, Toronto General Hospital, University of Toronto, Toronto, Ontario, Canada; c Library and Information Services, University Health Network, Toronto, Ontario, Canada

## Abstract

**Background:**

Mitral annular calcification (MAC) is a common chronic degenerative process of the mitral valve. Thrombus formation on MAC is a rare complication that likely contributes to the increased risk of thromboembolic events. Outcomes and management strategies for this condition are unknown. The aim of this study was to perform a systematic review to describe the management and outcomes of patients who have thrombus on MAC.

**Methods:**

The MEDLINE, Embase, and Cochrane databases were searched. Patients with a prior mitral valve intervention or prosthesis were excluded. The primary outcomes were treatment, mortality, and thromboembolic events.

**Results:**

Fifteen studies, with a total of 22 cases (patients aged 69.1 ± 14.8 years; n = 18 [82%] female) were included. Most patients presented with stroke or a transient ischemic event (n = 15; 68%) or myocardial infarction (n = 4; 18%). All patients were diagnosed with either transthoracic (n = 18; 82%) or transesophageal (n = 4; 18%) echocardiography. Seventeen patients (77%) were treated with anticoagulation therapy alone, and 5 (23%) required surgery. The most common surgical indication was prevention of recurrent embolization (n = 3; 14%). No mortality was reported. Six patients (27%) had thromboembolic events after diagnosis. For those treated with anticoagulation therapy alone, 5 (23%) had persistent thrombus with or without embolization.

**Conclusions:**

In this systematic review, patients with MAC who present with a thromboembolic event require careful echocardiographic assessment of the MAC, to exclude the presence of thrombus. Although most patients can be managed with anticoagulation therapy alone, a significant number will require surgery. Persistent thrombus, despite anticoagulation therapy, and recurrent embolization are common. Larger studies are needed to elucidate what constitutes the optimal long-term care for these patients.

## Résumé

**Contexte:**

La calcification de l’anneau mitral supportant la valve mitrale est un processus dégénératif chronique courant. La formation d’un thrombus sur l’anneau mitral calcifié est une complication rare qui contribue vraisemblablement à une hausse du risque d'événements thromboemboliques. L’issue et les stratégies de prise en charge de cette affection sont inconnues. Cette étude visait à réaliser une revue systématique afin de décrire la prise en charge et les résultats thérapeutiques des patients présentant un thrombus sur l’anneau mitral calcifié.

**Méthodologie:**

Nous avons procédé à une recherche dans les bases de données MEDLINE, Embase et Cochrane. Les patients ayant subi une intervention valvulaire mitrale ou porteurs d’une prothèse valvulaire mitrale étaient exclus. Les principaux paramètres d’évaluation étaient le type de traitement, la mortalité et la survenue d'événements thromboemboliques.

**Résultats:**

Quinze études, regroupant au total 22 cas (patients âgés de 69,1 ± 14,8 ans; n = 18 [82 %] de femmes), ont été incluses dans l’analyse. La plupart des patients avaient subi un accident vasculaire cérébral et/ou un accident ischémique transitoire (n = 15; 68 %), ou un infarctus du myocarde (n = 4; 18 %). Dans tous les cas, le diagnostic a été posé à l’aide de l’échocardiographie transthoracique (n = 18; 82 %) ou transœsophagienne (n = 4; 18 %). Dix-sept patients (77 %) ont reçu une anticoagulothérapie en monothérapie, et 5 (23 %) ont dû subir une intervention chirurgicale. Dans la plupart des cas, le recours à la chirurgie visait à prévenir une nouvelle embolie (n = 3; 14 %). Aucun décès n’a été signalé. Après le diagnostic, un événement thromboembolique a été observé chez 6 patients (27 %). Parmi les patients ayant reçu des anticoagulants en monothérapie, on a observé la récurrence du thrombus, avec ou sans embolie, chez 5 (23 %) d’entre eux.

**Conclusions:**

Selon cette revue systématique, les patients qui présentent un événement thromboembolique et dont l’anneau mitral est calcifié doivent faire l’objet d’une évaluation échocardiographique attentive de la calcification de l’anneau mitral afin d’écarter la présence d’un thrombus. Bien que dans la plupart des cas, l’administration d’anticoagulants en monothérapie soit suffisante, un nombre important de patients devront subir une intervention chirurgicale. La persistance d’un thrombus, malgré l'anticoagulothérapie, et la récurrence d’embolisation sont fréquentes. Des études de plus vaste envergure sont requises pour déterminer les soins de longue durée optimaux pour ces patients.

Mitral annular calcification (MAC) is a common, chronic, degenerative, noninflammatory process involving the mitral valve annulus that results in its progressive calcification. MAC often is diagnosed incidentally on imaging studies.^1^, ^2^, ^3^ The estimated prevalence of MAC ranges from 5% to 42%, with a higher prevalence in older age groups and women.^1^^,^^4^, ^5^, ^6^, ^7^, ^8^, ^9^, ^10^ The presence of MAC is associated with increased rates of cardiovascular and all-cause mortality, as well as cardiac and extracardiac morbidity.^3^^,^^10^, ^11^, ^12^, ^13^, ^14^, ^15^, ^16^, ^17^, ^18^ Increasingly, MAC is being recognized as a significant risk factor for cerebrovascular accidents.^19^, ^20^, ^21^, ^22^ The presence of MAC alone has been reported to be associated with a 2- to 3-fold increase in the risk of stroke.^8^^,^^21^

**Figure 1:**
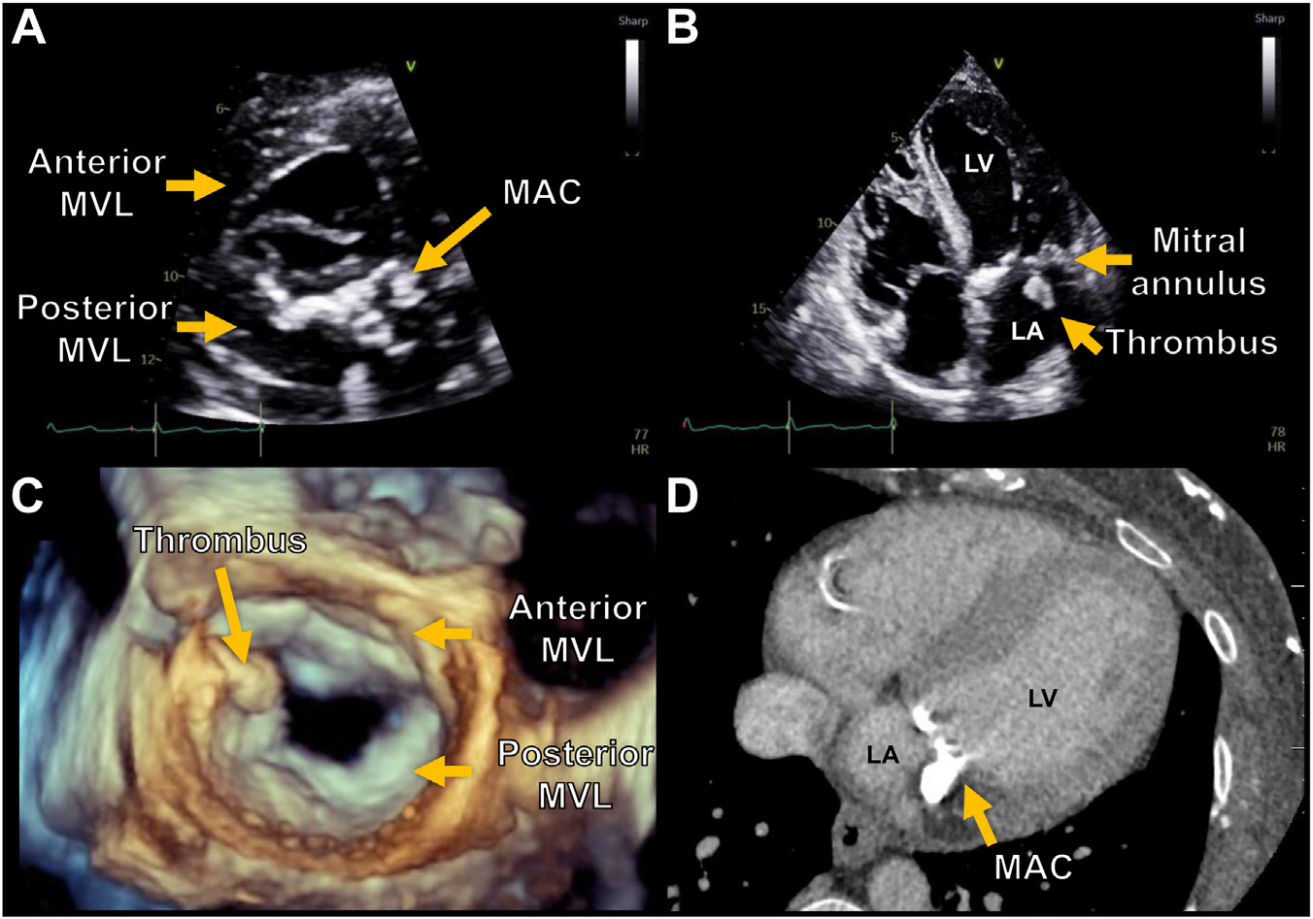
Composite image of cases with mitral annular calcification (MAC) and MAC thrombus. (**A**) Transthoracic parasternal short-axis view of the left ventricle (LV) at the level of the mitral valve, demonstrating moderate-to-severe posterior MAC); (**B**) transthoracic apical 4-chamber view demonstrating a 17 x 13 mm thrombus on the atrial side of the MAC. (**C**) 3-dimensional transesophageal echocardiogram of the mitral valve as visualized from the left atrial (LA) perspective, demonstrating the thrombus on the lateral side of the MAC. (**D**) Computed tomography demonstrating moderate-to-severe MAC and thrombus. MVL, mitral valve leaflet.

Multiple mechanisms have been proposed for the link between MAC and ischemic stroke, including MAC’s known association with increased rates of atrial fibrillation and atherosclerosis, and the embolization of calcific or caseous material on MAC.^23^, ^24^, ^25^, ^26^, ^27^, ^28^, ^29^, ^30^ Thrombus formation on MAC (Fig. 1) has been described in several case reports, and it may represent a rare mechanism for MAC’s association with stroke.^31^, ^32^, ^33^ However, the outcomes and current management strategies for MAC thrombus are poorly understood. The aim of the current study was to perform a systematic review, to describe the management and outcomes of patients with thrombus on MAC.

## Methods

This study protocol was registered with the International Prospective Register of Systematic Reviews (PROSPERO 2022 CRD42022299281)^34^ and was conducted according to the Preferred Reporting Items for Systematic Reviews and Meta-Analyses (PRISMA) guidelines.^35^

### Search strategy

A comprehensive search was performed in MEDLINE, Embase, Cochrane Database of Systematic Reviews, and Cochrane Central Register of Controlled Trials (Supplemental Appendix S1). When available, a combination of both controlled vocabulary terms, and text words (medical subject headings [MesH] and keywords) related to mitral annulus, calcification, and thrombus, were used in the subject component blocks. The search was performed for studies published from inception of the database to December 2021, and was limited to studies on humans. No language restriction was applied. No other limits were applied. Additional studies were identified by searching the article reference lists.

### Study selection

Inclusion criteria were all prospective and retrospective studies conducted in humans who had thrombus attached to MAC. MAC was defined as the accumulation of calcium along the mitral valve annulus seen on imaging studies. MAC thrombus was defined as a discrete mobile mass with characteristic imaging (computed tomography, cardiac magnetic resonance imaging, transesophageal echocardiography [TEE]) or pathology features consistent with thrombus attached to the calcified mitral annulus. Cases also were included if resolution occurred without evidence of embolization with therapeutic anticoagulation. If more than one publication was found that presented the same patient data, the most-recent study was included. Exclusion criteria included the following: study of nonhumans; patients having an artificial mitral valve, prior mitral valve intervention, rheumatic valvular disease, or calcified amorphous tumuor; and publication before 1985 due to the absence of reliable echocardiographic data for timepoints prior to this date.

### Outcomes

Our primary outcomes of interest were death, thromboembolic events (including stroke and myocardial infarction), and the need for cardiac surgery after the diagnosis of MAC with thrombus. Our secondary outcome of interest was recurrence of MAC thrombus.

### Data extraction and quality evaluation

Two investigators (A.C. and D.B.) independently screened records for inclusion using Covidence systematic review software (Veritas Health Innovation, Melbourne, Australia). Each of these researchers was blinded to the other’s decisions. Disagreements were resolved by consensus between the 2 investigators, or by arbitration by a third investigator (W.T.) if the former method was unsuccessful. Extracted data included the following: patient characteristics (age, sex, ethnicity, comorbidities, presence of hypercoagulability); presentation; potential etiology; modality of diagnosis (eg, echocardiogram, computed tomography scan); location of MAC thrombus (atrial vs ventricular); location of thrombus attachment to the valve with MAC (leaflet, annulus, etc.); thrombus size; treatments for MAC thrombus; cardiac events; cardiac intervention; thromboembolic events; in-hospital death; follow-up (if any); and timeline.

Quality assessment of the included studies was performed using a modified version of Murad et al.’s approach to methodological quality and synthesis of case series and case reports (Supplemental Table S1).^36^ Risk of bias was assessed based on the following 4 key components: (i) patient selection (ie, whether the patient selected represented the whole experience of the investigators); (ii) ascertainment (ie, exposure and/or outcomes being adequately ascertained); (iii) causality (ie, potential alternative causes were sufficiently investigated and follow-up duration was long enough for the outcome to be able to occur); and (iv) reporting (ie, sufficient details to allow practitioners to make inferences related to their own practice). The causality component was modified to exclude the dose–response effect and the challenge–rechallenge phenomenon criteria as they are not relevant to MAC thrombus management. Each item was scored as “yes” or “no” (1 point per item, for a maximum score of 6 points), and the overall methodological quality and/or risk-of-bias level was rated as low, moderate, or high.

### Data synthesis and analysis

We used the Preferred Reporting Items for Systematic Reviews and Meta-analyses (PRISMA) guidelines to report our findings.^35^ Continuous variables were summarized using medians and interquartile ranges. Categorical variables were reported as proportions.

## Results

### Study selection

**Figure 2:**
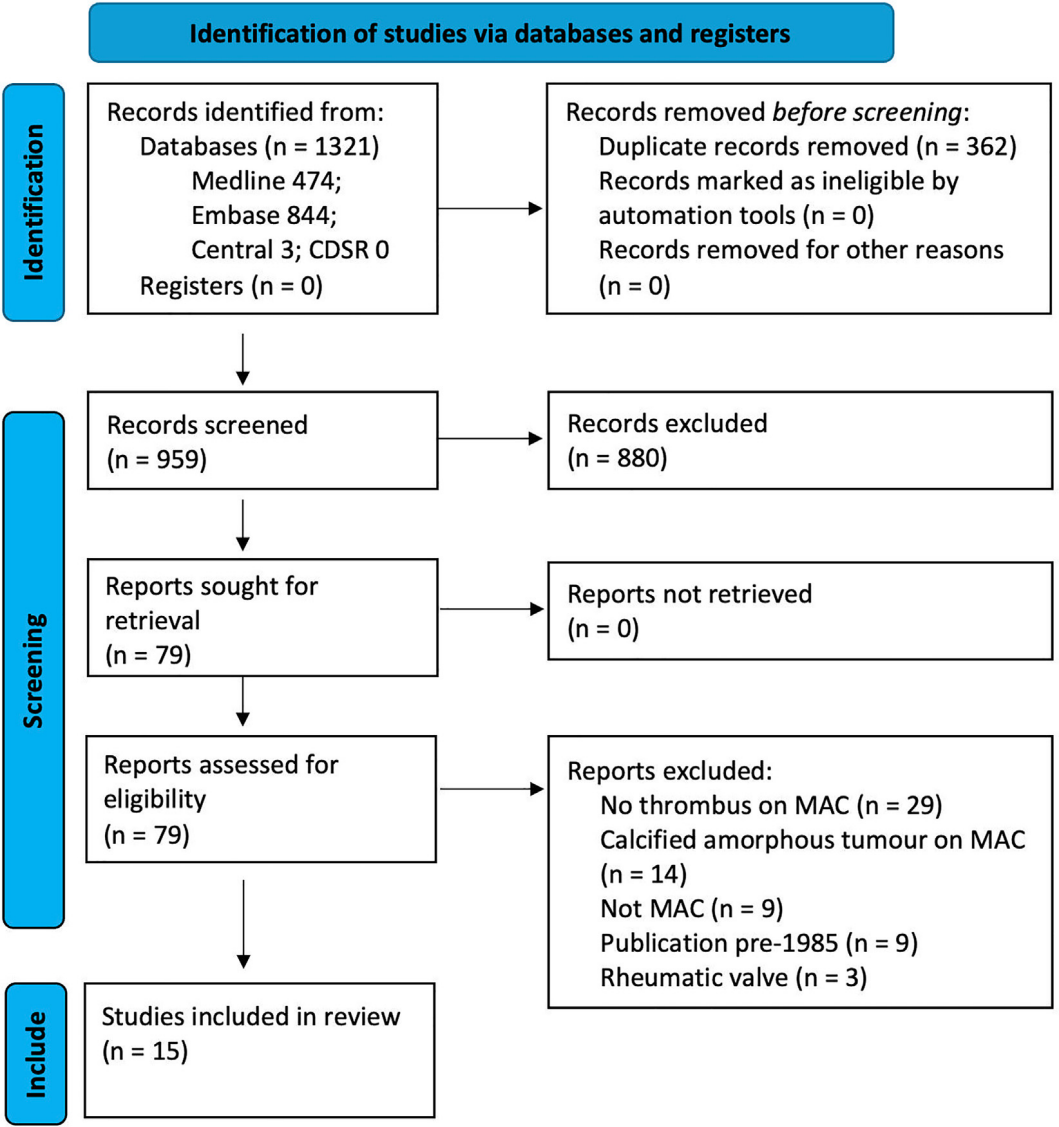
Flowchart demonstrating the process of study selection according to the Preferred Reporting Items for Systematic Reviews and Meta-Analyses (PRISMA) guidelines for systematic reviews.^35^ CDSR, Cochrane Database of Systematic Reviews; CENTRAL, Cochrane Central Register of Controlled Trials; MAC, mitral annular calcification.

The literature search approach, review process, and reasons for study exclusion are presented in Figure 2. Characteristics of the 15 included studies are described in Table 1^32^^,^^33^^,^^37^, ^38^, ^39^, ^40^, ^41^, ^42^, ^43^, ^44^, ^45^, ^46^, ^47^, ^48^, ^49^; they consist of 10 case reports, 4 case series, and 1 retrospective cohort study. Excluded studies are summarized in Supplemental Table S2.

**Table 1:** Cases (n = 22) of mitral annular calcification (MAC) thrombus

Study; country	Age, y	Sex	Initial presentation	Thrombus location and size (mm)	Management	Outcome and follow-up
Stein and Soble^33^ (1995); USA	74	F	Stroke	LA side, posterior annulus; 2	Anticoagulation (warfarin)	Thrombus resolved, no recurrence
Stein and Soble^33^ (1995); USA	47	F	Stroke	LA side, posterior leaflet; 5	Anticoagulation (warfarin)	Thrombus resolved, no recurrence
Malaterre et al.^37^ (1996); France	83	F	Stroke	LV side, unknown attachment; 18	Anticoagulation (UFH followed by warfarin)	Thrombus resolved, at follow-up free of new symptoms
Eicher et al.^38^ (1997) France	60	F	Stroke	LA and LV side, unknown attachment; 27 x 10	Anticoagulation (UFH, followed by ASA + fluindione)	Thrombus resolved, no recurrence
Eicher et al. ^38^ (1997); France	73	F	TIA	LA and LV side, unknown attachment; 13 x 8	Anticoagulation (UFH, followed by ASA + fluindione)	Thrombus resolved, no recurrence
Eicher et al.^38^ (1997); France	88	M	TIA	LA side, unknown attachment; 18 x 8	Anticoagulation (UFH, followed by dipyridamole + fluindione)	Thrombus resolved, no recurrence
Shohat-Zabarski et al.^39^ (2001); Israel	70	F	Stroke	LA side, unknown attachment; N/A	Initialanticoagulation, but eventually stopped due to bleeding (warfarin)	Initial reduction with anticoagulation, with stabilization thereafter, but recurrent emboli at 5 mo to eye after anticoagulation was stopped with persistent thrombus
Shohat-Zabarski et al.^39^ (2001); Israel	69	F	Stroke	LA side, posterior leaflet; 15	Anticoagulation (warfarin)	Thrombus resolved, no recurrence
Lahey and Horton^40^ (2002); USA	22	F	MI	LA side, unknown attachment; N/A	Anticoagulation (warfarin)	Second stroke at 4 mo despite anticoagulation and found to have persistent MAC thrombus at that time
Kawano et al.^41^ (2005); Japan	50	M	Stroke	LA side, posterior leaflet; 21 x 10	Anticoagulation (UFH, followed by mitral valvuloplasty)	Thrombus persisted with reduced size with anticoagulation, followed by surgical resection with mitral valvuloplasty; follow-up N/A
Choudry et al.^42^ (2009); United Kingdom	80	F	Presyncope	LV side, posterior leaflet; N/A	Anticoagulation (abciximab)	Coronary emboli in < 1 wk despite anticoagulation, followed by successful percutaneous retrieval of the coronary emboli; follow-up N/A
Konishi-Yakushiji et al.^43^ (2010); Japan	76	F	Stroke	LV side, unknown attachment; 7 x 7	Anticoagulation (UFH, followed by ASA + clopidogrel + warfarin)	Second stroke despite anticoagulation, followed by enlargement of the mass; initiation of antiplatelet therapy with anticoagulation led to reduction of mass size (persisted at 12 mo) without any other embolic event
Sia et al.^32^ (2010); Canada	73	F	Stroke	LV side, posterior annulus; 48 x 5	Anticoagulation (UFH)	Plan for surgery but no thrombus seen on perioperative TEE; suspected resolution with heparin; follow-up N/A
Sia et al.^32^ (2010); Canada	78	F	MI	LV side, unknown attachment; 24 x 7	Anticoagulation (UFH, followed by W)	Thrombus resolved, no recurrence
Sia et al.^32^ (2010); Canada	62	M	MI	LV side, unknown attachment; 15 x 11	Anticoagulation (UFH), followed by surgical resection	Successful resection; follow-up N/A
Nagai et al.^44^ (2012); Japan	80	F	Stroke	LV side, posterior leaflet; 7 x 5	Anticoagulation (argatroban + ASA + cilostazol, followed by warfarin)	Initial reduction with anticoagulation, with persistent smaller thrombus at 8 mo, but no recurrent emboli
Mohan et al.^45^ (2016); India	73	M	Stroke	LV side, posterior leaflet; 16 x 8	Anticoagulation (UFH + ASA + clopidrogel, followed by warfarin)	Thrombus resolved, no recurrence
Hadadi et al.^46^ (2016); USA	63	F	Incidental— diabetic foot wound	LA side, posterior annulus; 9 x 6	Anticoagulation (warfarin)	Thrombus resolved, no recurrence
Singu et al.^47^ (2017); Japan	89	F	Stroke	LA side, posterior annulus; 18 x 10	Anticoagulation (warfarin)	Home warfarin for afib before and after stroke; second embolic event (stroke) 11 d after presentation, followed by complete resolution with anticoagulation; no recurrence
Suma et al.^48^ (2019); Italy	76	F	Stroke	LV side, posterior annulus; N/A	Surgical resection with MVR	Second stroke after presentation (unknown if patient on anticoagulation), followed by successful surgical resection; follow-up N/A
Chetrit et al.^49^ (2020); USA	77	F	CHF	LA side, posterior annulus; N/A	Surgical resection with MVR	Successful resection, follow-up N/A
Chetrit et al.^49^ (2020); USA	59	F	MI	LV side, AL; 16 x 5	Surgical resection	Successful resection, follow up N/A

Afib, atrial fibrillation; ASA, acetylsalicylic acid; F, female; LA, left atrium; LV, left ventricle; M, male; MI, myocardial infarction; MVR, mitral valve replacement; N/A, not available; TEE, transesophageal echocardiography; TIA, transient ischemic attack; UFH, unfractionated heparin.

### Patient population

Overall, 22 patients were identified. The median age at diagnosis was 73 years, with an interquartile range (IQR) of 62-78 years, and 82% (n = 18) were female (see Table 2). Ethnicity frequently was not described, but the locations of publication spanned 7 countries. The most common comorbidities were hypertension, type 2 diabetes, chronic kidney disease, and coronary artery disease. No history of hypercoagulability was reported in any of the patients.

**Table 2:** Patient and thrombus characteristics

Characteristics	All patients (n = 22)
Women	18 (82)
Age, y	73 ± 15.5
Hypertension	8 (36)
Dyslipidemia	1 (5)
Atrial fibrillation	1 (5)
Type 2 diabetes	6 (27)
Chronic kidney disease	3 (14)
Dialysis	2 (9)
Coronary artery disease	3 (14)
Clinical presentation	
Stroke; transient ischemic attack	15 (68)
Myocardial infarction	4 (18)
Other	3 (14)
Thrombus size, mm (largest dimension)	16 ± 90
Thrombus location (side)	
Atrial	10 (45)
Ventricular	10 (45)
Both	2 (9)

Values are n (%), or median ± interquartile range.

The most common presentation was a cerebral vascular event, which occurred in 68% of patients (n = 15), with most experiencing a stroke (n = 13), and those remaining experiencing a transient ischemic attack (n = 2). The second most common clinical presentation in 18% (n = 4) was myocardial infarction. In 14% (n = 3), thrombus on MAC was identified during investigations for other presentations, such as heart failure, presyncope, or diabetic foot wound.

### Diagnosis and imaging characteristics

All patients were diagnosed using either transthoracic echocardiography (TTE; n = 18; 82%) or TEE(n = 4; 18%). The MAC severity level was reported in 14 of 22 patients, as being severe in 11 (79%), moderate in 2 (14%), and mild in 1 (7%).

Thrombus size was reported in 17 patients (77%), and the median widest dimension was 1.6 cm, with an IQR of 0.9-1.8 cm. Thrombus was identified on the atrial side of the mitral annulus in 10 patients (46%), on the ventricular side of the mitral valve in 10 patients (46%), and on both the atrial and ventricular sides in 2 patients (8%). The circumferential location of thrombus attachment was reported in 14 patients (64%), with the most-common attachment site located posteriorly in 12 patients (55%). Only 2 cases (10%) reported anterior thrombus attachment.

### Management

**Figure 3:**
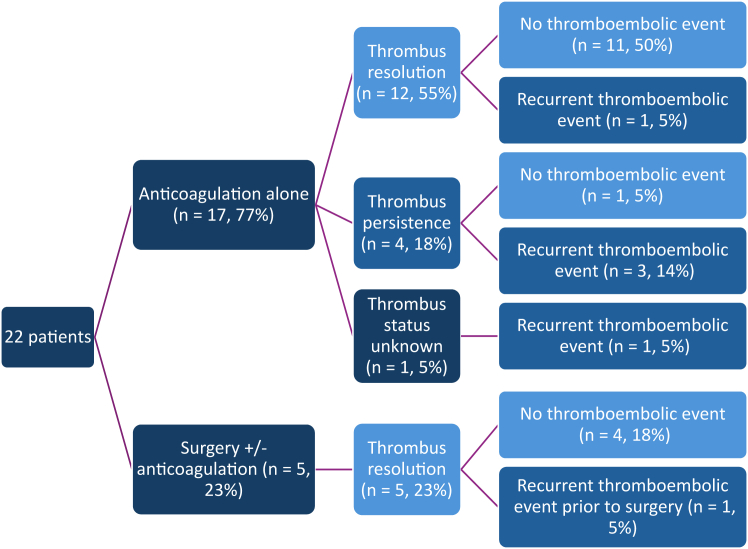
Flowchart demonstrating outcomes for patients with mitral annular calcification thrombus, managed by either anticoagulation therapy alone or surgery.

The main modalities for treatment of MAC thrombus were anticoagulation therapy and surgery. A flowchart demonstrating anticoagulation and surgical management for the patients is presented in Figure 3. Seventeen of the patients (77%) were treated only medically, with anticoagulation therapy with the potential addition of antiplatelet agents (Fig. 3). Agents used included heparin, vitamin-K antagonists (warfarin, fluindione), anti-glycoprotein IIb/IIa receptor antibody (abciximab), direct thrombin inhibitor (argatroban), dipyridamole, aspirin, and clopidogrel. Some of these agents were provided simultaneously, and some consecutively, in the same patient. Fourteen patients (64%) received a vitamin-K antagonist at some point during their therapy, and 10 (45%) received heparin. Most anticoagulated patients received warfarin alone, with a target international normalized ratio (INR) of between 2 and 3. In 6 cases, antiplatelet therapy was added to anticoagulation therapy (aspirin alone, dipyridamole alone, aspirin + cilostazol, or aspirin + clopidogrel). These combinations were sometimes used upfront (in 4 cases), and occasionally (in 2 cases), they were used due to inadequate response to anticoagulation therapy alone. The add-on strategy was effective when required. In 1 case, a higher INR target (> 3.0) was used, due to an inadequate response to an INR of between 2 and 3. The duration of anticoagulation therapy was unclear for half of the cases (n = 11), and for the remainder, treatment duration ranged from 1 week to lifelong.

Five patients (23%) underwent cardiac surgery (Table 3). Two of these 5 patients had received successful anticoagulation therapy. Whether anticoagulation therapy was received prior to surgery was unknown for the remaining 3 patients. The most common indication for surgery was prevention of recurrent embolization, which occurred in 3 patients (14%). The exact reasons for surgery in these 3 patients were as follows: (i) incomplete resolution after 1 month of anticoagulation therapy; (ii) recurrent ischemic stroke during the admission; and (iii) an initial diagnosis of fibroelastoma that intraoperatively was found to be thrombus. Of these 3 cases, only 1 patient received anticoagulation therapy prior to surgery (for 1 month, with incomplete resolution of the thrombus). For the other 2 patients, whether anticoagulation therapy was started before the surgery is unclear. The remaining 2 patients (9%) underwent surgery for other indications. such as severe mitral stenosis or severe coronary artery disease, during which the thrombus was removed. Three patients benefited from mitral surgery at the time of thrombus resection (1 valvuloplasty and 2 replacements). The indication clearly was due to severe mitral stenosis for only 1 of these 3 patients. The valve and annulus were calcified for the other 2 patients, but no hemodynamically significant stenosis was described in the reports.

**Table 3:** Indications for surgery

Indication	n (%)
Prevention of additional embolic events	3 (14)
Symptomatic mitral stenosis	1 (5)
Other surgical indication—coronary artery disease	1 (5)

% is % of total patients.

### Outcomes

Outcomes are presented in Figure 3. After their initial clinical presentation, thromboembolic events occurred in 6 patients (27%), including 3 patients who had received successful anticoagulation therapy. The most common thromboembolic event was stroke (n = 4; 18%), followed in frequency by coronary embolism (n = 1; 5%), and an embolic event to the eye (n = 1; 5%). The timeline for these events ranged from <1 week to ≤ 5 months from the initial presentation. No in-hospital mortality was reported.

Follow-up care was provided in 16 patients (73%). The median follow-up period was 3 months (IQR: 1-9), with a range from 10 days to 14 months. For those treated with anticoagulation therapy, 23% (n = 5) had persistent thrombus with or without embolization identified on imaging during follow-up care, ranging from 2 weeks to 12 months, with a median of 3 months. One of these patients subsequently underwent surgery. One patient had a recurrent embolic event. All patients treated with surgery had resolution of MAC thrombus. No patient mortality was reported during the follow-up period.

### Quality evaluation

The overall methodological quality level of the case reports included in our systematic review was reasonable, with a low to moderate risk of bias (see Supplemental Table S1). Most cases provided a satisfactory description of the presence of MAC, thrombus attached to MAC, patients’ past medical history, laboratory and imaging tests, management, and outcomes. However, whether most of these case reports represent the authors’ whole experience is unclear. Adequate disease and outcome ascertainment, with consistent investigations, was present in all cases. Follow-up care was described adequately in only half of the case reports (53%), and investigations to identify alternative causes that might explain the observation were reported in only two-thirds of the cases (67%).

## Discussion

Thrombus formation on MAC is a known complication that is associated with thromboembolic events. This systematic review is the first to examine the management and outcomes of MAC thrombus. The 4 main findings (see the graphical abstract) are as follows: (i) the most common clinical presentation for MAC thrombus was a cerebrovascular event; (ii) thromboembolic events after initial presentation were frequent, occurring in 27% of patients (n = 6); (iii) although most patients were managed with anticoagulation therapy alone, a significant number (3 of 22 patients; 14%) required cardiac surgery for recurrent embolization; and (iv) despite anticoagulation therapy, persistent thrombus was observed in 23% of patients (n = 5), at a median follow-up duration of 3 months.

In this systematic review of MAC thrombus, the most common clinical presentation was a cerebrovascular event. MAC itself is known to be a risk factor for ischemic stroke,^8^^,^^21^^,^^50^ with a relative risk of 2.1 reported in the Framingham Heart Study, and a multivariable-adjusted hazard ratio of 1.89 reported in the Strong Heart Study.^8^^,^^21^^,^^22^^,^^51^, ^52^, ^53^ Multiple mechanisms have been proposed for the association between MAC and ischemic stroke, such as concurrent atrial fibrillation, atherosclerosis, and calcific emboli.^23^^,^^24^^,^^39^^,^^54^^,^^55^ For instance, MAC may be associated with a degenerative process affecting mitral leaflet mobility, which could in turn influence thrombus formation, particularly if significant mitral stenosis and left atrial structural and functional abnormalities are present. Thrombus formation on MAC also could be a significant mechanism for MAC’s association with stroke,^39^^,^^55^ and it should be considered in ischemic stroke patients who are found to have MAC, on TTE. In this systematic review, some cases of MAC thrombus were identified only with TEE. Therefore, although TTE is a mainstay of the workup for etiology of ischemic stroke, routine TEE in stroke patients with significant MAC may be indicated, especially in the presence of recurrent events. Thrombus on MAC, particularly on the left atrial side, may be missed on TTE, due to shadowing from the MAC.

We found that patients with MAC thrombus are at risk for thromboembolic events after diagnosis while they are undergoing anticoagulation therapy. However, the treatment strategies were broad, both in terms of regimens (antiplatelet agents and anticoagulation) and duration (ranging from 1 week to lifelong), which may have affected the outcomes. While it is unknown whether patients were within therapeutic range at the time of these events, MAC thrombus may be associated with a persistently elevated thromboembolic risk, both in the short- and mid-term, despite anticoagulation therapy. Furthermore, during follow-up care, persistent thrombus, despite anticoagulation therapy, was found in several patients, and it may be the mechanism for these recurrent embolic events. Thus, MAC thrombus should be considered a high-risk presentation that is associated with a significant incidence of morbidity. Early identification, appropriate management, close monitoring, and follow-up care are key to managing this risk of recurrent embolic events. Repeated imaging to ensure thrombus resolution may be prudent.

Most patients in our review were managed with anticoagulation therapy alone, although a wide variety of anticoagulant regimens were utilized. This situation may have contributed to both the rates of persistent thrombus observed and the number of embolic events that occurred after diagnosis. The ideal anticoagulation therapy regimen and duration remain unclear. Furthermore, delineating the role for use of preventative anticoagulant or antiplatelet therapy for MAC thrombus, particularly in patients with MAC and atrial fibrillation, or with severe MAC, would be beneficial. The ideal timing for a follow-up echocardiogram after initiating anticoagulation therapy also remains to be determined. In most cases, an echocardiogram performed after 4-8 weeks of anticoagulation therapy showed that a partial or complete reduction in thrombus size had occurred. However, some reports demonstrated a reduction in thrombus size within a few days. Given this variability, repeating the echocardiogram 4-8 weeks after achieving therapeutic anticoagulation is reasonable.

Although most patients were managed with anticoagulation therapy alone, a significant number required surgery to prevent recurrent embolization. The indications for surgical management of MAC thrombus without recurrent embolization remain to be elucidated. Surgical treatment of MAC itself is indicated when it results in severe mitral valve stenosis or regurgitation.^56^^,^^57^ However, significant challenges arise with the surgical management of MAC, and by extension, MAC thrombus. Patients who have MAC tend to be of an advanced age and to have significant cardiac and extracardiac comorbidities that may exclude them from surgical candidacy.^57^^,^^58^ Also, multiple anatomic constraints can make surgical MAC management difficult, including the risk of annular disruption, atrioventricular dissociation, and paravalvular regurgitation.^58^ Transcatheter mitral valve replacement in MAC has been associated with a high incidence of morbidity and mortality and would not address the thrombus.^58^ For optimal management, a dedicated multidisciplinary team for MAC surgical management should be involved to determine potential valve solutions.^57^ The indications for surgery for prevention of recurrent embolization of MAC thrombus, however, remain unclear.

### Limitations

Our study, although it provides valuable insights, has several limitations. The systematic literature search identified only 22 patients, despite use of broad search criteria. This small sample size may not represent the management and outcomes of MAC thrombus accurately, due to publication bias and the fact that some data were unavailable or were not reported systematically. Moreover, in some cases, whether competing comorbidities contributed to cerebrovascular events rather than a cardioembolic event is unclear. Confirmation of the diagnosis of MAC thrombus can be challenging. As we included only those cases with appropriate pathology, characteristic imaging, or response to therapeutic anticoagulation, MAC thrombus cases that did not meet these criteria may have been excluded. However, our cases inadvertently could have included those with mobile degenerative debris from MAC or caseous calcification. Additionally, asymptomatic MAC thrombus may not be recognized or described in publications, thereby altering our understanding of the clinical presentation and outcomes of MAC thrombus. Furthermore, the indications for surgery were limited to only 5 patients, a number too small to establish definitive indications for surgery. Finally, the median follow-up period was only 3 months, and adequate follow-up care was reported in only 53% of the case reports. This short duration and inadequacy of long-term surveillance limit our ability to determine the impact of an intervention on patients with MAC, who typically have multiple comorbidities.

### Conclusion

This systematic review is the first to examine the management and outcomes of patients with MAC thrombus. In patients with MAC who present with a thromboembolic event, careful echocardiographic interrogation of MAC to exclude the presence of a thrombus should be performed. The diagnosis of thrombus can be challenging, as the differential can include mobile degenerative debris from MAC or caseous calcification. Diagnosis requires either pathology, supportive imaging, or resolution with treatment. Although thrombus often resolves with anticoagulation therapy, persistent thrombus, despite anticoagulation therapy, and recurrent embolization was observed in this population and may be due to the MAC microenvironment. Although most patients potentially can be managed with anticoagulation therapy alone, some patients may require surgery for recurrent embolization. However, these patients often are highly comorbid, which may complicate the diagnosis and management of those challenging cases. The paucity of studies identified in this systematic review limits our ability to identify a clear management strategy for this potentially underreported pathology. Larger studies on MAC thrombus management are needed to elucidate what constitutes the optimal long-term care for these patients.
