# Supplementary material for: Thrombus on Mitral Annular Calcification: A Systematic Review of Management and Outcomes
Source: CJC Open. 2024 Sep 17;6(12):1538–48. doi: 10.1016/j.cjco.2024.09.001 (PMC11681350; doi:10.1016/j.cjco.2024.09.001)
Supplement: Supplemental Appendix [file mmc1.pdf]

Supplemental Appendix S1: Search strategies. A comprehensive search strategy was developed using a combination of database-specific subject headings and text words for the main concepts of mitral annulus, calcification and thrombus. Results were limited to human studies. No other limits were applied. The following databases were searched on December 15, 2021: Ovid MEDLINE; Ovid Embase; Cochrane Database of Systematic Reviews (Ovid); and Cochrane Central Register of Controlled Trials (Ovid).

Ovid MEDLINE(R) ALL <1946 to December 14, 2021>
Search history sorted by search number ascending

#	Searches	Results	Type
1	Mitral Valve/	31995	Advanced
2	(Mitral adj2 valve*).mp.	73385	Advanced
3	(mitral adj2 annular).mp.	3071	Advanced
4	(mitral adj2 annulus).mp.	3739	Advanced
5	or/1-4	75914	Advanced
6	exp Calcinosis/	47680	Advanced
7	calcification*.mp.	72908	Advanced
8	Calcific.mp.	5929	Advanced
9	calcinos?s.mp.	40272	Advanced
10	or/6-9	96997	Advanced
11	5 and 10	3421	Advanced
12	exp "embolism and thrombosis"/	231683	Advanced
13	thrombos?s.mp.	210556	Advanced
14	thrombus.mp.	42442	Advanced
15	thrombi.mp.	14580	Advanced
16	(blood adj2 clot*).mp.	11495	Advanced
17	thromboembolism*.mp.	62049	Advanced
18	thromboembolic.mp.	31633	Advanced
19	thrombo-embolism*.mp.	851	Advanced
20	thrombo-embolic.mp.	1387	Advanced
21	embolism*.mp.	104728	Advanced
22	embolus.mp.	6167	Advanced
23	emboli.mp.	14086	Advanced
24	or/12-23	372991	Advanced
25	11 and 24	485	Advanced
26	animals/ not (animals/ and humans/)	4897009	Advanced
27	25 not 26	474	Advanced
28	remove duplicates from 27	474	Advanced

Embase <1974 to 2021 December 14>
Search history sorted by search number ascending

#	Searches	Results	Type
1	mitral valve/	26216	Advanced
2	(Mitral adj2 valve*).mp.	108936	Advanced
3	(mitral adj2 annular).mp.	5978	Advanced
4	(mitral adj2 annulus).mp.	7168	Advanced
5	or/1-4	114412	Advanced
6	exp calcification/	94418	Advanced
7	calcification*.mp.	112652	Advanced
8	Calcific.mp.	8413	Advanced
9	calcinos?s.mp.	18604	Advanced
10	or/6-9	128058	Advanced
11	5 and 10	5448	Advanced
12	exp thromboembolism/	567233	Advanced
13	thrombos?s.mp.	377143	Advanced
14	thrombus.mp.	78541	Advanced
15	thrombi.mp.	21294	Advanced
16	(blood adj2 clot*).mp.	251373	Advanced
17	thromboembolism*.mp.	132783	Advanced
18	thromboembolic.mp.	50419	Advanced
19	thrombo-embolism*.mp.	1479	Advanced
20	thrombo-embolic.mp.	2315	Advanced
21	embolism*.mp.	221351	Advanced
22	embolus.mp.	9117	Advanced
23	emboli.mp.	20391	Advanced
24	or/12-23	874236	Advanced
25	11 and 24	878	Advanced
26	(exp animals/ or exp animal experimentation/ or nonhuman/) not ((exp animals/ or exp animal experimentation/ or nonhuman/) and exp human/)	6800979	Advanced
27	25 not 26	858	Advanced
28	remove duplicates from 27	844	Advanced

Cochrane Central Register of Controlled Trials <2014 to Present>
Search history sorted by search number ascending

#	Searches	Results	Type
1	Mitral Valve/	405	Advanced

2	(Mitral adj2 valve*).mp.	2419	Advanced
3	(mitral adj2 annular).mp.	279	Advanced
4	(mitral adj2 annulus).mp.	295	Advanced
5	or/1-4	2784	Advanced
6	exp Calcinosis/	508	Advanced
7	calcification*.mp.	2431	Advanced
8	Calcific.mp.	326	Advanced
9	calcinos?s.mp.	446	Advanced
10	or/6-9	2805	Advanced
11	5 and 10	67	Advanced
12	exp "embolism and thrombosis"/	7413	Advanced
13	thrombos?s.mp.	20553	Advanced
14	thrombus.mp.	2905	Advanced
15	thrombi.mp.	597	Advanced
16	(blood adj2 clot*).mp.	6433	Advanced
17	thromboembolism*.mp.	8971	Advanced
18	thromboembolic.mp.	4703	Advanced
19	thrombo-embolism*.mp.	131	Advanced
20	thrombo-embolic.mp.	207	Advanced
21	embolism*.mp.	8850	Advanced
22	embolus.mp.	297	Advanced
23	emboli.mp.	732	Advanced
24	or/12-23	38893	Advanced
25	11 and 24	3	Advanced
26	remove duplicates from 25	3	Advanced

Cochrane Database of Systematic Reviews <2005 to Present>
Search history sorted by search number ascending

#	Searches	Results	Type
1	(Mitral adj2 valve*).ti,ab.	2	Advanced
2	(mitral adj2 annular).ti,ab.	0	Advanced
3	(mitral adj2 annulus).ti,ab.	0	Advanced
4	or/1-3	2	Advanced
5	calcification*.ti,ab.	6	Advanced
6	Calcific.ti,ab.	4	Advanced
7	calcinos?s.ti,ab.	0	Advanced
8	or/5-7	8	Advanced
9	4 and 8	0	Advanced

10	thrombos?s.ti,ab.	148	Advanced
11	thrombus.ti,ab.	14	Advanced
12	thrombi.ti,ab.	5	Advanced
13	(blood adj2 clot*).ti,ab.	25	Advanced
14	thromboembolism*.ti,ab.	93	Advanced
15	thromboembolic.ti,ab.	66	Advanced
16	thrombo-embolism*.ti,ab.	0	Advanced
17	thrombo-embolic.ti,ab.	2	Advanced
18	embolism*.ti,ab.	73	Advanced
19	embolus.ti,ab.	2	Advanced
20	emboli.ti,ab.	11	Advanced
21	or/10-20	285	Advanced
22	9 and 21	0	Advanced

Supplemental Table S1: Quality assessment of the included studies. This was performed using a modified version of Murad et al.'s approach to methodological quality and synthesis of case series and case reports.

**Study**	**Selection**	**Ascertainment**		**Causality**		**Reporting**	**Evaluation**	
**(first author, country, year)**	**Does the patient(s) represent(s) the whole experience of the investigator (centre) or is the selection method unclear to the extent that other patients with similar presentation may not have been reported?<br><br>(max 1 point)**	**Was the exposure adequately ascertained?<br><br>(max 1 point)**	**Was the outcome adequately ascertained?<br><br>(max 1 point)**	**Were other alternative causes that may explain the observation ruled out?<br><br>(max 1 point)**	**Was follow-up long enough for outcomes to occur?<br><br>(max 1 point)**	**Is the case(s) described with sufficient details to allow other investigators to replicate the research or to allow practitioners make inferences related to their own practice?<br><br>(max 1 point)**	**Total score (max 6 points)**	**Risk of bias**
Stein<br><br>USA, 1995	0	1	1	1	0	1	4	Low to moderate
Malaterre<br><br>France, 1996	0	1	1	1	0	1	4	Low to moderate
Eicher<br><br>France, 1997	1	1	1	1	1	1	6	Low
Shohat-Zobarski<br><br>Israel, 2001	0	1	1	1	0	1	4	Low to moderate
Lahey<br><br>USA, 2002	0	1	1	1	1	1	5	Low
Kawano<br><br>Japan, 2005	0	1	1	1	0	1	4	Low to moderate

Choudry<br>UK, 2009	0	1	1	1	1	1	5	Low
Konishi-Yakushiji<br>Japan, 2010	0	1	1	0	1	1	4	Low to moderate
Sia<br>Canada, 2010	1	1	1	0	0	1	4	Low to moderate
Nagai<br>Japan, 2012	0	1	1	1	1	1	5	Low
Mohan<br>India, 2016	0	1	1	1	1	1	5	Low
Hadadi<br>USA, 2016	0	1	1	0	1	1	4	Low to moderate
Singu<br>Japan, 2017	0	1	1	1	0	1	4	Low to moderate
Suma<br>Italy, 2019	0	1	1	0	1	1	4	Low to moderate
Cherit<br>USA, 2020	0	1	1	0	0	1	3	Moderate

Adapted from Murad et al (2018). **Abbreviations**: NA: not applicable, UK: United Kingdom, USA: United States of America.

Supplemental Table S2: Excluded papers.

	Title	Authors	Published Year	Journal	Volume	Issue	Pages	Reason for exclusion
1	Advantages of open mind commissurotomy using a triple-orifice technique	Aaron, B. L.; Lower, R. R.	1975	Annals of Thoracic Surgery	19	6	654-8	Mitral valve intervention
2	Texture of mitral bioprosthesis, ventricular function and formation of thrombus. Analysis through transesophageal echocardiography and use of bioscore. [Portuguese]	Abensur, H.; Grinberg, M.; Ramires, J. A. F.	2005	Arquivos Brasileiros de Cardiologia	84(5)		410-415	Prosthetic valve
3	Valve replacement in children under 15 years with rheumatic heart disease	Abid, F.; Mzah, N.; el Euch, F.; Ben Ismail, M.	1989	Pediatric Cardiology	10	4	199-204	Rheumatic valve
4	Epidemiological characteristics; clinical and echocardiographic evaluation of rheumatic mitral stenosis in men	Abir Abardazzou, A.; Oummou, S.; Raouah, A.; El Karimi, S.; Benzeroual, D.; El Hattaoui, M.	2017	European Journal of Heart Failure	19(Supplement 1)		572-573	Rheumatic valve
5	Features of patients with severe mitral stenosis with respect to atrial rhythm. Atrial fibrillation in predominant and tight mitral stenosis	Acar, J.; Michel, P. L.; Cormier, B.; Vahanian, A.; Iung, B.	1992	Acta Cardiologica	47	2	115-24	Valve intervention
6	[Should mitral valve diseases, without or with few symptoms, be treated surgically?]	Acar, J.; Vahanian, A.; Michel, P. L.; Luxereau, P.; Cormier, B.; Iung, B.	1992	Archives des Maladies du Coeur et des Vaisseaux	85	12	1837-43	Not MAC thrombus
7	An Observational, Prospective Evaluation of the St Jude Medical Epic Valve	Actrn,	2010	https://trialsearch.who.int/Trial2.aspx?TrialID=ACTRN 12610000023055.				Not MAC thrombus, valve intervention

8	Multiple calcific embolism following mtral valvotomy	Adams, M. J.	1961	British Heart Journal	23		333-6	Valve intervention
9	Expect the unexpected in the matters of the heart	Adhikari, S.; Pandey, S.; Harinstein, M.	2016	Critical Care Medicine	44(12 Supplement 1)		488	Not MAC thrombus
10	Association between mitral annulus calcification and aortic atheroma: a prospective transesophageal echocardiographic study	Adler, Y.; Vaturi, M.; Fink, N.; Tanne, D.; Shapira, Y.; Weisenberg, D.; Sela, N.; Sagie, A.	2000	Atherosclerosis	152	2	451-6	Not MAC thrombus
11	'Calcified clot march' after intravenous thrombolysis	Agarwal, V.; Choudhary, N.; Vyas, S.; Kumar, A.; Goyal, M.	2020	Annals of Indian Academy of Neurology	23(4)		568-570	Not MAC thrombus
12	Bilateral severe valvular regurgitation after me-diastinal radiotherapy in a hodgkin s lymphoma survivor: Radiation does not read text books	Ahmed, A.; Shokr, M.; Rashed, A.; Mishra, T.; Kottam, A.	2018	Journal of General Internal Medicine	33(2 Supplement 1)		478-479	Not MAC thrombus
13	Calcified Amorphous Tumor-Induced Acute Cerebral Infarction	Aizawa, Y.; Nakai, T.; Saito, Y.; Monno, K.; Morikawa, T.; Kogawa, R.; Hatta, T.; Tamaki, T.; Kato, M.; Arimoto, M.; Osaka, S.; Sunagawa, K.; Tang, X. Y.; Tanaka, M.; Hao, H.; Hirayama, A.	2018	International Heart Journal	59	1	240-242	Calcified amorphous tumor, not MAC thrombus
14	Silent brain infarction in patients with rheumatic mitral stenosis	Akdemir, I.; Dagdelen, S.; Yuce, M.; Davutoglu, V.; Akcay, M.; Akdemir, N.;	2002	Japanese Heart Journal	43	2	137-44	Not MAC thrombus

		Aksoy, M.; Erkal, H.; Misirli, H.						
15	The Relationship Between Mitral Annular Calcification, Metabolic Syndrome and Thromboembolic Risk	Aksoy, F.; Guler, S.; Kahraman, F.; Kuyumcu, M. S.; Bagci, A.; Bas, H. A.; Uysal, D.; Varol, E.	2019	Brazilian Journal of Cardiovascular Surgery	34	5	535-541	Not MAC thrombus
16	Leaflet thrombosis after valve-in-valve transcatheter aortic valve implantation: A case series	Aktuerk, D.; Mirsadraee, S.; Quarto, C.; Davies, S.; Duncan, A.; Goliasch, G.; Deharo, P.; Akodad, M.; Biasco, L.; Simovic, S.; Thomson, R.	2020	European Heart Journal - Case Reports	4(4)		06-Jan	Valve intervention
17	TAVR in Patients with a Low STS Score: A Cohort Study with a Mean Follow Up of 2Years	Al-Shaibi, K.; Ahmed, W.; Abukhudair, W.; Nosir, Y.; Al-Shaibi, A.; Kateb, R.; Alasnag, M.	2019	Journal of the American College of Cardiology	2)		S208-S209	Not MAC thrombus
18	Echocardiographic evaluation of porcine bioprosthetic valves: experience with 309 normal and 59 dysfunctioning valves	Alam, M.; Lakier, J. B.; Pickard, S. D.; Goldstein, S.	1983	Cardiovascular Revascularization Medicine	20(8)		695-699	Prosthetic valve
19	Transcatheter Mitral Valve Thrombosis: A Case Report and Literature Review	Alarfaj, M.; Krepp, J.; Jain, T.	2020	American Journal of Cardiology	52	3	309-15	Valve intervention
20	Transcatheter mitral valve thrombosis: A case report and literature review	Alarfaj, M.; Jain, T.; Krepp, J.	2021	Catheterization & Cardiovascular Interventions	97	6	E887-E892	Valve intervention
21	The role of Wnt5b and Wnt11 in aortic valve calcification	Albanese, I.; Yu, B.; You, Z.; Barratt, B.; Shum-Tim, D.; Al Kindi,	2014	Circulation. Conference: American Heart Association	142	SUPPL 3		Not MAC thrombus

		H.; Schwertani, A.; Alreshidan, M.		Scientific Sessions, AHA				
22	Embolic infarct in a known coronary artery disease patient	Ali, A. H.; Alnuaimi, U.; Lacau, S. I.; Badiu, C.; Popescu, A. C.	2020	Arteriosclerosis, Thrombosis, and Vascular Biology. Conference: American Heart Association's Arteriosclerosis, Thrombosis and Vascular Biology	34	SUPP L. 1		No thrombus on MAC
23	Computed Tomography Assessment for Transcatheter Aortic Valve Replacement	Alkhouli, M.; Winkler, L.; Tallaksen, R. J.	2018	European Heart Journal Cardiovascular Imaging	21(Supplement 1)		i957	Not MAC thrombus
24	[Contribution of complementary examinations in the diagnosis and treatment of emboligenic cardiopathies. Retrospective study apropos of 46 cases]	Allard-Latour, G.; Schlama, S.; Aubran, M.; Trigano, J. A.; Juhan, C.; Torresani, J.	1985	Interventional Cardiology Clinics	7(3)		301-313	Not MAC thrombus
25	Ruptured caseous calcification of the mitral annulus	Allwood, R. P.; Bonacci, E. L.; McKinnon, E.	2021	Archives des Maladies du Coeur et des Vaisseaux	78	8	1249-54	Not MAC thrombus
26	Percutaneous transvenous mitral commissurotomy in 71 years old woman with mitral stenosis	Al Shafi Majumder, A.	2014	Australasian Journal of Ultrasound in Medicine	24	2	106-111	Not MAC thrombus
27	The Bjork-Shiley valve prosthesis. Analysis of long-term evolution	Alvarez, L.; Escudero, C.; Figuera, D.; Castillo-Olivares, J. L.	1992	Chest	150(4 Supplement 1)		92A	Prosthetic valve
28	Non-bacterial thrombotic endocarditis in a patient with antiphospholipid syndrome	Alvarez, C. S.; Blackshear, J.	2016	Journal of Thoracic &	104	5	1249-58	Not MAC thrombus

				Cardiovascular Surgery				
29	An extremely severe case of Aicardi-Goutieres syndrome 7 with a novel variant in IFIH1	Amari, S.; Tsukamoto, K.; Ishiguro, A.; Yanagi, K.; Kaname, T.; Ito, Y.	2020	European Journal of Medical Genetics	63(2) (no pagination)			Not MAC thrombus
30	Prognostic impact, imaging and clinical predictors of mitral regurgitation improvement after TAVI in aortic stenosis patients: A multicentric study	Amat-Santos, I. J.; Cortes, C.; Nombela-Franco, L.; Munoz-Garcia, A. J.; Gutierrez-Ibanes, E.; Soriano, J. G. C.; Macaya, C.; Jimenez-Mazuecos, J.; De La Torre Hernandez, J. M.	2015	Journal of the American College of Cardiology	1)		B293	Valve intervention
31	Wilkins' score as predictor of thromboembolic events in rheumatic mitral stenosis and normal sinus rhythm	Amin, A. E. H.; Farrag, A. A. F.; Ammar, W. A. A.; Elarousy, W. A. E.	2014	European Heart Journal	1)		122	Rheumatic valve disease
32	Review on Future Targets and Current Trends in Transcatheter Left Atrial Appendage Closure	Amoroso, N. S.	2019	Current Cardiovascular Risk Reports	13(6) (no pagination)			Not MAC thrombus
33	Long-term follow-up of aortic valve replacement with the fresh aortic homograft	Anderson, E. T.; Hancock, E. W.	1976	Journal of Thoracic & Cardiovascular Surgery	72	1	150-6	Prosthetic valve
34	The Angell-Shiley porcine xenograft	Angell, W. W.; Angell, J. D.; Sywak, A.	1979	Journal of Thoracic & Cardiovascular Surgery	83	4	493-502	Prosthetic valve
35	Twelve-year experience with glutaraldehyde-preserved porcine xenografts	Angell, W. W.; Angell, J. D.; Kosek, J. C.	1982	Annals of Thoracic Surgery	28	6	537-53	Prosthetic valve

36	Indications for closed- or open-heart surgery for mitral stenosis. Review of 152 operated cases	Ankeney, J. L.	1967	Annals of Thoracic Surgery	3	5	389-405	Not MAC thrombus
37	Case records of the Massachusetts General Hospital. Weekly clinicopathological exercises. Case 29-1971	Anonymous,	1971	New England Journal of Medicine	285	4	220-8	Publication before 1985
38	Cardiogenic brain embolism. Cerebral Embolism Task Force	Anonymous,	1986	Archives of Neurology	43	1	71-84	Not MAC thrombus
39	Correction: Transcatheter Mitral Valve Replacement for Patients With Symptomatic Mitral Regurgitation: A Global Feasibility Trial (Journal of the American College of Cardiology (2017) 69(4) (381-391)(S0735109716371224)(10.1016/j.jacc.2016.10.068))	Anonymous,	2017	Journal of the American College of Cardiology	69(9)		1213	Not MAC thrombus
40	Conservative mitral valve surgery: has the progress changed the indications?	Antunes, M. J.	1991	Schweizerische Medizinische Wochenschrift. Journal Suisse de Medecine	121	51-52	1946-53	Not MAC thrombus
41	Right atrial thrombus associated with combined valvular disease: case report	Aoyagi, S.; Nishimi, M.; Hiratsuka, R.; Takaseya, T.; Teshima, H.	2001	Journal of Heart Valve Disease	10	4	542-4	Not MAC thrombus
42	Re-repair of the failed mitral valve: insights into aetiology and surgical management	Aphram, G.; De Kerchove, L.; Mastrobuoni, S.; Navarra, E.; Solari, S.; Tamer, S.; Baert, J.; Poncelet, A.; Rubay, J.; Astarci, P.; Noirhomme, P.; El Khoury, G.	2018	European Journal of Cardio-Thoracic Surgery	54	4	774-780	Mitral valve intervention

43	Calcific aortic-valve stenosis and angiodysplasia of the colon: Heyde's syndrome - Report of two cases	Apostolakis, E.; Doering, C.; Kantartzis, M.; Winter, J.; Schulte, H. D.	1990	Thoracic and Cardiovascular Surgeon	38(6)		374-376	Not MAC thrombus
44	Acute cardioembolic cerebral infarction: answers to clinical questions	Arboix, A.; Alio, J.	2012	Current cardiology reviews	8	1	54-67	Not MAC thrombus
45	Acute onset pulmonary edema after cusp tear of mitral bioprosthesis	Ardal, H.; Yilmaz, O.; Susam, M.; Arbatli, H.; Yagan, N. E.; Can, E.; Sonmez, B.	2010	Interactive Cardiovascular and Thoracic Surgery	1)		S183	Prosthetic valve
46	Mitral annular calcification and thromboembolic stroke	Aronow, W. S.	1990	Journal of Cardiovascular Technology	9(3)		193-195	No thrombus on MAC
47	Association of mitral anular calcium with new thromboembolic stroke and cardiac events at 39-month follow-up in elderly patients	Aronow, W. S.; Koenigsberg, M.; Kronzon, I.; Gutstein, H.	1990	Geriatrics	46	4	73-5, 79	No thrombus on MAC
48	Mitral annular calcification: significant and worth acting upon	Aronow, W. S.	1991	Herz	16	6	395-404	Not MAC thrombus
49	Etiology and pathogenesis of thromboembolism	Aronow, W. S.	1991	American Journal of Cardiology	81	1	105-6	Not MAC thrombus
50	Frequency of thromboembolic stroke in persons greater than or equal to 60 years of age with extracranial carotid arterial disease and/or mitral annular calcium	Aronow, W. S.; Schoenfeld, M. R.; Gutstein, H.	1992	American Journal of Cardiology	85	5	672-3, A11	No thrombus on MAC
51	Association of mitral annular calcium with new thromboembolic stroke at 44-month follow-up of 2,148 persons, mean age 81 years	Aronow, W. S.; Ahn, C.; Kronzon, I.; Gutstein, H.	1998	American Journal of Cardiology	65	22	1511-2	No thrombus on MAC

52	Association of mitral annular calcium with prior thromboembolic stroke in older White, African-American, and Hispanic men and women	Aronow, W. S.; Ahn, C.; Kronzon, I.; Gutstein, H.	2000	American Journal of Cardiology	70	1	123-4	No thrombus on MAC
53	Role of multimodality imaging in evaluation left atrial masses: Our experience & analysis with a case series	Arunachalam, S. K.; G, G.; S, V.	2018	Indian Heart Journal	70(Supplement 2)		S101	Thrombus on rheumatic valve
54	A novel device for endovascular native aortic valve resection for transapical transcatheter aortic valve implantation	Astarci, P.	2011	Interactive Cardiovascular and Thoracic Surgery	2)		S98	Valve intervention
55	Impaired fibrinolytic capacity in rheumatic mitral stenosis with or without atrial fibrillation and nonrheumatic atrial fibrillation	Atalar, E.; Ozmen, F.; Haznedaroglu, I.; Ozer, N.; Aksoyek, S.; Ovunc, K.; Nazli, N.; Kirazli, S.; Kes, S.	2002	International Journal of Hematology	76	2	192-5	Rheumatic valve, not MAC thrombus
56	Septic coronary embolism presenting as STEMI in a patient with cardiovascular risk factors	Atreya, A. R.; Mallidi, J.; Egan, T.; Kugelmaas, A.	2015	Journal of the American College of Cardiology	1)		A672	Not MAC thrombus
57	Mitral valve replacement in children with rheumatic heart disease	Attie, F.; Kuri, J.; Zanoniani, C.; Renteria, V.; Buendia, A.; Ovseyevitz, J.; Lopez-Soriano, F.; Garcia-Cornejo, M.; Martinez-Rios, M. A.	1981	Circulation	64(4)		812-817	Prosthetic valve, rheumatic heart disease
58	Carpentier-Edwards pericardial valves in the mitral position: ten-year follow-up	Aupart, M. R.; Neville, P. H.; Hammami, S.; Sirinelli, A. L.; Meurisse, Y. A.; Marchand, M. A.	1997	Journal of Thoracic & Cardiovascular Surgery	113	3	492-8	Prosthetic valve

59	Influence of pregnancy after bioprosthetic valve replacement in young women: a prospective five-year study	Avila, W. S.; Rossi, E. G.; Grinberg, M.; Ramires, J. A.	2002	Journal of Heart Valve Disease	11	6	864-9	Prosthetic valve
60	A retrospective analysis of factors influencing re-operation in patients undergoing mechanical valve replacement	Aydin, E.; Yapici, F.	2013	Cardiovascular Journal of Africa	24(7)		251-254	Prosthetic valve
61	Severe systemic calciphylaxis with culture-negative endocarditis	Azegami, T.; Wakino, S.; Hayashi, M.; Itoh, H.	2013	Clinical Kidney Journal	6(3)		342-343	Not MAC thrombus
62	An uncommon reason for transient ischemic attack: Nonbacterial thrombotic endocarditis	Babur Guler, G.; Kilicgedik, A.; Zencirkiran Agus, H.; Kahveci, G.	2020	European Heart Journal Cardiovascular Imaging	21(Supplement 1)		i530	Not MAC thrombus
63	Medium term results with the Ionescu-Shiley bioprosthesis in 98 patients. [French]	Bachet, J.; Farncoual, M.; Landau, J. F.	1984	Archives des Maladies du Coeur et des Vaisseaux	77(8)		946-952	Prosthetic valve
64	Massive Left Atrial Thrombi During Dabigatran Therapy for Nonvalvular Atrial Fibrillation	Bachvarova, V.; Bucci, C.; Ho, N.; Yazdan-Ashoori, P.; Morgan, C.	2017	Case	1(5)		198-201	Not MAC
65	Calcific arterial embolization accompanying commissurotomy; report of a proved case	Baglio, C. M.; Hunter, W. C.	1959	Journal of Thoracic Surgery	37	4	490-5	Mitral valve intervention
66	Reconstruction of the cardiac valves with autologous tissue	Bailey, C. P.; Zimmerman, J.; Hirose, T. T.; Folk, F. S.; Bakst, A. A.	1976	Vascular Surgery	10	2	99-119	Not MAC thrombus
67	Rapid Bioprosthetic Valve Degeneration Resulting in Severe Mitral Stenosis	Bajaj, M.; Abuissa, H.; Main, M. L.	2008	Journal of the American Society of Echocardiography	21(1)		e1	Not MAC thrombus

68	Caseous Necrosis of Mitral Annulus	Balci, S.; Akkaya, S.; Ardali, S.; Hazirolan, T.	2015	Case Reports in Radiology	2015		561329	Not MAC thrombus
69	PCI in impending cardiogenic shock tackling multiple culprit lesions	Bali, H. K.; Chauhan, H.	2015	Journal of the American College of Cardiology	1)		S262-S263	Not MAC thrombus
70	Mean platelet volume and mitral annular calcification	Balta, S.; Demirkol, S.; Cakar, M.; Kurt, O.; Unlu, M.; Kucuk, U.	2013	Blood Coagulation and Fibrinolysis	24(8)		899	Not MAC thrombus
71	Acute renal infarction secondary to calcific embolus from mitral annular calcification	Bande, D.; Abbara, S.; Kalva, S. P.	2011	Cardiovascular & Interventional Radiology	34	3	647-9	No thrombus on MAC
72	A few issues involving the maze procedure	Banerjee, A.; Akhter, M.; Khanna, S. K.	1995	Annals of Thoracic Surgery	59	6	1623-4	Not MAC thrombus
73	A case report of malignant primary pericardial mesothelioma with atypical imaging appearance: Multimodality imaging with histopathological correlation	Banisauskaite, A.; Jankauskas, A.; Sarauskas, V.; Arzanauskaite, M.	2020	European Heart Journal - Case Reports	4(2)		05-Jan	Not MAC thrombus
74	Echocardiography in diagnostic assessment of peripheral arterial embolization	Bar-El, Y.; Adar, R.; Schneiderman, Y.; Motro, M.	1990	American Heart Journal	119	5	1090-4	No thrombus on MAC
75	Aortic sinotubular atherosclerotic debris associated with cerebral embolic events can be identified by transthoracic echocardiography	Barasch, E.; Kaushik, V.; Ahn, C.	1998	Cardiology	90	4	253-7	Not MAC thrombus
76	Unexpected detection of nonbacterial thrombotic endocarditis on mitral valve in a patient with atypical chest pain	Barbati, G.; Erente, G.; Marotta, C.; Ramondo, A. B.	2017	European Heart Journal Cardiovascular Imaging	18(Supplement 3)		iii366	Not MAC thrombus
77	Notes on atrial thrombosis in mitral stenosis. [Italian]	Barberis, L.; Passerone, G. C.; Lijoi, A.;	1978	Minerva Cardioangiologica	26(1-2)		59-62	Publication before 1985

		Brisighella, A.; Venere, G.						
78	Heart in ischemic stroke--a changing emphasis	Barnett, H. J.	1983	Neurologic Clinics	1	1	291-315	Not MAC thrombus
79	Avoidance of embolic complications by ultrasonic characterization of the ascending aorta	Barzilai, B.; Marshall, W. G., Jr.; Saffitz, J. E.; Kouchoukos, N.	1989	Circulation	80	3 Pt 1	I275-9	No MAC
80	Iatrogenic deaths following treatment for hypertrophic obstructive cardiomyopathy: case reports and an approach to the autopsy and death certification	Batalis, N. I.; Harley, R. A.; Collins, K. A.	2005	American Journal of Forensic Medicine & Pathology	26	4	343-8	Not MAC thrombus
81	The diagnosis of left atrial thrombus by cineangiography	Baue, A. E.; Baum, S.; Wallace, H. W.; Blakemore, W. S.; Zinsser, H. F.	1968	Archives of Surgery	97	6	976-83	Not MAC
82	[Iconographic cases of cardiovascular pathology not frequently encountered]	Bazzi, A.; De Candia, M.; Lutman, M.; Zanuttini, D.	1969	Quaderni di Radiologia	35	2	103-22	Publication before 1985
83	Balloon valvuloplasty in congenital and acquired heart disease: morphologic considerations	Becker, A. E.; Hoedemaker, G.	1987	Zeitschrift fur Kardiologie	76 Suppl 6		Sep-73	Not MAC thrombus
84	Extensive left atrial tear during mitral valve replacement: A word of caution	Bedi, H. S.; Kalkat, M. S.; Nayyar, A.	2001	Asian Cardiovascular and Thoracic Annals	9(2)		123-124	Surgical intervention
85	Food for Thought: Mitral Stenosis with a Side of Mac and Cheese	Behari, G.; Jenssen, F.; Price, A.; Arcement, L.; Vyas, V.	2015	Journal of the Louisiana State Medical Society	167	3	156-7	Not MAC thrombus
86	Restenosis of the mitral valve	Belcher, J. R.	1979	Annals of the Royal College of Surgeons of England	61(4)		258-264	Not MAC thrombus, surgical intervention

87	Radiation induced valve disease and cardiotoxicity induced heart failure is frequent	Bergler-Klein, J.; Schneider, M.; Frey, M.; Gyongyosi, M.; Binder, T.	2018	European Heart Journal Cardiovascular Imaging	20(Supplement 1)		i442	No thrombus
88	Radiation induced valve disease and cardiotoxicity induced cardiomyopathy is frequent	Bergler-Klein, J.; Badr-Eslam, R.; Frey, M.; Schneider, M.; Pavone-Gyongyosi, M.; Binder, T.	2019	European Journal of Heart Failure	20(Supplement 1)		220	No thrombus
89	Balloon mitral valvotomy by using the Twin-AT catheter: Immediate results and complications in 110 patients	Berland, J.; Rocha, P.; Choussat, A.; Lefebvre, T.; Fernandez, F.; Rath, P.	1993	Catheterization and Cardiovascular Diagnosis	28(2)		126-133	No MAC thrombus before surgery
90	Percutaneous mitral valvulotomy in non-optimal candidates	Bernard, Y.; Bassand, J. P.; Schiele, F.; Anguenot, T.; Payet, M.; Abdou, S.; Maurat, J. P.	1991	European Heart Journal	12(SUPPL. B)		90-94	No MAC thrombus
91	Results of up to 9 years of high-temperature-fixed valvular bioprostheses in a young population	Berrebi, A. J.; Carpentier, S. M.; Phan, K. P.; Nguyen, V. P.; Chauvaud, S. M.; Carpentier, A.	2001	Annals of Thoracic Surgery	71	5 Suppl	S353-5	No MAC thrombus, artificial valve
92	Arteriolar vs. valvular thrombosis: Pick your evil!	Berry, A.; Degheim, G.; Saba, S.	2018	Thrombosis Journal	16(1) (no pagination)			Mechanical valve
93	Mitral Annular Calcification and Calcific Mitral Stenosis: Therapeutic Challenges and Considerations	Bertrand, P. B.; Mihos, C. G.; Yucel, E.	2019	Current Treatment Options in Cardiovascular Medicine	21(4) (no pagination)			No thrombus
94	The burden of post-actinic heart disease: A case of severe valvular	Bettella, N.; Previtero, M.; Ruocco, A.;	2020	European Heart Journal	21(Supplement 1)		i45	Not MAC thrombus

	and coronary artery disease in a cancer survivor	Muraru, D.; Iliceto, S.; Badano, L. P.		Cardiovascular Imaging				
95	Long-Term Outcomes of Mosaic Versus Perimount Mitral Replacements: 17-Year Follow-Up of 940 Implants	Beute, T. J.; Goehler, M.; Parker, J.; Boeve, T.; Heiser, J.; Murphy, E.; Timek, T.; Willekes, C. L.	2020	Annals of Thoracic Surgery	110(2)		508-515	Prosthetic valve
96	Cardiac Calcified Amorphous Tumor in a Newborn	Bhag, G.; Kumar, G.; Sahai, K.; Arora, H. S.; Sharma, V. K.	2018	Annals of Thoracic Surgery	106	1	e27-e28	Not MAC
97	Clot Histopathology in Ischemic Stroke with Infective Endocarditis	Bhaskar, S.; Saab, J.; Cappelen-Smith, C.; Killingsworth, M.; Wu, X. J.; Cheung, A.; Manning, N.; Aouad, P.; McDougall, A.; Hodgkinson, S.; Cordato, D.	2019	Canadian Journal of Neurological Sciences	46(3)		331-336	No MAC thrombus
98	Warfarin in hemodialysis patients: More harm than good?	Bhatta, M.	2016	Journal of General Internal Medicine	1)		S782	Not MAC thrombus
99	An Unusual Cause of Embolic Stroke in a Patient with Rheumatoid Arthritis	Bhattacharya, A.; Astsaturov, A.; Lam, U.	2021	Journal of the American College of Cardiology	77(18 Supplement 1)		2644	Not MAC thrombus
100	Mitral stenosis in the young in developing countries	Bhayana, J. N.; Khanna, S. K.; Gupta, B. K.; Sharma, S. R.; Gupta, M. P.; Padmavati, S.	1974	Journal of Thoracic and Cardiovascular Surgery	68(1)		126-130	Valve intervention

101	Repair of mitral regurgitation from myxomatous degeneration in the patient with a severely calcified posterior annulus	Bichell, D. P.; Adams, D. H.; Aranki, S. F.; Rizzo, R. J.; Cohn, L. H.	1995	Journal of Cardiac Surgery	10	4 Pt 1	281-4	No MAC thrombus
102	Differential calcification of cusps and aortic wall of failed stented porcine bioprosthetic valves	Biedrzycki, L. M.; Lerner, E.; Levy, R. J.; Schoen, F. J.	1997	Journal of Biomedical Materials Research	34	4	411-5	Prosthetic valve
103	Percutaneous mitral and aortic paravalvular leak repair: Indications, current application, and future directions	Binder, R. K.; Webb, J. G.	2013	Current Cardiology Reports	15(3) (no pagination)			Valve intervention
104	Cardiac myxomas in patients over 75 years of age: A series of 19 cases. [French]	Bire, F.; Roudaut, R.; Chevalier, J. M.; Quiniou, G.; Dubecq, S.; Marazanoff, M.; Choussat, A.	1999	Archives des Maladies du Coeur et des Vaisseaux	92(3)		323-328	Not MAC thrombus
105	Role of closed mitral commissurotomy for mitral restenosis	Biswas, B.; Datta, S.; Dutta, A. L.; Chakraborty, A.	1999	Journal of the Indian Medical Association	97	7	255-8	Rheumatic valve
106	Aortic plaque in atrial fibrillation: prevalence, predictors, and thromboembolic implications	Blackshear, J. L.; Pearce, L. A.; Hart, R. G.; Zabalgoitia, M.; Labovitz, A.; Asinger, R. W.; Halperin, J. L.	1999	Stroke	30	4	834-40	Not MAC thrombus
107	Percutaneous mitral balloon valvotomy	Block, P. C.; Tuzcu, E. M.; Palacios, I. F.	1991	Cardiology Clinics	9	2	271-87	Not MAC thrombus
108	Value of echocardiography and Holter monitoring in the diagnosis of occult sources of cerebral emboli. [French]	Boissonnot, L.; Herpin, D.; Allal, J.	1985	Annales de Cardiologie et d'Angeiologie	34(8)		541-545	No MAC thrombus
109	Failure of Hancock xenograft valve: importance of valve position (4- to 9-year follow-up)	Bolooki, H.; Mallon, S.; Kaiser,	1983	Annals of Thoracic Surgery	36	3	246-52	Prosthetic valve

		G. A.; Thurer, R. J.; Kieval, J.						
110	Calcific emboli complicating mitral valve commissurotomy; report of a case	Bolton, H. E.; Maniglia, R.; Massey, F. C.	1952	Journal of Thoracic Surgery	24	5	502-9	Valve intervention
111	A case report: Giant left atrial thrombus	Borazan, E.; Ercan, E.; Kocogullari, C. U.; Melek, M.	2011	International Journal of Cardiology	2)		S160	Previous valve intervention
112	Stroke - Heart and brain relationship	Bornstein, N. M.	1998	Acta Clinica Croatica, Supplement	37(1)		25-27	Unlikely MAC thrombus
113	Intermittent obstruction of the Omnicarbon-valve prosthesis in the mitral position due to interference by papillary muscle. Diagnostic and surgical considerations	Borowski, A.; Reiss, N.; Klaer, R.	1992	Journal of Cardiovascular Surgery	33	3	305-7	Prosthetic valve
114	[Structural changes of Hancock bioprostheses in children (author's transl)]	Bortolotti, U.; Milano, A.; Mazzucco, A.; Callucci, V.; Valente, M.; Del Maschio, A.; Valfre, C.; Thiene, G.	1980	Journal of the American College of Cardiology	24	3	676-82	Prosthetic valve
115	Evidence of impending embolization of a calcific cusp fragment from a mitral porcine xenograft	Bortolotti, U.; Milano, A.; Thiene, G.; Valente, M.; Mazzucco, A.; Gallucci, V.	1982	Giornale Italiano di Cardiologia	10	11	1520-5	Prosthetic valve
116	Results of reoperation for primary tissue failure of porcine bioprostheses	Bortolotti, U.; Milano, A.; Mazzucco, A.; Valfre, C.; Talenti, E.; Guerra, F.; Thiene, G.; Gallucci, V.	1985	Annals of Thoracic Surgery	50	5	734-8	Prosthetic valve
117	Long-term durability of the Hancock porcine bioprosthesis	Bortolotti, U.; Milano, A.; Thiene,	1987	Journal of Thoracic &	90	4	564-9	Prosthetic valve

	following combined mitral and aortic valve replacement: an 11-year experience	G.; Guerra, F.; Mazzucco, A.; Talenti, E.; Gallucci, V.		Cardiovascular Surgery				
118	Influence of prosthetic design on durability of the Liotta porcine valve in the mitral position	Bortolotti, U.; Milano, A.; Mazzucco, A.; Guerra, F.; Stellin, G.; Talenti, E.; Thiene, G.; Gallucci, V.	1990	Annals of Thoracic Surgery	44	2	139-44	Prosthetic valve
119	Hancock II porcine bioprosthesis: excellent durability at intermediate-term follow-up	Bortolotti, U.; Milano, A.; Mazzaro, E.; Thiene, G.; Talenti, E.; Casarotto, D.	1994	Thoracic & Cardiovascular Surgeon	30	6	405-6	Prosthetic valve
120	Minimally invasive approach for redo mitral valve surgery	Botta, L.; Cannata, A.; Bruschi, G.; Fratto, P.; Taglieri, C.; Russo, C. F.; Martinelli, L.	2013	Journal of Thoracic Disease	5(SUPP L.6)		S686-S693	Prior mitral valve intervention
121	Mid-term follow-up in patients with Biocor porcine bioprostheses	Bottio, T.; Rizzoli, G.; Gerosa, G.; Thiene, G.; Casarotto, D.	2002	Cardiovascular Surgery	10	3	238-44	Prosthetic valve
122	Late results of percutaneous mitral commissurotomy up to 20 years: development and validation of a risk score predicting late functional results from a series of 912 patients	Bouleti, C.; Iung, B.; Laouenan, C.; Himbert, D.; Brochet, E.; Messika-Zeitoun, D.; Detaint, D.; Garbarz, E.; Cormier, B.; Michel, P. L.; Mentre, F.; Vahanian, A.	2012	Heart	99(18)		1336-1341	Mitral valve intervention
123	Long-term efficacy of percutaneous mitral commissurotomy for restenosis	Bouleti, C.; Iung, B.; Himbert, D.; Brochet, E.; Messika-Zeitoun, D.; Detaint, D.;	2013	Circulation	125	17	2119-27	Previous mitral valve intervention

	after previous mitral commissurotomy	Garbarz, E.; Cormier, B.; Vahanian, A.						
124	Simplified surgical technique for the management of severe mitral annulus calcification	Bowers, P.; Mathur, M.	2017	Heart Lung and Circulation	26(Supplement 3)		S356-S357	No MAC thrombus
125	Annular injuries following the insertion of heart valve prostheses	Bowes, V. F.; Datta, B. N.; Silver, M. D.; Minielly, J. A.	1974	Thorax	29	5	530-3	Mitral valve intervention
126	Anticoagulation for atrial fibrillation and stroke prevention	Boysen, G.	1993	Neuroepidemiology	12	5	280-4	No MAC thrombus
127	IMC bovine pericardial valve: 11 years	Braile, D. M.; Ardito, R. V.; Greco, O. T.; Lorga, A. M.	1991	Journal of Cardiac Surgery	6	4 Suppl	580-8	Prosthetic valve
128	The PB IMC bovine pericardial prosthesis: 11 years in the mitral position. Instituto de Molestias Cardiovasculares. [Portuguese]	Braile, D. M.; Zaiantchick, M.; Souza, D. R.	1992	Arquivos brasileiros de cardiologia	59(1)		13-21	Prosthetic valve
129	Dyspnoea, fatigue and an elusive cardiac mass: An echocardiographic approach	Bras, D.; Guerreiro, R. A.; Congo, K.; Pais, J.; Carvalho, J.; Semedo, P.; Neves, D.; Santos, A. R.; Aguiar, J.	2017	European Heart Journal Cardiovascular Imaging	18(Supplement 3)		iii366-iii367	Not MAC thrombus
130	Characteristics and outcomes of patients with at rial fibrillation and significant valvular lesions: Experience from the rocke T AF trial	Breithardt, G.; Baumgartner, H.; Berkowitz, S.; Hellkamp, A.; Piccini, J.; Stevens, S.; Lokhnygina, Y.; Patel, M.; Halperin, J.; Singer, D.; Hankey, G.; Hacke, W.; Becker, R.; Nessel, C.;	2013	Journal of the American College of Cardiology	1)		E282	Not MAC thrombus

		Mahaffey, K.; Robert, K. F.						
131	Cardiac involvement in patients with primary antiphospholipid syndrome	Brenner, B.; Blumenfeld, Z.; Markiewicz, W.; Reisner, S. A.	1991	Journal of the American College of Cardiology	18(4)		931-936	No thrombus on MAC
132	Catheter Management Of Mitral Stenosis	Brown, K. N.; Kanmanthareddy, A.	2020	StatPearls Publishing	1		1	Not MAC thrombus
133	Mitral mass in patient with heart failure: Cancer, thrombosis, vegetation or. big mac?	Buccolieri, M.; Cardona, A.; Cosmi, D.; Piccioni, N.; Lemmi, A.; Murrone, A.	2021	European Heart Journal, Supplement	23(SUP PL C)		C46	No thrombus on MAC
134	Stroke due to spontaneous calcified cerebral embolus as presenting feature of calcified aortic stenosis. [French]	Bugnicourt, J. M.; Bonnaire, B.; Lepage, L.; Garcia, P. Y.; Lefranc, M.; Godefroy, O.	2008	Journal des Maladies Vasculaires	33(2)		106-109	Not MAC thrombus
135	Transcatheter Heart Valves: A Biomaterials Perspective	Bui, H. T.; Khair, N.; Yeats, B.; Gooden, S.; James, S. P.; Dasi, L. P.	2021	Advanced Healthcare Materials	10(15) (no pagination)			Not MAC thrombus
136	Treatment of mitral stenosis	Burckhardt, D.; Hoffmann, A.; Kiowski, W.	1991	European Heart Journal	12 Suppl B		Aug-95	Not MAC thrombus
137	Morphologic findings in explanted Hancock II porcine bioprostheses	Butany, J.; Yu, W.; Silver, M. D.; David, T. E.	1999	Journal of Heart Valve Disease	8(1)		15-Apr	Prosthetic valve
138	Inflammation and infection in nine surgically explanted Medtronic Freestyle stentless aortic valves	Butany, J.; Zhou, T.; Leong, S. W.; Cunningham, K. S.; Thangaroopan, M.; Jegatheeswaran,	2007	Cardiovascular Pathology	16	5	258-67	Prosthetic valve

		A.; Feindel, C.; David, T. E.						
139	Percutaneous mitral repair in the setting of severe mitral annular calcification: Midterm results from a single centre experience	Buzzatti, N.; Denti, P.; De Bonis, M.; Schiavi, D.; Vicentini, L.; Latib, A.; Colombo, A.; Di Giannuario, G.; La Canna, G.; Alfieri, O.	2015	EuroIntervention. Conference: EuroPCR		pagina tion		No thrombus
140	Two cases of caseous calcification of the Mitral Annulus	Byeon, K. M.; Park, S. W.; Choi, J. O.; Yang, J. H.; Han, H. J.; Son, G. H.; Kim, Y. J.	2009	Korean Circulation Journal	39(2)		82-85	No thrombus
141	Twelve-year follow up with the Sorin Pericarbon bioprosthesis in the mitral position	Caimmi, P. P.; Di Summa, M.; Galloni, M.; Gastaldi, L.; Papillo, B.; Actis Dato, G. M.; Agaccio, G.; Donegani, E.; Poletti, G.; Morea, M.	1998	Journal of Heart Valve Disease	7	4	400-6	No MAC
142	Caseous calcification of the mitral annulus: A silent cause of intracardiac mass	Campos, I.; Galvao Braga, C.; Costeira-Pereira, A.; Salome, N.; Vieira, C.; Pereira, V. H.; Costa-Oliveira, C.; Marques Pires, C.; Medeiros, P.; Flores, R.; Mane, F.; Marques, J.	2020	European Heart Journal Cardiovascular Imaging	21(Supplement 1)		i98	No thrombus
143	Infective endocarditis and caseous calcification of the mitral annulus: the odd couple	Capin, E.; Leon, D.; Rodriguez, M. L.; Corros, C.; Garcia-Campos, A.; de la Hera, J.; Martin, M.	2014	Archivos de Cardiologia de Mexico	84	2	100-1	No thrombus

144	Cerebral embolism in the Michael Reese Stroke Registry	Caplan, L. R.; Hier, D. B.; D'Cruz, I.	1983	Stroke	14	4	530-6	Not MAC thrombus
145	Specific cardiac disorders in 402 consecutive patients with ischaemic cardioembolic stroke	Capmany, R. P.; Arboix, A.; Casanas-Munoz, R.; Anguera-Ferrando, N.	2004	International Journal of Cardiology	95(2-3)		129-134	Not MAC thrombus
146	Extensive calcification of the mitral valve anulus: pathology and surgical management	Carpentier, A. F.; Pellerin, M.; Fuzellier, J. F.; Relland, J. Y.	1996	Journal of Thoracic & Cardiovascular Surgery	111	4	718-29; discussion 729	No thrombus
147	Percutaneous mitral balloon valvotomy and the new demographics of mitral stenosis	Carroll, J. D.; Feldman, T.	1993	Journal of the American Medical Association	270(14)		1731-1736	No thrombus
148	Role of echocardiography in percutaneous mitral valve interventions	Cavalcante, J. L.; Rodriguez, L. L.; Kapadia, S.; Tuzcu, E. M.; Stewart, W. J.	2012	JACC: Cardiovascular Imaging	5(7)		733-746	No MAC
149	The evolving role and use of echocardiography in the evaluation of cardiac source of embolism	Celeste, F.; Muratori, M.; Mapelli, M.; Pepi, M.	2017	Journal of Cardiovascular Echography	27(2)		33-44	Perhaps may have MAC thrombus
150	The Mosaic Mitral Valve Bioprosthesis: A Long-Term Clinical and Hemodynamic Follow-Up	Celiento, M.; Blasi, S.; De Martino, A.; Pratali, S.; Milano, A. D.; Bortolotti, U.	2016	Texas Heart Institute Journal	43	1	13-Sep	Prosthetic valve
151	A fatal condition in the thoracic aorta	Celikyay, F.; Yuksekkaya, R.; Yilmaz, A.; Beyhan, M.; Sivqin, H.; Almus, F.	2013	Annals of Saudi Medicine	33(5)		508-509	No MAC, thrombus not on MAC
152	Two giant thrombi in the left atrium	Ceyhan, K.; Karayakali, M.; Koc, F.	2010	Turk Kardiyoloji Dernegi Arsivi	38(1)		71	Rheumatic valve

153	Mechanical valve in aortic position is a valid option in children and adolescents	Champsaur, G.; Robin, J.; Tronc, F.; Curtil, A.; Ninet, J.; Sassolas, F.; Vedrinne, C.; Bozio, A.	1997	European Journal of Cardio-Thoracic Surgery	11	1	117-22	Prosthetic valve
154	Relationship between bio markers and intra cardiaccal cification in patients with mild to moderate aortic stenosis	Chan, K.; Tam, J.; Dumesnil, J. G.; Teo, K. K.	2011	Canadian Journal of Cardiology	1)		S105	No MAC thrombus
155	Calcified embolism: A rare cause of cerebral infarction	Chandran, V.; Pai, A.; Rao, S.	2013	BMJ Case Reports.				No MAC
156	New xenograft valved conduit (Contegra) for right ventricular outflow tract reconstruction	Chatzis, A. C.; Giannopoulos, N. M.; Bobos, D.; Kirvassilis, G. B.; Rammos, S.; Sarris, G. E.	2003	Heart Surgery Forum	6(5)		396-398	No MAC
157	Identifying patients with degenerative mitral regurgitation for mitral valve repair and replacement: A transesophageal echocardiographic study	Chaudhry, F. A.; Upadya, S. P. Y.; Singh, V. P.; Cusik, D. A.; Izrailtyan, I.; Sanders, J.; Hargrove, C.	2004	Journal of the American Society of Echocardiogra phy	17(9)		988-994	No thrombus
158	Stroke due to calcific embolism after cardiac procedures	Chaudhry, F. S.; Vela-Duarte, D.; Biller, J.	2013	Arquivos de Neuro-Psiquiatria	71(6)		416	Embolism from procedure not MAC thrombus
159	Reconstructive surgery in congenital mitral valve insufficiency (Carpentier's techniques): long-term results	Chauvaud, S.; Fuzellier, J. F.; Houel, R.; Berrebi, A.; Mihaileanu, S.; Carpentier, A.	1998	Journal of Thoracic & Cardiovascular Surgery	115	1	84-92; discussion 92	No MAC, prosthetic valve
160	Serial cardiac magnetic resonance imaging of a rapidly progressing liquefaction necrosis of mitral annulus calcification associated with embolic stroke	Chen, O.; Dontineni, N.; Nahlawi, G.; Bhumireddy, G. P.; Han, S. Y.; Katri, Y.; Gulkarov, I. M.;	2012	Circulation	125	22	May-92	No thrombus

		Ciaburri, D. G.; Tortolani, A. J.; Lazzaro, R. S.; Sacchi, T. J.; Socolow, J. A.; Heitner, J. F.						
161	Mitral annular calcification versus mitral valve calcification [4]	Cheng, T. O.; Stein, J. H.; Soble, J. S.	1995	Stroke	26(12)		2374	No thrombus
162	Repeated operations on the heart in patients with a history of mitral commissurotomy. [Russian]	Chernov, V. A.	1984	Kardiologiia	24(7)		33-37	Mitral valve intervention
163	Spontaneous fistulization of a caseous calcification of the mitral annulus: an exceptional cause of stroke	Chevalier, B.; Reant, P.; Laffite, S.; Barandon, L.	2011	European Journal of Cardio-Thoracic Surgery	39	6	e184-5	Not MAC thrombus
164	Giant left atrium in rheumatic heart disease: the classic signs of left atrial enlargement	Chick, J. F.; Sheehan, S. E.; Miller, J. D.; Bair, R. J.; Madan, R.	2013	Journal of Emergency Medicine	44	6	e393-4	Rheumatic heart disease
165	Calcification of the mitral annulus fibrosus with systemic embolization: A clinicopathologic study of 16 cases	Ching-Shen, Lin; Schwartz, I. S.; Chapman, I.	1987	Archives of Pathology and Laboratory Medicine	111(5)		411-414	No thrombus on MAC
166	Computerized tomography in intracardiac tumors	Cholankeril, J. V.; Millman, A. E.; Ramamurti, S.; Ketyer, S.; Federici, E. E.	1983	Computerized Radiology	7	5	311-8	Not MAC
167	Open mitral commissurotomy in the current era: indications, technique, and results	Choudhary, S. K.; Dhareshwar, J.; Govil, A.; Airan, B.; Kumar, A. S.	2003	Annals of Thoracic Surgery	75	1	Jun-41	Rheumatic valve disease
168	False detection of left atrial thrombus by the angiographic sign of "neovascularity"	Chow, W. H.; Tai, Y. T.; Cheung, K. L.	1989	Catheterization & Cardiovascular Diagnosis	18	3	165-7	Not thrombus, not MAC

169	Mitral Valve Replacement Using Carpentier-Edwards Pericardial Bioprosthesis in Patients With Rheumatic Heart Disease Aged Below 40 Years: 17-Year Results	Chowdhury, U. K.; Rizvi, A.; Narang, R.; Seth, S.; Kalaivani, M.; Hasija, S.; Kumari, L.	2018	Heart, Lung & Circulation	27	7	864-871	Rheumatic heart disease
170	Twenty years of clinical experience with cardiac myxomas: Diagnosis, treatment, and follow up	Cianciulli, T. F.; Cozzarin, A.; Soumoulou, J. B.; Saccheri, M. C.; Mendez, R. J.; Beck, M. A.; Gagliardi, J. A.; Lax, J. A.	2019	Journal of Cardiovascular Imaging	27(1)		37-47	No MAC
171	Calcification of the left atrium. Case report (Rumanian). [Romanian]	Cimpeanu, Al; Pompilian, P.; Gutiu, I.; Carp, C.	1975	Medicina Interna	27(2)		149-154	Not MAC thrombus
172	Presidential address: Value, viability, and valves	Clarke, D. R.	2002	Journal of Thoracic and Cardiovascular Surgery	124(1)		06-Jan	Not MAC thrombus
173	Transthoracic echocardiography in stroke performed by neurologist: Pilot study. [Spanish]	Cocho, D.; Bravo, Y.; Leta, R.; Marti-Fabregas, J.; Carreras, F.; Pons, G.; Pujadas, S.; Aleu, A.; Marti-Vilalta, J. L.	2007	Neurologia	22(7)		420-425	Potential MAC thrombus
174	Long-term follow-up of the Hancock bioprosthetic heart valve: a 6-year review	Cohen, L. H.; Koster, J. K.; Mee, R. B.; Collins, J. J., Jr.	1979	European Heart Journal Cardiovascular Imaging	22(6)		E24-E57	Prosthetic valve
175	Comparison of late (62 to 140 months) degenerative changes in simultaneously implanted and explanted porcine (Hancock) bioprostheses in the tricuspid and mitral valve positions in six patients	Cohen, S. R.; Silver, M. A.; McIntosh, C. L.; Roberts, W. C.	1984	Circulation	60	2 Pt 2	87-92	Prosthetic valve

176	EACVI recommendations on cardiovascular imaging for the detection of embolic sources: Endorsed by the Canadian Society of Echocardiography	Cohen, A.; Donal, E.; Delgado, V.; Pepi, M.; Tsang, T.; Gerber, B.; Soulat-Dufour, L.; Habib, G.; Lancellotti, P.; Evangelista, A.; Cujec, B.; Fine, N.; Andrade, M. J.; Sprynger, M.; Dweck, M.; Edvardsen, T.; Popescu, B. A.; Bertrand, P.; Galderisi, M.; Haugaa, K. H.; Sade, L. E.; Stankovic, I.; Cosyns, B.	2021	American Journal of Cardiology	53	11	1599-602	No thrombus on MAC
177	Long-term follow-up of the Hancock bioprosthetic heart valve. A 6-year review	Cohn, L. H.; Koster, J. K.; Mee, R. B. B.; Collins Jr, J. J.	1979	Circulation	60(2 II)		I87-I92	Prosthetic valve
178	Early degeneration of a bioprosthetic mitral valve complicated by a large left atrial thrombus	Connors, C. W.; Christie, A. A.; Weldner, P. W.	2011	Seminars in Cardiothoracic & Vascular Anesthesia	15	3	112-4	Prosthetic valve
179	Technical problems in mitral valve repair and replacement	Cooley, D. A.	1989	Annals of Thoracic Surgery	48(4 SUPPL.)		S91-S92	No MAC thrombus
180	Mitral valvular stenosis and left atrium wall calcifications with left atrium massive thrombosis simulating atrial myxoma. [Italian]	Corea, L.; Alicandri, C.; Todisco, T.	1976	Cardiologia Pratica	27(1)		63-68	Publication before 1985
181	Bulky cardiac mass in uncommon location	Correia, J.	2017	European Heart Journal Cardiovascular Imaging	18(Supplement 2)		ii108	No thrombus

182	Closed mitral commissurotomy through a midline sternotomy. A useful and potentially advantageous alternative to the left thoracotomy approach	Cosio-Pascal, M.; Ibarra-Perez, C.	1974	American Journal of Surgery	127	6	721-4	Not MAC thrombus
183	Aortic valve surgery of the 21st century: Sutureless AVR versus TAVI	Costache, V. S.; Moldovan, H.; Arsenescu, C.; Costache, A.	2018	Minerva Cardioangiolog ica	66(2)		191-197	Not MAC thrombus
184	Systemic embolism in mitral valve disease	Coulshed, N.; Epstein, E. J.; McKendrick, C. S.; Galloway, R. W.; Walker, E.	1970	British Heart Journal	32	1	26-34	Rheumatic valve
185	CarboMedics Prosthetic Heart Valve	Craver, J.	1999	European Journal of Cardio-Thoracic Surgery	15 Suppl 1		S3-11; discussion S39	Prosthetic valves
186	Challenges in risk stratification of symptomatic and asymptomatic valvular heart disease: Lessons from large observational studies	Crea, F.	2020	European Heart Journal	41(45)		4289-4292	Not MAC thrombus
187	Early Bioprosthetic Valve Failure: Mechanistic Insights via Correlation between Echocardiographic and Operative Findings	Cremer, P. C.; Rodriguez, L. L.; Griffin, B. P.; Tan, C. D.; Rodriguez, E. R.; Johnston, D. R.; Pettersson, G. B.; Menon, V.	2015	Journal of the American Society of Echocardiogra phy	28	10	1131-48	Prosthetic valve
188	Adult aortic stenosis: Value of catheterisation for the study of associated lesions: A series of 137 cases. [French]	Crochet, D.; Petitier, H.; De Laguerenne, J.	1983	Archives des Maladies du Coeur et des Vaisseaux	76(9)		1057-1065	Not MAC thrombus
189	Neurologic complications of nonrheumatic valvular heart disease	Cruz-Flores, S.	2021	Handbook of Clinical Neurology	177		33-41	Not MAC thrombus
190	A case of iatrogenic severe mitral regurgitation	D'Aloia, A.; Piovanelli, B.; Rovetta, R.;	2014	Monaldi Archives for Chest Disease	80(3)		133-136	Not MAC thrombus

		Bonadei, I.; Vizzardi, E.; Curnis, A.; Metra, M.		- Cardiac Series				
191	Heart valve scaffold fabrication: Bioinspired control of macro-scale morphology, mechanics and micro-structure	D'Amore, A.; Luketich, S. K.; Raffa, G. M.; Olia, S.; Menallo, G.; Mazzola, A.; D'Accardi, F.; Grunberg, T.; Gu, X.; Pilato, M.; Kameneva, M. V.; Badhwar, V.; Wagner, W. R.	2018	Biomaterials	150		25-37	Not MAC thrombus
192	Unusual echocardiographic appearances attributable to submitral calcification simulating left ventricular "masses"	D'Cruz, I. A.; Devaraj, N.; Hirsch, L. J.; Glick, G.	1980	American Heart Journal	103(2)		295-298	Not MAC thrombus
193	Two-dimensional echocardiographic detection of staphyloccal vegetation attached to calcified mitral anulus	D'Cruz, I. A.; Collison, H. K.; Gerrardo, L.; Hensel, P.	1982	Clinical Cardiology	3	4	260-4	Not MAC thrombus
194	Multiphase TAVR CT identifies unexpected sticky situation (Mechanical mitral valve leaflet dysfunction and bicuspid aortic valve)	Dahiya, A.; Coucher, J.; Pratap, J.; Cole, C.	2021	Journal of Cardiovascular Computed Tomography	15(3)		e22-e24	Prosthetic valve
195	Single centre experience with transapical transcatheter mitral valve implantation	Dahle, G.; Rein, K. A.; Fiane, A. E.	2017	Interactive Cardiovascular & Thoracic Surgery	25	2	177-184	Valve intervention
196	Early Resolution of Heyde's Syndrome following Transcatheter Aortic Valve Replacement	Dall'Ara, G.; Grotti, S.; Conficoni, E.; Poletti, G.; Valpiani, D.; Carletti, R.; Compagnone, M.; Tarantino, F.; Galvani, M.	2021	Seminars in Thrombosis and Hemostasis	47(1)		102-104	Not MAC thrombus

197	The diagnosis of prosthesis pathology in biological and mechanical valves. I. Clinical and echocardiographic evaluation. [Italian]	Dander, B.; Tovena, D.; Buonanno, C.; Rossi, R.; Righetti, B.; Marino, P.; Zardini, P.	1985	Giornale italiano di cardiologia	15(5)		478-484	Prosthetic valves
198	Cardiological diagnosis in patients with arterial embolism. [German]	Daniel, W. G.; Durst, U. N.	1991	Herz	16(6)		405-418	Not MAC thrombus
199	Offering balloon mitral valvotomy to high wilkins score patients: Are we crossing the boundary? IPGMER Kolkata experience	Dasbiswas, A.; Ghosh Dastidar, D.; Maitra, A.	2010	EuroInterventio n. Conference: EuroPCR	6	SUPP L. H		RHD
200	TCTAP C-223 Para Valvular Leak Closure - Unconventional Way	Datta, G.	2019	Journal of the American College of Cardiology	73(15 Supplement)		S282-S283	Prosthetic valve
201	The risk for systemic embolization associated with percutaneous balloon valvuloplasty in adults. A prospective comprehensive evaluation	Davidson, C. J.; Skelton, T. N.; Kisslo, K. B.; Kong, Y.; Peter, R. H.; Simonton, C. A.; Phillips, H. R.; Behar, V. S.; Bashore, T. M.	1988	Annals of Internal Medicine	108	4	557-60	Valve intervention
202	Plasma NT-proBNP is a potential marker of disease severity and correlates with symptoms in patients with chronic rheumatic valve disease	Davutoglu, V.; Celik, A.; Aksoy, M.; Sezen, Y.; Soydinc, S.; Gunay, N.	2005	European Journal of Heart Failure	7	4	532-6	Rheumatic valve
203	Mitral-annulus calcification and cerebral or retinal ischaemia	de Bono, D. P.; Warlow, C. P.	1979	Lancet	2	8139	383-5	Not MAC thrombus
204	Multiple mycotic aneurysms of the aortic root after aortic valve replacement	De Gaspari, M.; Basso, C.; Perazzolo Marra, M.; Thiene, G.; Rizzo, S.	2020	Cardiovascular Pathology	44 (no pagination)			Not MAC thrombus
205	[Indications for the surgical repair in mitral insufficiency]	Degeorges, M.	1969	Archives des maladies du	100	SPEC. ISS. 1	19-28	Not MAC thrombus

				coeur et des vaisseaux				
206	The Best of valvular heart disease in 2006	De Gevigney, G.	2007	European Journal of Cardiovascular Medicine	3(1)		436-437	Not relevant, not a study.
207	A mitral annulus challenging mass: Role of cardiac magnetic resonance in the diagnosis of caseous calcification of the mitral annulus	De Lazzari, M.; Cecchetto, A.; De Conti, G.; Iliceto, S.; Marra, M. P.	2015	American Journal of Cardiovascular Disease	11(4)		494-498	Not MAC thrombus
208	Cerebral embolization from caseous mitral annular calcification	Del Castillo, M.; Burkland, D.; Letsou, G.; Stainback, R.	2017	Presse Medicale	77	5	151-2	Not MAC thrombus
209	Left atrial myxoma and transient ischemic attack: Case report	de Lima, M. F. M.; Varano, N.; Neto, O. P. A.; Mendes, D. S.	2021	Journal of the American College of Cardiology	69(11 Supplement 1)		2180	Not MAC thrombus
210	The Incidence and Clinical Course of Caseous Calcification of the Mitral Annulus: A Prospective Echocardiographic Study	Deluca, G.; Correale, M.; Ieva, R.; Salvatore, B. D.; Gramenzi, S.; Di Biase, M.	2008	Journal of the American Society of Echocardiography	21(7)		828-833	Not MAC thrombus
211	[Affection of cardiovascular system in antiphospholipid syndrome with non-bacterial thrombotic endocarditis]	Demin, A. A.; Chapaeva, N. N.; Trifonova, M. A.	2008	Terapevticheskii Arkhiv	80	8	Mar-51	Not MAC thrombus
212	Affection of cardiovascular system in antiphospholipid syndrome with non-bacterial thrombotic endocarditis. [Russian]	Demin, A. A.; Chapaeva, N. N.; Trifonova, M. A.	2008	Terapevticheskii Arkhiv	80(8)		51-53	Not MAC thrombus
213	Pseudoinfectious endocarditis in antiphospholipid syndrome. [Russian]	Demin, A. A.; Chapaeva, N. N.; Trifonova, M. A.	2008	Klinicheskaia meditsina	86(7)		14-18	Not MAC thrombus
214	Transcatheter mitral valve replacement in severe mitral	Demir, O. M.; Ruparelia, N.; Gopalan, D.; Busi, G.; Frame, A.;	2019	Cardiovascular Revascularization Medicine	20(3)		194-196	No MAC thrombus

	annular calcification and atrial septal defect closure	Sutaria, N.; Ariff, B.; Sen, S.; Malik, I.; Colombo, A.; Mikhail, G. W.						
215	Multimodality imaging in the diagnosis of caseous calcification of mitral annulus. [Turkish, English]	Demirkol, S.; Balta, S.; Bozlar, U.; Unlu, M.; Iyisoy, A.; Arslan, Z.; Kara, K.; Yokusoglu, M.; Kucuk, U.	2012	Turk Kardiyoloji Dernegi Arsivi	2)		151	Not MAC thrombus
216	A serious complication of percutaneous mitral valvuloplasty: systemic embolism. How can we decrease it? Case history	Demirtas, M.; Usal, A.; Birand, A.; San, M.; Batyraliev, T.; Niyazova, Z.	1996	Angiology	47	3	285-9	Valve intervention
217	Calcification of the mitral valve complicated by embolization and subsequent ischemic stroke with haemorrhagic transformation	Denegri, A.; Pedrazzini, G. B.; Moccetti, T.; Moccetti, M.	2015	European Heart Journal	36	40	2728	No thrombus on MAC
218	Left atrium 'Egg shell calcification'	Deora, S.; Patel, T.	2014	Heart Asia	4(1)		108-109	Rheumatic valve
219	Cardiac MRI for the detection of proximal sources of embolism in stroke and TIA patients	Desai, J. A.; Dobson, J. L.; Salahudeen, S.; Flood, J.; Nolan, R. L.; Jin, A. Y.	2011	Indian Journal of Pathology and Microbiology	58(2)		217-219	Not MAC thrombus
220	Cardiac MRI findings in the etiological workup of stroke and TIA	Desai, J. A.; Flood, J. R.; Dobson, J.; Salahudeen, S. R.; Nolan, R. L.; Jin, A. Y.	2011	Canadian Journal of Neurological Sciences	1)		S75	Not MAC thrombus
221	Idiopathic mitral valve prolapse with tricuspid, aortic and pulmonary valve involvement: An autopsy case report	Desai, H. M.; Amonkar, G. P.	2015	Stroke	42(11)		e594	No MAC
222	Structural valve deterioration 4 years after transcatheter aortic	Deutsch, M. A.; Mayr, N. P.; Assmann, G.; Will,	2015	Circulation	131(7)		682-685	Not relevant, TAVR.

	valve replacement imaging and pathohistological findings	A.; Krane, M.; Piazza, N.; Bleiziffer, S.; Lange, R.						
223	Calcification of a porcine valve xenograft during pregnancy--a case report and review of the literature	Deviri, E.; Yechezkel, M.; Levinsky, L.; Vidne, B. A.; Levy, M. J.	1984	Thoracic & Cardiovascular Surgeon	32	4	266-8	Mitral valve prosthesis
224	Quantification of regional platelet and calcium deposition on pericardial tissue valve prostheses in calves and effect of hydroxyethylene diphosphonate	Dewanjee, M. K.; Solis, E.; Mackey, S. T.; Lenker, J.; Edwards, W. D.; Didisheim, P.; Chesebro, J. H.; Zollman, P. E.; Kaye, M. P.	1986	Journal of Thoracic and Cardiovascular Surgery	92(3 I)		337-348	Animal study
225	New-onset acute rapidly deteriorating case of calciphylaxis after open heart surgery: A case report	Diasty, M. E.; Cuenca, J.	2021	European Heart Journal - Case Reports	5(3) (no pagination)			No MAC thrombus
226	Are patients with caseous calcification of the mitral annulus but without atrial fibrillation at increased risk of cerebral embolization?	Dietl, C. A.; Hawthorn, C. M.; Raizada, V.	2014	Circulation. Conference: American Heart Association's	130	SUPP L. 2		Not MAC thrombus
227	Risk of Cerebral Embolization with Caseous Calcification of the Mitral Annulus: Review Article	Dietl, C. A.; Hawthorn, C. M.; Raizada, V.	2016	The Open Cardiovascular Medicine Journal	10		221-232	Not MAC thrombus
228	Long-term results of bioprosthetic mitral valve replacement the pericardial perspective	Doenst, T.; Borger, M. A.; David, T. E.	2004	Journal of Cardiovascular Surgery	45(5)		449-454	Prosthetic valves
229	Mitral Arcades Unexpectedly Encountered During Cardiac Surgery	Dokollari, A.; Cameli, M.; Bisleri, G.; Pervez, M. B.; Kalra, D. K.; Demosthenous, M.; Yanagawa, B.; Pernoci, M.;	2021	Journal of Cardiothoracic and Vascular Anesthesia	35(3)		914-916	Not MAC

		Verma, S.; Latter, D.; Bonacchi, M.						
230	[Submitral left ventricular aneurysm: a rare disease in subsaharian African countries]	Donegani, E.; Casula, M.; Della Rocca, F.	2011	Giornale Italiano di Cardiologia	12	5	392-3	Not MAC
231	Caseous calcification of mitral annulus: A rare variant of mitral annular calcification	Dotan, M.; Isilak, Z.; Atalay, M.; Uz, O.	2014	American Journal of Cardiology	1)		S129-S130	Not MAC thrombus
232	Systemic embolism as a complication of percutaneous mitral valvuloplasty	Drobinski, G.; Montalescot, G.; Evans, J.; Nivet, M.; Thomas, D.; Grosgogeat, Y.	1992	Catheterization & Cardiovascular Diagnosis	25	4	327-30	Valve intervention
233	Transcatheter aortic valves produce unphysiological flows which may contribute to thromboembolic events: An in-vitro study	Ducci, A.; Pirisi, F.; Tzamtzis, S.; Burriesci, G.	2016	Journal of Biomechanics	49(16)		4080-4089	In vitro
234	Myocardial infarction as the first probable manifestation of caseous calcification of the mitral annulus	Durao, D.; Pitta Mda, L.; Alves, M.; Cabanelas, N.; Peres, M.; Aranha, J.; Monteiro, I.; Martins, V. P.; Francisco, A.; Leal, M.; Loureiro, J.; da Silva, G. F.	2009	Revista Portuguesa de Cardiologia	28	11	1271-5	Not MAC thrombus
235	Cardiogenic brain embolism	Dyken, M. L.; Fisher, M.; Harrison, M. J. G.	1986	Archives of Neurology	43(1)		71-84	Not MAC thrombus
236	The mitral valve replacement by the new-type bioprostheses (features of design and long-term results)	Dzemeshkevich, S. L.; Konstantinov, B. A.; Gromova, G. V.; Lyudinovskova, R. A.; Kudrina, L. L.	1994	Journal of Cardiovascular Surgery	35	6 Suppl 1	189-91	Prosthetic valve

237	The effects of the lampoon procedure on flow in the mitral valve anterior leaflet neo-sinus	Easley, T. F.; Sadri, V.; Dorbala, P.; Kamioka, N.; Babaliaros, V.; Yoganathan, A. P.	2019	Circulation. Conference: American Heart Association Scientific Sessions, AHA	140	Supplement 1		In vitro
238	Two cases of warfarin-induced tracheobronchial calcification after Fontan surgery	Eckersley, L.; Stirling, J.; Occleshaw, C.; Wilson, N.	2014	Pediatric Cardiology	35(6)		954-958	Not MAC thrombus
239	Pathology of acquired valvular disease of the heart	Edwards, J. E.	1979	Seminars in Roentgenology	14	2	96-115	Not MAC
240	Outcome of anticoagulation therapy for bioprosthetic valve thrombosis: A prospective study	Egbe, A. C.; Connolly, H.; Pellikka, P.; Nkomo, V.; Pislaru, S.	2017	Journal of the American College of Cardiology	69(11 Supplement 1)		1948	Prosthetic valve
241	Mitral and aortic valve decalcification by ultrasonic energy. Experimental report	Eguaras, M. G.; Saceda, J. L.; Luque, I.; Concha, M.	1988	Journal of Thoracic & Cardiovascular Surgery	100	2	161-6	Not MAC thrombus
242	A comparison of repair and replacement for mitral stenosis with partially calcified valve	Eguaras, M. G.; Luque, I.; Montero, A.; Garcia, M. A.; Calleja, F.; Roman, M.; Concha, M.; Ocerin, J. M.	1990	Journal of Thoracic & Cardiovascular Surgery	95	6	1038-40	Valve intervention
243	The mosaic bioprosthesis in the aortic position: hemodynamic performance after 2 years	Eichinger, W. B.; Schutz, A.; Simmerl, D.; Gansera, B. U.; Breuer, M.; Haslinger, B.; Kemkes, B. M.	1998	Annals of Thoracic Surgery	66	6 Suppl	S126-9	Prosthetic valve
244	Mobile mitral annular mass	Elalmis, O. U.; Cicekciotlu, H.; Ulusoy, F. V.	2012	Annals of Thoracic Surgery	52	1	Sep-66	Not MAC thrombus

245	Mitral valve repair in the extensively calcified mitral valve annulus	el Asmar, B.; Acker, M.; Couetil, J. P.; Perier, P.; Dervanian, P.; Chauvaud, S.; Carpentier, A.	1991	Journal of Heart Valve Disease	9	1	75-80; discussion 81	Valve intervention
246	Percutaneous Transvenous Transseptal Transcatheter Valve Implantation in Failed Bioprosthetic Mitral Valves, Ring Annuloplasty, and Severe Mitral Annular Calcification	Eleid, M. F.; Cabalka, A. K.; Williams, M. R.; Whisenant, B. K.; Alli, O. O.; Fam, N.; Pollak, P. M.; Barrow, F.; Malouf, J. F.; Nishimura, R. A.; Joyce, L. D.; Dearani, J. A.; Rihal, C. S.	2016	International Journal of Cardiology	1)		S94	Not MAC thrombus
247	Surgical repair of the prolapsing anterior leaflet in degenerative mitral valve disease	El Khoury, G.; Noirhomme, P.; Verhelst, R.; Rubay, J.; Dion, R.	2000	JACC: Cardiovascular Interventions	9(11)		1161-1174	Valve intervention
248	[the Prognosis of Operated Mitral Stenosis]	Ellis, L. B.; Harken, D. E.; Cainee, R.; Acar, J.; Joly, F.; Carlotti, J.; Belcher, J. R.; Gupta, R. L.	1964	Presse Medicale	72		Aug-67	Previous mitral valve intervention
249	Fifteen-to twenty-year study of one thousand patients undergoing closed mitral valvuloplasty	Ellis, L. B.; Singh, J. B.; Morales, D. D.; Harken, D. E.	1973	Circulation	48	2	357-64	Mitral valve intervention
250	Strategies and Devices to Minimize Stroke in Adult Cardiac Surgery	Engelman, R. M.; Engelman, D. T.	2015	Seminars in Thoracic and Cardiovascular Surgery	27(1)		24-29	Not MAC thrombus
251	Key role of Doppler echocardiography in the emergency management of elderly patients	Ennezat, P. V.; Logeart, D.; Berrebi, A.;	2010	Acta Cardiologica	76(2)		216-217	Not MAC thrombus

		Vincentelli, A.; Marechaux, S.						
252	Fenfluramine induced mitral stenosis complicated by massive left atrial thrombosis	Ennezat, P. V.; Arnaud-Crozat, E.; Guerbaai, R. A.; Stephanov, O.	2021	Archives of Cardiovascular Diseases	103(2)		115-128	No MAC (mildly calcified leaflets)
253	Implication of eruption of caseous calcification of mitral annulus	Enomoto, Y.; Sudo, Y.; Hasegawa, H.	2019	Asian Cardiovascular & Thoracic Annals	27	6	495-497	Not MAC thrombus
254	Important cardiac findings on routine CT examinations: A practical approach and review	Entrikin, D.; Schoppe, K.; Romesburg, J.; Carr, J.	2012	American Journal of Roentgenology. Conference	198	5 SUPPL. 1		Not MAC thrombus
255	Caseous calcification of the mitral annulus, a rare but possible source of cardioembolic stroke	Equiza, J.; De La Riva, P.; De Arce, A.; Gonzalez, F.; Diez Gonzalez, N.; Villanueva, I.; Rodriguez-Antiguedad, J.; Campo-Caballero, D.; Iruzubieta Agudo, P.; Martinez-Zabaleta, M.	2020	International Journal of Stroke	15(1 SUPPL)		715	Not MAC thrombus
256	2014 ESC guidelines on the diagnosis and treatment of aortic diseases	Erbel, R.; Aboyans, V.; Boileau, C.; Bossone, E.; Di Bartolomeo, R.; Eggebrecht, H.; Evangelista, A.; Falk, V.; Frank, H.; Gaemperli, O.; Grabenwoger, M.; Haverich, A.; Iung, B.; Manolis, A. J.; Meijboom, F.; Nienaber, C. A.; Roffi, M.; Rousseau, H.; Sechtem, U.;	2014	European Heart Journal	35(41)		2873-2926	Not MAC thrombus

		Sirnes, P. A.; Von Allmen, R. S.; Vrints, C. J. M.; Zamorano, J. L.; Achenbach, S.; Baumgartner, H.; Bax, J. J.; Bueno, H.; Dean, V.; Deaton, C.; Erol, C.; Fagard, R.; Ferrari, R.; Hasdai, D.; Hoes, A.; Kirchhof, P.; Knuuti, J.; Kolh, P.; Lancellotti, P.; Linhart, A.; Nihoyannopoulos, P.; Piepoli, M. F.; Ponikowski, P.; Tamargo, J. L.; Tendera, M.; Torbicki, A.; Wijns, W.; Windecker, S.; Czerny, M.; Deanfield, J.; Di Mario, C.; Pepi, M.; Taboada, M. J. S.; Van Sambeek, M. R.; Vlachopoulos, C.						
257	The role of echocardiography in the management of the sources of embolism	Esposito, R.; Raia, R.; De Palma, D.; Santoro, C.; Galderisi, M.	2012	Future Cardiology	8	1	101-14	Not MAC thrombus
258	Predictive value of free fatty acid levels in embolic stroke of undetermined source: A retrospective observational study	Eun, M. Y.; Sung, J. H.; Lee, S. H.; Jung, I.; Park, M. H.; Kim, Y. H.; Jung, J. M.	2020	Medicine	99	40	e22465	No MAC thrombus
259	Fresh autologous pericardium for leaflet perforation repair in mitral valve infective endocarditis	Evans, C. F.; DeFilippi, C. R.; Shang, E.; Griffith,	2013	Journal of Heart Valve Disease	22	4	560-6	Mitral valve intervention

		B. P.; Gammie, J. S.					
260	Tiny bubbles: Coronary aneurysms and lupus	Famularo, G.; Vallone, A.; Compagnucci, M.; Capo, G.; Minisola, G.; Gasbarrone, L.	2014	American Journal of Medicine	127(10)	933-935	Not MAC thrombus
261	Mitral annulus calcification: clinical observation on 72 patients. [Chinese]	Fang, B. R.; Chiang, C. W.; Lee, Y. S.	1991	Journal of the Formosan Medical Association = Taiwan yi zhi	90(3)	304-307	Not MAC thrombus
262	Left main perforation and acute stent thrombosis-a cath lab nightmare	Fang, H. Y.; Wu, C. J.	2011	Catheterization and Cardiovascular Interventions	78(2)	237-243	Not MAC thrombus
263	Pulmonary hypertension: Prevalence and risk factors	Faqih, S. A.; Noto-Kadou-Kaza, B.; Abouamrane, L. M.; Mtiou, N.; El Khayat, S.; Zamd, M.; Medkouri, G.; Benghanem, M. G.; Ramdani, B.	2016	IJC Heart and Vasculature	11	87-89	No MAC
264	Mitral anullar mass-when echocardiographic features do CMACcurate	Faria Da Mota, T.; Fernandes, R.; Sousa Bispo, J.; Azevedo, P.; Guedes, J. P.; Silva, D.; Amado, J.; Vieira, M.; Mendonca Cafe, H.; De Jesus, I.	2019	European Heart Journal Cardiovascular Imaging	20(Supplement 1)	i835	Not MAC thrombus
265	Rupture of chordae tendineae in patients with beta-thalassemia	Farmakis, D.; Deftereos, S.; Giakoumis, A.; Polymeropoulos, E.; Aessopos, A.	2004	European Journal of Haematology	72(4)	296-298	Not MAC

266	Predictors of successful percutaneous transvenous mitral commissurotomy using the bonhoeffer Multi-Track system in patients with moderate to severe mitral stenosis: Can we see beyond the Wilkins score?	Farman, M. T.; Khan, N.; Sial, J. A.; Saghir, T.; Ashraf, T.; Rasool, S. I.; Zaman, K. S.	2015	Anadolu Kardiyoloji Dergisi	15(5)		373-379	RHD, mitral valve intervention
267	Mitral repair in patients with severely calcified annulus: feasibility, surgery and results	Fasol, R.; Mahdjoobian, K.; Joubert-Hubner, E.	2002	Journal of Heart Valve Disease	11	2	153-9	Not MAC thrombus
268	Diagnosis and differential therapy of mitral stenosis. [German]	Fassbender, D.; Schmidt, H. K.; Seggewiss, H.; Mannebach, H.; Bogunovic, N.	1998	Herz	23(7)		420-428	Not MAC thrombus
269	Code stroke! a case of nonbacterial thrombotic endocarditis	Feghaly, J.; Ampadu, J.; Das, D.	2020	Journal of Investigative Medicine	68(5)		1057-1058	Not MAC
270	The long-term follow-up of 1000 patients with a valve replacement by the prosthesis of the Instituto Nacional de Cardiologia Ignacio Chavez. [Spanish]	Fernandez de la Reguera, G.; Barragan Garcia, R.; Alzaga, M. T.; Peon, J. M.; Cardenas, M.; Soni, J.; Kabela, E.	1992	Archivos del Instituto de Cardiologia de Mexico	62(6)		513-520	Prosthetic valves
271	Structural changes in implanted cardiac valvular bioprostheses constructed of glycerol-treated human dura mater	Ferrans, V. J.; Milei, J.; Ishihara, T.; Storino, R.	1991	European Journal of Cardio-Thoracic Surgery	5	3	144-54	Mitral valve prosthesis
272	Results of reoperation for failure of cardiac bioprostheses	Festa, M.; Renzulli, A.; Sante, P.; Micheletti, E.; Giannolo, B.; Palma, G.; Cotrufo, M.	1987	Italian Journal of Surgical Sciences	17	3	213-7	Prosthetic valves

273	Prevalence and consequences of noncardiac incidental findings on preprocedural imaging in the workup for transcatheter aortic valve implantation, renal sympathetic denervation, or MitraClip implantation	Feyz, L.; El Faquir, N.; Lemmert, M. E.; Misier, K. R.; van Zandvoort, L. J. C.; Budde, R. P. J.; Boersma, E.; Zijlstra, F.; de Jaegere, P.; Van Mieghem, N. M.; Daemen, J.	2018	American Heart Journal	204		83-91	Not MAC thrombus
274	Valvular disease in patients requiring long-term left ventricular assist devices: Pathophysiology and therapeutic options	Firstenberg, M. S.; Sai-Sudhakar, C. B.; Anyanwu, A.	2012	Expert Review of Cardiovascular Therapy	10(2)		205-213	Not MAC thrombus
275	Transcatheter aortic valve implantation: Status and challenges	Fishbein, G. A.; Schoen, F. J.; Fishbein, M. C.	2014	Cardiovascular Pathology	23(2)		65-70	Not MAC
276	Vitamin K and vascular calcifications	Fodor, D.; Albu, A.; Poanta, L.; Porojan, M.	2010	Acta Physiologica Hungarica	97(3)		256-266	Not MAC
277	Incidental cardiac findings on computed tomography imaging of the thorax	Foley, P. W.; Hamaad, A.; El-Gendi, H.; Leyva, F.	2010	BMC Research Notes	3		326	No MAC thrombus
278	Surgical Management of Caseous Calcification of the Mitral Annulus	Fong, L. S.; McLaughlin, A. J.; Okiwelu, N. L.; Nordstrand, I. A. J.; Newman, M.; Passage, J.; Joshi, P. V.	2017	Annals of Thoracic Surgery	104(3)		e291-e293	Not MAC thrombus
279	Echocardiographic changes associated with risk of developing embolic complications in patients with ischemic stroke. [Russian]	Foniakin, A. V.; Geraskina, L. A.; Suslina, Z. A.	2002	Terapevticheskii arkhiv	74(11)		71-74	No thrombus on MAC
280	An unusual coronary vein lesion thrombus with calcification	Ford, J. C.; Ascah, K. J.; Walley, V. M.	1998	Cardiovascular Pathology	7	3	169-72	Not MAC thrombus

281	Implications of a large atrial roof thrombus	Fountoulakis, P.; Hamodraka, E.; Siama, A.; Tsoukas, A.; Manolis, A. J.	2020	European Heart Journal Cardiovascular Imaging	21(Supplement 1)		i796	Rheumatic valve RHD
282	Indications for surgical replacement of the mitral valve. With particular reference to common and uncommon causes of mitral regurgitation	Fowler, N. O.; van der Bel-Kahn, J. M.	1979	American Journal of Cardiology	44	1	148-57	No MAC thrombus
283	The Mitral/Aortic Flow Velocity Integral Ratio in Mitral Regurgitation	Fox, J. M.; Trzcinka, A.; Cobey, F.	2020	Journal of Cardiothoracic and Vascular Anesthesia	34(1)		289-293	No MAC thrombus
284	Caseous calcification of the mitral annulus: case report	Franca, L. A.; Rodrigues, A. C.; Vieira, M. L.; Oliveira, W. A.; Azevedo, R. E.; Cordovil, A.; Lira-Filho, E. B.; Fischer, C. H.; Morhy, S. S.	2013	Einstein	11	3	370-2	Not MAC thrombus
285	Quattro valve trial at mid-term: December 1996 to November 2004	Frater, R. W.; Sussman, M.; Middlemost, S.; Walther, T.; Mohr, F.; Al Halees, Z.; Shahid, M.	2006	Journal of Heart Valve Disease	15	2	230-7; discussion 237	Mitral valve prosthesis
286	Big mitral annular calcification: a case report of a dynamic liquefaction necrosis as a potential source of embolism	Frey, S. M.; Hofmann, V.; Zellweger, M. J.; Haaf, P.	2021	European Heart Journal. Case Reports	5	10	ytab380	Not MAC thrombus
287	[Valve replacement in children: results and long-term follow-up in 171 operated patients]	Friedli, B.; Friedli, G. M.; Ben Ismail, M.; Rouge, J. C.; Hahn, C.; Faidutti, B.	1981	Schweizerische Medizinische Wochenschrift. Journal Suisse de Medecine	111	27-28	1044-8	Mitral valve prosthesis
288	Intracardiac echocardiography via the transvenous approach with	Fu, M.; Hung, J. S.; Lo, P. U.; Wu,	1999	Mayo Clinic Proceedings	74(8)		775-783	No MAC thrombus

	use of 8F 10-MHz ultrasound catheters	C. J.; Chang, K. C.; Lau, K. W.						
289	A case of mitral valve replacement with a collar-reinforced prosthetic valve for heavily calcified mitral annulus	Fukada, Y.; Matsui, Y.; Sasaki, S.; Yasuda, K.	2005	Annals of Thoracic & Cardiovascular Surgery	11	4	260-3	Prosthetic valve
290	Transesophageal echocardiography discloses unexpected cardiac sources of embolus in stroke patients aged more than 45 years	Fukujima, M. M.; Tatani, S. B.; Aguiar, A. S.; De Rezende Ferraz, M. E. M.; Francisco, S.; Ferreira, L. D.; Monaco, C. G.; Ortiz, J.; Costa Lima, J. A.; Gabbai, A. A.; Do Prado, G. F.	2005	Arquivos de Neuro-Psiquiatria	63(4)		941-945	No MAC thrombus
291	Recurrent ischemic stroke as a result of caseous calcifications of the mitral annulus under dual anti-platelet therapy	Fukushima, N.; Yoshizawa, H.; Iijima, M.; Nagashima, Y.; Yamasaki, K.; Hagiwara, N.; Kitagawa, K.	2017	Neurology and Clinical Neuroscience	5(6)		181-182	Not MAC thrombus
292	Calcification of the mitral annulus: etiology, clinical associations, complications and therapy	Fulkerson, P. K.; Beaver, B. M.; Auseon, J. C.; Graber, H. L.	1979	American Journal of Medicine	66	6	967-77	Not MAC thrombus
293	[Value of MRI with injection of gadolinium in the diagnosis of mitral ring abscess. Apropos of a case]	Furber, A.; Geslin, P.; Le Jeune, J. J.; Doss-Louca, N.; Laporte, J.; Jallet, P.; Tadei, A.	1997	Archives des Maladies du Coeur et des Vaisseaux	90	3	399-404	Not MAC
294	Cerebrovascular complications associated with idiopathic hypertrophic subaortic stenosis	Furlan, A. J.; Craciun, A. R.; Raju, N. R.; Hart, N.	1984	Stroke	15(2)		282-284	No MAC thrombus
295	Risk of stroke in patients with mitral annulus calcification	Furlan, A. J.; Craciun, A. R.;	1984	Stroke	15	5	801-3	Not MAC thrombus

		Salcedo, E. E.; Mellino, M.						
296	[Heart valve bioprostheses of the "Bionix" series. 6-year experience with the mitral valve replacement]	Fursov, B. A.; Zaitsev, V. V.; Bykova, V. A.; Mishchenko, B. P.; Gorshkov Iu, V.; Svanidze, O. G.; Evdokimov, S. V.; ZaitsevLv,; Rusanov, N. I.; Tsukerman, G. I.	1991	Grudnaia i Serdechno-Sosudistaia Khirurgiia		10	06-Nov	Prosthetic valve
297	Bleeding, vertebral fractures and vascular calcifications in patients treated with warfarin: Hope for lower risks with alternative therapies	Fusaro, M.; Crepaldi, G.; Maggi, S.; D'Angelo, A.; Calo, L.; Miozzo, D.; Fornasieri, A.; Gallieni, M.	2011	Current Vascular Pharmacology	9(6)		763-769	No MAC
298	Editor-in-Chief's Top Picks From 2015: Part Two	Fuster, V.	2016	Journal of the American College of Cardiology	67(7)		817-842	Not MAC thrombus
299	A Malignant Imaging Course of a Benign Mass	Gajanan, G.; Asawaeer, M.; Velagapudi, P.; Sayyed, S.; Chatzizisis, Y. S.	2020	Journal of the American College of Cardiology	75(11)		2659	Not MAC thrombus
300	Curious case of calciphylaxis leading to acute mitral regurgitation	Gallimore, G. G.; Curtis, B.; Smith, A.; Benca, M.	2014	BMJ Case Reports	30		30	No MAC thrombus
301	Isolated mitral valve replacement with the Hancock bioprosthesis: A 13-year appraisal	Gallucci, V.; Bortolotti, U.; Milano, A.	1984	Annals of Thoracic Surgery	38(6)		571-578	Mitral valve prosthesis
302	Mitral valve replacement with porcine bioprosthesis in children. Evaluation of 29 patients during 12 years. [Portuguese]	Gandra, S. M.; Rivetti, L. A.; Pinto, A. M.; Marinelli, I.; Campagnucci, V. P.; Franken, R. A.	1992	Arquivos brasileiros de cardiologia	58(2)		101-105	Mitral valve prosthesis

303	Complicated rheumatic mitral stenosis presenting in an elderly patient and the challenges in its management	Ganeshpure, S.; Vaidya, G. N.; Gattani, V.	2012	BMJ Case Reports.				RHD
304	Caseous calcification of the mitral annulus mimicking benign cardiac tumour of the mitral valve	Gao, H.; Yao, L.; Cheng, Y.; Wu, C.; Mei, X.; Mou, Y.; Jiang, L.; Zheng, Z.	2021	Cardiovascular Journal of Africa	32	4	224-227	Not MAC thrombus
305	Echocardiographic criteria in selection of patients for percutaneous mitral commissurotomy	Garbarz, E.; Iung, B.; Cormier, B.; Vahanian, A.	1999	Echocardiogra phy	16(7 I)		711-721	RHD, valve intervention
306	Valvular heart disease in primary antiphospholipid syndrome (PAPS): Clinical and morphological findings	Garcia-Torres, R.; Amigo, M. C.; De La Rosa, A.; Moron, A.; Reyes, P. A.	1996	Lupus	5(1)		56-61	Not MAC thrombus
307	Calcified left ventricular aneurysm: "An egg in the heart"	Garg, P.; Rodrigues, E.	2010	JACC: Cardiovascular Imaging	6(1)		105-119	Not MAC thrombus
308	Echocardiography in liver transplant candidates	Garg, A.; Armstrong, W. F.	2013	Open Cardiovascular Medicine Journal	4		214-215	No MAC thrombus
309	Spontaneous calcific cerebral embolus	Gearry, R. B.; Sharr, J. P.; Avery, S. F.	2005	Australasian Radiology	49(2)		154-156	Not MAC thrombus
310	Cardiac masses: Experience from a Turkish tertiary center of cardiology	Gecmen, C.; Gecmen, G. G.; Kahyaoglu, M.; Omar, B.; Izci, S.; Kalayci, A.; Karabay, C. Y.; Coban, S.; Candan, O.; Yanik, E.; Izgi, I. A.; Barisik, N. O.	2017	Herz	42(7)		690-695	Not MAC thrombus

311	Unusual cardiac manifestation of Systemic lupus erythematosus	Gegenava, M.; Huizinga, T. W. J.	2019	European Heart Journal: Acute Cardiovascular Care	8(Supplement 1)		297	Not MAC thrombus
312	Cerebral embolism following mitral valvotomy	Geldof, W. C.; Roos, J. P.; Brom, A. G.	1971	Acta Cardiologica	26	4	392-9	Mitral valve intervention
313	Is there an association between arteriovenous fistula thrombosis and presence of the cardiac valve calcification in non-diabetic hemodialysis patients	Gelev, S.; Trajceska, L.; Srbinovska, E.; Pavleska, S.; Oncevski, A.; Dejanov, P.; Gerasomovska, V.; Selim, G.; Sikole, A.	2012	International Journal of Artificial Organs	38(7)		394	Not MAC thrombus
314	Association between cardiac valve calcification and arteriovenous fistula thrombosis in hemodialysis patients	Gelev, S.	2015	Nephrology Dialysis Transplantation	2)		ii258-ii259	Not MAC thrombus
315	Gross and histological findings in bioprosthetic mitral valves explanted from children under 5 years of age	Gellis, L.; Baird, C. W.; Emani, S.; Borisuk, M.; Gauvreau, K.; Paders, R. F.; Sanders, S. P.	2017	Journal of Thoracic & Cardiovascular Surgery	155	2	746-752	Mitral valve prosthesis
316	Morphologic and histologic findings in bioprosthetic valves explanted from the mitral position in children younger than 5 years of age	Gellis, L.; Baird, C. W.; Emani, S.; Borisuk, M.; Gauvreau, K.; Padera, R. F., Jr.; Sanders, S. P.	2018	Cardiology in the Young	27(4)		S548-S549	Mitral valve prosthesis
317	Large vessel occlusion stroke due to dislodged aortic valve calcification revealed by imaging and histopathology	Genchi, A.; Schwarz, G.; Semerano, A.; Callea, M.; Sanvito, F.; Simionato, F.; Panni, P.; Scomazzoni, F.; Doglioni, C.; Comi,	2020	Journal of the Neurological Sciences	408 (no pagination)			Not MAC thrombus

		G.; Falini, A.; Ancona, F.; Filippi, M.; Roveri, L.; Bacigaluppi, M.						
318	Prediction of recurrence after cryoballoon ablation therapy in patients with paroxysmal atrial fibrillation	Gerede, D. M.; Candemir, B.; Vurgun, V. K.; Aghdam, S. M.; Acibuca, A.; Ozcan, O. U.; Goksuluk, H.; Kervancioglu, C.; Erol, C.	2016	Anatolian Journal of Cardiology	16(7)		482-488	Not MAC thrombus
319	Two cases of Fahrvs syndrome with hypoparathyroidism	Gerenova, J. B.; Mateeva, V. M.; Prodanova, D. I.; Valkanova, G. P.; Mitev, M. A.; Obretenov, E. D.	2015	Endokrinologya	20(2)		106-108 and 109	Not MAC thrombus
320	Caseous calcification of the mitral annulus: a rare entity confirmed by cardiovascular magnetic resonance imaging	Ghazawi, F. M.; Vinco, G.; Walker, M.	2018	The International Journal of Cardiovascular Imaging	34	1	25-26	Not MAC thrombus
321	ECG-gated MDCT imaging after aortic and mitral valve corrective surgery: Correlation with echocardiography, fluoroscopy, and surgical findings	Ghersin, E.; Martinez, C.; Fishman, J.; Tracy, M.; Bokhari, S.; O'Neill, W.	2012	American Journal of Roentgenology. Conference	198	5 SUPPL. 1		Valve intervention
322	Multiple coronary fistula formation associated with a huge left atrial thrombus in rheumatic mitral valve stenosis	Gholoobi, A.; Poorzand, H.	2014	Journal of Cardiology Cases	9(3)		124-126	Rheumatic valve
323	Transcatheter Valve Implantation in Mitral Annular Calcification During Open Surgery: Extended Collar Technique	Ghosh-Dastidar, M.; Bapat, V.	2017	Annals of Thoracic Surgery	104(3)		e303-e305	Valve intervention

324	Left atrial mass produced by extensive mitral annular calcification	Giannoccaro, P. J.; Ascah, K. J.; Chan, K. L.; Walley, V. M.	1991	Journal of the American Society of Echocardiography	4	6	619-22	No thrombus on MAC
325	Left atrial appendage thrombus despite long-term treatment with dabigatran complicating aortic valve replacement for severe aortic stenosis	Gibbs, O.; Faour, A.; Hsu, D.; Jain, M.; Lo, S.	2017	Heart Lung and Circulation	26(Supplement 2)		S169	Not MAC thrombus
326	Training seminar "Echocardiography: Heart valve diseases - Surgery versus monitoring" - December 1, 2007, Wilhelminenberg Castle, Vienna. [German]	Glaser, F.	2008	Journal fur Kardiologie	15(3-4)		90-94	Not MAC thrombus
327	The role of percutaneous transvenous balloon mitral valvuloplasty in the treatment of patients with symptomatic mitral stenosis	Glazier, J. J.; Benit, E.; Vrolix, M. C.; Rocha, P.; De Geest, H.; Van de Werf, F.	1992	Acta Clinica Belgica	47	4	256-63	Valve intervention
328	Comparison between transthoracic and transesophageal echocardiography in the evaluation of candidates to percutaneous mitral valvuloplasty. [Italian]	Gobbi, M.; Ferlito, M.; Rapezzi, C.; Bacchi Reggiani, M. L.; Ortolani, P.; Piovaccari, G.; Zimarino, M.; Magnani, G.; Marzocchi, A.; Branzi, A.	1994	Cardiologia (Rome, Italy)	39(4)		261-268	RHD
329	Histotopographic evidence that amyloid deposits in sclerocalcific heart valves and other chronic lesions of the cardiovascular system are related to old thrombotic material	Goffin, Y. A.; Rickaert, F.	1986	Virchows Archiv - A Pathological Anatomy and Histopathology	409(1)		61-77	Not MAC thrombus
330	Mitral valve replacement with bileaflet preservation for complex annular calcification	Goksel, O. S.; Inan, K.; Tatar, T.; Ucak, A.; Arslan, G.; Us, M.; Yilmaz, A. T.	2008	Heart Surgery Forum	11	1	E1-3	No MAC thrombus

331	Caseous calcification of the mitral annulus causing multiple cardioembolic strokes	Goldberg, A.; Singh, G.; Boateng, S.; Tracy, M.	2016	Journal of the American College of Cardiology	1)		1184	Not MAC thrombus
332	Left atrial calcification (Serbocroatian). [Serbian]	Goldner, V.; Bubanj, D.; Asperger, Z.	1974	Lijecnicki Vjesnik	96(4)		227-230	Not MAC thrombus
333	Early clinical experience with the Tissuemed porcine bioprosthesis	Goldsmith, I.; Mukundan, S.; Nugent, A.; Rosin, M. D.	1998	Annals of Thoracic Surgery	66	6 Suppl	S259-63	Mitral valve prosthesis
334	Echocardiography: Guidance during valve implantation	Goncalves, A.; Marcos-Alberca, P.; Zamorano, J. L.	2010	EuroInterventio n	6(SUPP L. G)		G14-G19	Not MAC thrombus
335	Multiple extremity necrosis in fatal calciphylaxis: Case report	Gonzalez, D. E.; Foresto, R. D.; Santos Maldonado, A. L.; Carvalho Padilha, W. S.; Roberto, F. B.; da Costa Pereira, M. E. V.; de Souza Durao, M.; Carvalho, A. B.	2021	Jornal Brasileiro de Nefrologia	43(2)		274-278	No MAC thrombus
336	Calcified bicuspid aortic valve mass prolapsing into the left main coronary artery	Goraya, T. Y.; Mookadam, F.; Lapeyre, Iii A. C.	2000	Mayo Clinic Proceedings	75(10)		1081-1085	No MAC
337	Failure mode of a new pericardial valve prosthesis (Sorin Pericarbon). A morphological study	Grabenwoger, M.; Grimm, M.; Leukauf, C.; Szeles, C.; Feichtinger, E.; Muller, M. M.; Moritz, A.; Bock, P.; Wolner, E.	1994	European Journal of Cardio-Thoracic Surgery	8	9	470-6	Mitral valve prosthesis
338	Caseous calcification of the mitral annulus and pulmonary arteriovenous malformation in a	Granitz, C.; Motloch, L. J.; Hoppe, U. C.;	2019	European Heart Journal	20(Supplement 2)		ii310-ii311	Not MAC thrombus

	patient with embolic stroke diagnosed by one stop shop MRI	Hergan, K.; Granitz, M.		Cardiovascular Imaging				
339	Transventricular mitral valvulotomy. Analysis of factors influencing operative and late results	Grantham, R. N.; Daggett, W. M.; Cosimi, A. B.; Buckley, M. J.; Mundth, E. D.; McEnany, T.; Scannell, J. G.; Austen, W. G.	1974	Circulation	50	2 Suppl	II200-12	Mitral valve intervention
340	Duplex ultrasound mapping protocol for placement of cardiopulmonary bypass cannulae for robotic mitral valve surgery	Grattan, A. G.; Digiannantonio, A.; Mihaljevic, T.; Gillinov, A. M.; Gornik, H. L.	2011	Journal for Vascular Ultrasound	35(3)		143-147	Not MAC thrombus
341	Reoperation for mitral stenosis	Gray, A.; Kitchin, A. H.; Logan, A.; Turner, R. W.	1969	British Heart Journal	31	6	795	Previous mitral valve intervention
342	Premolded bovine pericardial chords for replacement of ruptured or elongated chordae tendineae	Gregori Jr, F.; Leal, J. C.; Braile, D. M.	2010	Heart Surgery Forum	13(1)		E17-E20	Not MAC
343	[Radiographic signs of chronic disorder of the pulmonary circulation in mitral diseases]	Grigorian, E. A.	1968	Kardiologiia	8	3	Apr-40	Not MAC thrombus
344	Dynamic nature of caseous mitral annular calcification	Groves, D. W.; Acharya, T.; Shanbhag, S. M.; Bandettini, W. P.; Arai, A. E.; Chen, M. Y.	2018	Journal of Cardiovascular Computed Tomography	12(5)		444-446	Not MAC thrombus
345	Mitral stenosis and percutaneous mitral valvuloplasty (part 2)	Guerios, E. E.; Bueno, R.; Nercolini, D.; Tarastchuk, J.; Andrade, P.; Pacheco, A.; Faidiga, A.; Negrao, S.; Barbosa, A.	2005	Journal of Invasive Cardiology	17(8)		440-444	Valve intervention

346	Transcatheter mitral valve replacement with balloon expandable valves in native mitral valve disease due to severe mitral annular calcification: Results from the first global registry	Guerrero, M.; Dvir, D.; Himbert, D.; Urena, M.; Mahadevan, V. S.; Eleid, M.; O'Hair, D.; Martinez-Clark, P.; Witkowski, A.; Wendler, O.; Rodes-Cabau, J.; Dumonteil, N.; Ferrari, E.; Ciaburri, D.; Suh, W. M.; Vorobiof, G.; Greenbaum, A.; Wang, D. D.; Paone, G.; Palma, J. H.; Dager, A. E.; Linke, A.; Kornowski, R.; Nickenig, G.; Cribier, A. G.; Bapat, V.; Rihal, C.; Vahanian, A.; John, W.; O'Neill, W. W.	2015	JACC: Cardiovascular Interventions	9(13)	1361-1371	No MAC thrombus, mitral valve intervention
347	Transcatheter Mitral Valve Replacement in Native Mitral Valve Disease With Severe Mitral Annular Calcification: Results From the First Multicenter Global Registry	Guerrero, M.; Dvir, D.; Himbert, D.; Urena, M.; Eleid, M.; Wang, D. D.; Greenbaum, A.; Mahadevan, V. S.; Holzhey, D.; O'Hair, D.; Dumonteil, N.; Rodes-Cabau, J.; Piazza, N.; Palma, J. H.; DeLago, A.; Ferrari, E.; Witkowski, A.; Wendler, O.; Kornowski, R.; Martinez-Clark, P.; Ciaburri, D.; Shemin, R.; Alnasser, S.; McAllister, D.;	2016	Journal of the American College of Cardiology	1)	B291-B292	No MAC thrombus, mitral valve intervention.

		Bena, M.; Kerendi, F.; Pavlides, G.; Sobrinho, J. J.; Attizzani, G. F.; George, I.; Nickenig, G.; Fassa, A. A.; Cribier, A.; Bapat, V.; Feldman, T.; Rihal, C.; Vahanian, A.; Webb, J.; O'Neill, W.						
348	Cavitating atrial myxoma mimicking hydatid cyst on echocardiography: Utility of cardiac magnetic resonance imaging and computed tomography for diagnosis and preoperative evaluation	Gupta, A.; Gulati, G. S.; Hote, M. P.; Ray, R.; Bahl, V. K.; Sharma, S.	2010	Journal of Thoracic Imaging	25(3)		W85-W88	Not MAC
349	Echocardiographic findings and the increased risk of stroke in nonvalvular atrial fibrillation	Gustafsson, C.; Britton, M.; Brolund, F.; Eriksson, S. V.; Lindvall, K.	1992	Cardiology	81	05-Apr	189-95	Not MAC thrombus
350	Big mitral annular calcification (Big MAC) - Dynamic liquefaction necrosis as a potential source of embolism	Haaf, P.; Hofmann, V.; Bremerich, J.; Zellweger, M.	2019	European Heart Journal Cardiovascular Imaging	20(Supplement 2)		ii69	Not MAC thrombus
351	Mitral Prosthetic Valve Assessment by Echocardiographic Guidelines	Hahn, R. T.	2013	Cardiology Clinics	31(2)		287-309	Mitral valve prosthesis
352	Pictorial review of cardiac calcification, pathophysiology, aetiology and imaging findings	Hajhosseiny, M.; Ariff, B.	2020	International Journal of Cardiovascular Imaging	36(11)		2097	Not MAC thrombus
353	Rare Presentation of Left Lower Lobe Pulmonary Artery Dissection	Hako, R.; Fedacko, J.; Toth, S.; Morochovic, R.; Kristian, P.; Pekarova, T.;	2017	Case Reports in Medicine	2017 (no pagination)			Not relevant, no MAC.

		Tuomainen, P.; Pella, D.						
354	Transcatheter aortic valve replacement: An update	Halim, S. A.; Kiefer, T. L.; Hughes, G. C.; Hurwitz, L. M.; Harrison, J. K.	2013	Current Cardiology Reports	15(6) (no pagination)			Not relevant, TAVR.
355	The echocardiogram in old age. [French]	Halphen, C.; Haiat, R.; Sliosberg, R.	1982	Concours Medical	104(35)		5019-5022	Not MAC thrombus
356	Spontaneous rupture of a caseous calcification of the mitral annulus in a hemodialysis patient	Hamasaki, A.; Uchida, T.; Sadahiro, M.	2017	Journal of Cardiac Surgery	32	2	85-87	Not MAC thrombus
357	Real-time 3d, 2d trans esophageal echocardiography for the evaluation of rheumatic mitral stenosis initial single center experience	Hameed, H. R. A.; Alsaad, S. F.; Almusaw, A. H. A.	2021	Archivos Venezolanos de Farmacologia y Terapeutica	40(2)		197-201	RHD
358	Ascites Due to Constrictive Pericardial Disease Not Appreciated on Echocardiogram: A Report of Three Cases	Han, S. H. B.; Yau, C.; Chin, E. E.	2018	Digestive Diseases and Sciences	63(3)		797-802	Not MAC thrombus Not relevant, pericardial disease.
359	Studying the Role of 3d Transthoracic Echocardiography for the Evaluation of Rheumatic Mitral Stenosis Compare to 2d Transesophageal Echocardiography	Hao, P. T.	2019	Journal of the American College of Cardiology	73(9 Supplement 1)		1601	RHD
360	Echocardiography in Detecting Mechanical Complications in Acute Coronary Syndrome	Harnish, P.; Nesheiwat, Z.; Mahmood, S.; Soni, R.; Eltahawy, E.	2020	Case	4(5)		393-398	No MAC thrombus
361	Capture of particulate emboli during cardiac procedures in which aortic cross-clamp is used	Harringer, W.	2000	Annals of Thoracic Surgery	70(3)		1119-1123	No MAC thrombus

362	Left atrial calcification. Review of literature and proposed management	Harthorne, J. W.; Seltzer, R. A.; Austen, W. G.	1966	Circulation	34	2	198-210	Not MAC thrombus
363	Current and future pharmacological treatment strategies with regard to aortic disease in Marfan syndrome	Hartog, A. W.; Franken, R.; Zwinderman, A. H.; Groenink, M.; Mulder, B. J. M.	2012	Expert Opinion on Pharmacothera py	13(5)		647-662	No MAC.
364	Spontaneous mitral valve perforation associated with mitral annular calcification-related calcified amorphous tumor assessed by three-dimensional transesophageal echocardiography	Haruki, N.; Sumi, N.; Kobara, S.; Tsujimoto, D.; Inoue, Y.; Saito, Y.; Shirota, K.	2020	Journal of Medical Ultrasonics	47(3)		481-482	Calcified amorphous tumor, not MAC thrombus
365	Mitral stenosis and regurgitation with systemic lupus erythematosus and antiphospholipid antibody syndrome	Hasegawa, R.; Kitahara, H.; Watanabe, K.; Kuroda, H.; Amano, J.	2001	Thoracic and Cardiovascular Surgeon. Conference: 39th Annual Meeting of the German Society for Cardiovascular and Thoracic Surgery. Stuttgart Germany. Conference Publication:	58	SUPP L. 1		Not MAC thrombus No MAC
366	Long term results of isolated aortic and mitral valve replacement with Medtronic Mosaik valve: 16 years outcome in a single center	Haschimi, A.; Costi, M.; Spiliopoulos, K.; Szolnoky, J.; Eichinger, W.	2010	Japanese Journal of Thoracic & Cardiovascular Surgery	49	12	711-3	Mitral valve prosthesis
367	Clinical benefit of simultaneous pulmonary vein isolation and percutaneous transvenous mitral commissurotomy in patients with mitral stenosis and atrial fibrillation	Hashimoto, N.; Arimoto, T.; Otaki, Y.; Tamura, H.; Nishiyama, S.; Takahashi, H.; Shishido, T.; Miyamoto, T.;	2017	Journal of Cardiac Failure	23(10 Supplement 1)		S34	Not MAC thrombus RHD

		Watanabe, T.; Kubota, I.						
368	Multimodality imaging of a giant caseous calcification of the mitral annulus	Hatipoglu Akpinar, S.; Bakal, R. B.; Guler, A.; Ozdemir, N.	2013	Turk Kardiyoloji Dernegi Arsivi	41	3	245-7	Not MAC thrombus
369	Transesophageal echocardiography in candidates for percutaneous balloon mitral valvuloplasty	Hausmann, D.; Daniel, W. G.; Heublein, B.; Mugge, A.; Zick, R.; Engel, H. J.; Lichtlen, P. R.	1994	Echocardiogra phy	11(6)		553-559	RHD, not MAC thrombus
370	Severe stenosis of bioprosthetic valve due to late valve thrombosis	Hegde, S.; Rahban, Y.; Agnihotri, A.; Maysky, M.	2020	Journal of Cardiac Surgery	35	11	3150-3152	Prosthetic valves
371	The durability and fate of aortic valve grafts. An experimental study with a long term follow-up of clinical patients	Heimbecker, R. O.; Aldrige, H. E.; Lemire, G.	1968	Journal of Cardiovascular Surgery	9	6	511-7	Not MAC thrombus Not relevant, aortic valve prosthesis
372	Open mitral commissurotomy: fourteen- to eighteen-year follow-up clinical study	Herrera, J. M.; Vega, J. L.; Bernal, J. M.; Rabasa, J. M.; Revuelta, J. M.	1993	Annals of Thoracic Surgery	55	3	641-5	Mitral valve intervention
373	A rare risk factor for embolic stroke: A 60-year-old male with caseous mitral annular calcification	Herrington, G.; Mora, A.	2021	Critical Care Medicine	49(1 SUPPL 1)		180	Not MAC thrombus
374	Ischemic stroke due to a calcified embolus from the mitral annular valve	Herskovitz, M.; Telman, G.; Carasso, S.; Symonovitz, A.; Goldsher, D.	2012	Neurology	78	12	931	No thrombus on MAC
375	Thrombosis and degeneration of Hancock valves: clinical and pathological findings	Hetzer, R.; Hill, J. D.; Kerth, W. J.; Wilson, A. J.; Adappa, M. G.; Gerbode, F.	1978	Annals of Thoracic Surgery	26	4	317-22	Mitral valve prosthesis

376	[Computed tomographic findings in mitral valve disease]	Heuser, L.; Neufang, K. F.; Jansen, W.	1984	Rofo: Fortschritte auf dem Gebiete der Rontgenstrahlen und der Nuklearmedizin	140	4	435-40	Not MAC thrombus
377	Outcome probabilities and life history after surgical mitral commissurotomy: implications for balloon commissurotomy	Hickey, M. S.; Blackstone, E. H.; Kirklin, J. W.; Dean, L. S.	1991	Journal of the American College of Cardiology	17	1	29-42	Mitral valve intervention
378	A case of caseous calcification of the mitral annulus: A potential source of embolic stroke	Higashi, H.; Ohara, T.; Nakatani, S.; Hashimoto, S.; Torii, T.; Miyashita, K.; Naritomi, H.; Kitakaze, M.	2010	Journal of Cardiology Cases	2	3	e141-e143	Not MAC thrombus
379	Application of Cerebral Protection System in Open Mitral Replacement with Extensive Calcified Left Atrial Thrombus	Ho, J. Y. K.; Chan, J. W. Y.; Chow, S. C. Y.; Yu, P. S. Y.; Kwok, M. W. T.; Cheung, G. S. H.; Wong, R. H. L.	2020	Innovations: Technology & Techniques in Cardiothoracic & Vascular Surgery	15	1	85-87	Valve intervention
380	Haemoptysis secondary to late rupture of a pseudo-aneurysm at the site of a corrected aortic coarctation	Hoffer, E.; Garau, G.; Henroteaux, D.; Sanoussi, A.	2021	Acta Cardiologica	76(8)		904-906	No MAC
381	Transesophageal echocardiography in patients with systemic arterial embolism. [German]	Hofmann, T.; Meinertz, T.	1993	Herz	18(5)		301-317	Not MAC thrombus
382	2012 ACCF/AATS/SCAI/STS Expert Consensus Document on Transcatheter Aortic Valve Replacement: Developed in collaboration with the American Heart Association, American Society of Echocardiography, European Association for Cardio-Thoracic Surgery, Heart Failure	Holmes Jr, D. R.; Mac, K. M. J.; Kaul, S.; Agnihotri, A.; Alexander, K. P.; Bailey, S. R.; Calhoon, J. H.; Carabello, B. A.; Desai, M. Y.; Edwards, F. H.;	2012	Annals of Thoracic Surgery	93(4)		1340-1395	Not MAC thrombus

	Society of America, Mended Hearts, Society of Cardiovascular Anesthesiologists	Francis, G. S.; Gardner, T. J.; Kappetein, A. P.; Linderbaum, J. A.; Mukherjee, C.; Mukherjee, D.; Otto, C. M.; Ruiz, C. E.; Sacco, R. L.; Smith, D.; Thomas, J. D.						
383	Value of cardiac CT-angiography in the diagnostic workup of patients with TIA and acute ischaemic stroke: Prevalence of cardioembolic risk sources and therapeutic implications	Holswilder, G.; Wermer, M.; Kroft, L.; De Roos, A.; Holman, E.; Kruyt, N.; Van Walderveen, M.	2018	European Stroke Journal	3(1 Supplement 1)		34	No thrombus on MAC
384	CT Angiography of the Heart and Aorta in TIA and Ischaemic Stroke: Cardioembolic Risk Sources and Clinical Implications	Holswilder, G.; Wermer, M. J.; Holman, E. R.; Kruyt, N. D.; Kroft, L. J.; van Walderveen, M. A.	2020	Journal of Stroke & Cerebrovascular Diseases	29	12	105326	Not MAC thrombus
385	[Diagnosis of left atrial masses by computed tomography: with special reference to the differentiation between mural thrombi and myxomas]	Hongo, M.; Okubo, S.; Amemiya, H.; Yamada, H.; Matsuoka, K.; Kusama, S.; Morimoto, M.	1983	Journal of Cardiography - Supplement	13	4	935-47	Not MAC thrombus
386	The Association of Calcific Valvular Disease and Retinal Embolization	Horn, P.; Genovese, P. D.	1964	Journal of the Indiana State Medical Association	57		227-9	Not MAC thrombus
387	Noninvasive diagnosis of complications of the mitral bioprosthesis	Horowitz, M. S.; Goodman, D. J.; Hancock, E. W.; Popp, R. L.	1976	Journal of Thoracic & Cardiovascular Surgery	71	3	450-7	Prosthetic valve
388	Prosthetic valves or tissue valves--a vote for mechanical prostheses	Horstkotte, D.	1985	Zeitschrift fur Kardiologie	74 Suppl 6		19-37	Prosthetic valve

389	Pathomorphological aspects, aetiology and natural history of acquired mitral valve stenosis	Horstkotte, D.; Niehues, R.; Strauer, B. E.	1991	European Heart Journal	12(SUPPL. B)		55-60	Rheumatic valve
390	Massive left atrial calcification: a case report and review of the literature	Hosseini, S.; Rezaei, Y.; Samiei, N.; Sadeghpour, A.; Peighambari, M. M.; Mestres, C. A.; Sellke, F. W.	2017	General Thoracic & Cardiovascular Surgery	65	11	653-656	Not MAC thrombus
391	Cerebrovascular myocardium-tissue embolism: a rare complication of heart surgery: autopsy case report	Hrudka, J.; Hlavicka, J.; Smulova, M. C.; Kujal, P.	2018	Cardiovascular Pathology	34		46-49	Valve intervention
392	Mitral valve replacement in children	Human, D. G.; Joffe, H. S.; Fraser, C. B.; Barnard, C. N.	1982	Journal of Thoracic & Cardiovascular Surgery	83	6	873-7	Not MAC thrombus
393	Short- and long-term results of catheter balloon percutaneous transvenous mitral commissurotomy	Hung, J. S.; Chern, M. S.; Wu, J. J.; Fu, M.; Yeh, K. H.; Wu, Y. C.; Cherng, W. J.; Chua, S.; Lee, C. B.	1991	American Journal of Cardiology	67	9	854-62	Valve intervention
394	Concurrent antegrade transseptal inoue-balloon mitral and aortic valvuloplasty	Hung, J. S.; Wu, C. J.; Chen, Y. L.; Lau, K. W.	2013	American Journal of Cardiology	1)		56B	Rheumatic valve, valve intervention
395	Reply to the editor	Hussain, S. T.; Blackstone, E. H.; Pettersson, G. B.	2013	Journal of Thoracic and Cardiovascular Surgery	146(6)		1557-1558	Not MAC thrombus
396	Reply to the editor	Hussain, S. T.; Idress, J.; Brozzi, N. A.; Blackstone, E. H.; Pettersson, G. B.	2013	Journal of Thoracic and Cardiovascular Surgery	146(5)		1301	Not MAC thrombus

397	[Late results of closed methods of the surgical treatment of multiple valvular rheumatic heart defects]	Iakimets, L. S.	1978	Kardiologiia	18	9	May-70	Rheumatic valve
398	Lambl's excrescences and cerebrovascular accident	Igel, L. I.	2012	Journal of General Internal Medicine	2)		S450	Not MAC thrombus
399	Long term clinical and haemodynamic evaluation of the Ionescu-Shiley pericardial xenograft heart valve	Ionescu, M. I.; Tandon, A. P.	1978	Thoraxchirurgie Vaskulare Chirurgie	26	4	250-8	Prosthetic valve
400	Radiological features of caseous calcification of mitral annulus	Ishchenko, M.; Svitlana, F.; Tregubova, M.; Vitkovskyi, Y.; Lutsenko, L.; Parfentyeva, V.	2021	International Journal of Cardiovascular Imaging	37(12)		3641-3643	Not MAC thrombus
401	Incidence of and risk factors for early perioperative neurocognitive disorder of mitral valve surgery	Ishida, K.; Murakami, S.; Morioka, T.; Yamashita, S.; Yamashita, A.; Matsumoto, M.	2021	Journal of Neurosurgical Anesthesiology	33(4)		419	Not MAC thrombus
402	Hepatic infarction associated with terminal hemorrhagic necrotizing enteropathy: A case report (Japanese). [Japanese]	Ishii, T.; Hosoda, Y.; Yakumaru, K.	1975	Japanese Journal of Geriatrics	12(6)		357-362	Not MAC thrombus
403	Transesophageal echocardiography findings in patients with embolic strokes of undetermined source	Ishizuka, K.; Hoshino, T.; Mizuno, S.; Toi, S.; Maruyama, K.; Uchiyama, S.; Kitagawa, K.	2016	Journal of Stroke & Cerebrovascular Diseases	27	3	697-702	Not MAC thrombus
404	Associations of Mitral and Aortic Valve Calcifications with Complex Aortic Atheroma in Patients with Embolic Stroke of Undetermined Source	Ishizuka, K.; Hoshino, T.; Ashihara, K.; Mruyama, K.; Toi, S.; Mizuno, S.; Shirai, Y.;	2018	European Stroke Journal	1(1 Supplement 1)		501-502	Not MAC thrombus

		Hagiwara, N.; Kitagawa, K.						
405	A stepwise aortic clamp procedure to treat porcelain aorta associated with aortic valve stenosis and hemodialysis	Isoda, S.; Osako, M.; Kimura, T.; Nishimura, K.; Yamanaka, N.; Nakamura, S.; Maehara, T.	2014	Annals of Thoracic & Cardiovascular Surgery	20 Suppl		725-9	Not MAC thrombus
406	Late pathological changes of Carpentier-Edwards porcine bioprostheses in the mitral position	Isomura, T.; Yanai, T.; Akagawa, H.; Aoyagi, S.; Kosuga, K.; Ohishi, K.; Koga, M.	1986	Journal of Cardiovascular Surgery	34	4	327-31	Prosthetic valve
407	Study on reoperation in cardiac disease. [Japanese]	Isomura, T.; Hisatomi, T.; Satou, R.; Matsuzoe, S.; Hirano, A.; Kosuga, K.; Ohishi, K.	1992	Kyobu geka	The Japanese journal of thoracic surgery. 45(3)		208-211	Valve intervention
408	Clinical study of 47 patients with reoperation after open mitral commissurotomy	Isomura, T.; Hisatomi, K.; Satoh, T.; Hirano, A.; Kosuga, K.; Ohishi, K.	1993	Journal of Cardiovascular Surgery	27	3	307-15	Valve intervention
409	Surgical treatment of mitral stenosis with a closed commissurotomy. [German]	Isringhaus, H.; Stapenhorst, K.	1976	Herz Kreislauf	8(10)		559-564	Not MAC thrombus
410	Cerebral hyperperfusion syndrome following the excision of a mycotic aneurysm with superficial temporal artery-to-middle cerebral artery bypass: Case report	Ito, H.; Tanaka, Y.; Sase, T.; Uchida, M.; Yoshida, Y.; Sakakibara, Y.; Hashimoto, T.	2014	Neurologia Medico-Chirurgica	54(10)		845-850	Not MAC thrombus
411	Surgical management of valvular heart diseases in pregnancy	Jafferani, A.; Malik, A.; Khawaja, R. D. A.; Sheikh, L.; Sharif, H.	2011	European Journal of Obstetrics and Gynecology and	159(1)		91-94	Not MAC thrombus

				Reproductive Biology				
412	Early clinical experience with the Carpentier-Edwards porcine heterograft cardiac valve	Jamieson, W. R. E.; Janusz, M. T.; Munro, A. I.	1980	Thoracic and Cardiovascular Surgeon	29(4)		200-205	Prosthetic valve
413	Clinical experience with the Carpentier-Edwards porcine xenograft	Jamieson, M. P. G.; Bennett, J. G.; Robles, A.; Ross, D. N.	1981	Canadian Journal of Surgery	23(2)		132-137	Prosthetic valve
414	St Jude Medical Epic porcine bioprosthesis: Results of the regulatory evaluation	Jamieson, W. R. E.; Lewis, C. T. P.; Sakwa, M. P.; Cooley, D. A.; Kshettry, V. R.; Jones, K. W.; David, T. E.; Sullivan, J. A.; Fradet, G. J.; Bach, D. S.	2011	Journal of Thoracic and Cardiovascular Surgery	141(6)		1449-1454	Prosthetic valve
415	"Mosaic" medtronic bioprosthetic valve replacement clinical results and hemodynamical performance	Jasinski, M. J.; Kadziola, Z.; Keal, R.; Sosnowski, A. W.	2000	Journal of Cardiovascular Surgery	41	2	181-6	Prosthetic valve
416	Valve Repair Is Superior to Replacement in Most Patients With Coexisting Degenerative Mitral Valve and Coronary Artery Diseases	Javadikasgari, H.; Gillinov, A. M.; Idrees, J. J.; Mihaljevic, T.; Suri, R. M.; Raza, S.; Houghtaling, P. L.; Svensson, L. G.; Navia, J. L.; Mick, S. L.; Desai, M. Y.; Sabik, J. F.; Blackstone, E. H.	2017	Annals of Thoracic Surgery	103(6)		1833-1841	Valve intervention
417	Mitral Valve Replacement with a Pulmonary Autograft in an Infant	Jeong, Y. H.; Yun, T. J.	2018	The Korean Journal of Thoracic & Cardiovascular Surgery	51	2	149-152	Valve intervention

418	Mitral annulus calcification and embolism	Jespersen, C. M.; Egeblad, H.	1987	Acta Medica Scandinavica	222	1	37-41	Not MAC thrombus
419	Calcified mitral ring. Incidence and complications. [Danish]	Jespersen, C. M.; Egeblad, H.	1987	Ugeskrift for laeger	149(50)		3394-3397	Not MAC thrombus
420	Transcatheter heart valve thrombosis on the mitral stage: the tempest or much ado about nothing"?	Jilaihawi, H.	2021	EuroInterventio n	16(17)		1386-1387	Valve intervention
421	Closed mitral valvotomy: Early results and long-term follow-up of 3724 consecutive patients	John, S.; Bashi, V. V.; Jairaj, P. S.; Muralidharan, S.; Ravikumar, E.; Rajarajeswari, T.; Krishnaswami, S.; Sukumar, I. P.; Rao, P. S.	1983	Circulation	68(5)		891-896	Valve intervention
422	Closed mitral valvotomy in the older subject	John, S.; Bashi, V. V.; Ravikumar, E.; Jairaj, P. S.; Krishnaswami, S.	1990	Journal of Cardiovascular Surgery	31(1)		14-19	Valve intervention
423	Ischemic stroke and ST-elevation myocardial infarction revealing infective endocarditis	Joliat, G. R.; Halkic, N.; Pantet, O.; Ben-Hamouda, N.	2017	European Review for Medical and Pharmacologic al Sciences	21(20)		4640-4641	Not MAC thrombus
424	The cardiovascular status of the black stroke patient	Joubert, J.; van Gelder, A. L.; Darazs, B.; Pilloy, W. J.	1989	South African Medical Journal. Suid-Afrikaanse Tydskrif Vir Geneeskunde	76	12	657-64	Not MAC thrombus
425	Functional and morphological cardiac changes in myeloproliferative disorders (clinical study)	Kadikoylu, G.; Onbasili, A.; Tekten, T.; Barutca, S.; Bolaman, Z.	2004	International Journal of Cardiology	97(2)		213-220	Not MAC thrombus
426	Hybrid Mitral Valve Replacement: An Emerging Option for	Kafil, T. S.; Manian, U.; Tzemos, N.	2019	JACC: Case Reports	1(4)		500-502	Not MAC thrombus

	Nonsurgical Candidates With Complex Mitral Valve Disease						
427	Kawasaki disease: origins and evolution	Kainth, R.; Shah, P.	2021	Archives of Disease in Childhood	106(4)	413-414	Not MAC thrombus
428	Should aortic valve calcification be checked before percutaneous coronary intervention?	Kaji, S.	2017	Journal of Atherosclerosis and Thrombosis	24(5)	475-476	Not MAC thrombus
429	Aortic Cusp Extension for Surgical Correction of Rheumatic Aortic Valve Insufficiency in Children	Kalangos, A.; Myers, P. O.	2013	World Journal for Pediatric and Congenital Heart Surgery	4(4)	385-391	Not MAC thrombus
430	Huge left atrium thrombus and chronic pulmonary artery thrombus due to critical mitral valve stenosis	Kalinin, A.; Trenin, A.	2010	Circulation	122(2)	e295-e296	Rheumatic valve
431	Cardiovascular findings on cross-sectional imaging: spectrum of incidental and critical findings and clinical relevance for the abdominal radiologist	Kalisz, K.; Enzerra, M.; Ansari-Gilani, K.	2019	Abdominal Radiology	44(3)	1161-1180	Not MAC thrombus
432	Mitral annular calcification and echogenic densities in the left-ventricular outflow tract in association with cerebral ischemic events	Kalman, P.; DePace, N. L.; Kotler, M. N.	1982	Journal of Cardiovascular Ultrasonograph y	1(2)	155-160	Not MAC thrombus
433	Mitral annular calcifications and aortic plaques as predictors of increased cardiovascular mortality	Kamensky, G.; Lisy, L.; Polak, E.; Piknova, E.; Plevova, N.	2001	Journal of Cardiology	37 Suppl 1	21-Jun	Not MAC thrombus
434	OCT findings in patients with recanalization of organized thrombi in coronary arteries	Kang, S. J.; Nakano, M.; Virmani, R.; Song, H. G.; Ahn, J. M.; Kim, W. J.; Lee, J. Y.; Park, D. W.; Lee, S. W.; Kim, Y. H.; Lee, C. W.;	2012	Journal of the American College of Cardiology	1)	1082	Not MAC thrombus

		Park, S. W.; Park, S. J.						
435	Calcified masses attached at the anterior and posterior leaflets of mitral valve in a patient hemodialysis	Kang, M. K.; Kim, K. I.	2016	JACC: Cardiovascular Imaging	5(7)		725-732	Not MAC thrombus
436	Transcatheter aortic valve implantation in a patient with severe aortic stenosis and femur shaft fracture	Karaduman, B. D.; Ayhan, H.; Keles, T.; Durmaz, T.; Akcay, M.; Bayram, N. A.; Bozkurt, E.	2019	Anatolian Journal of Cardiology	22(Supplement 1)		84	Not MAC thrombus
437	The two sides of the spectrum: Paravalvular mitral regurgitation	Karakulak, U. N.; Ozer, N.	2021	Anatolian Journal of Cardiology	25		E26-E27	Not MAC thrombus
438	An extensive caseous calcification of the mitral annulus complicated with severe mitral regurgitation	Karakus, A.; Tibilli, H.; Inanc, I. H.; Tasolar, H.; Turkmen, S.; Akturk, E.	2018	Echocardiogra phy	35	2	282-284	Not MAC thrombus
439	A Case of Multiple [1]ic Strokes in a Patient With a Porcelain Left Atrium	Karan, A.; Feghaly, J.; Guo, H. J.; Akinjogbin, T. O.; Sattiraju, S.	2021	Cureus	13	10	e18585	Not MAC thrombus
440	Relation between mitral annular calcium and complex aortic atheroma in patients with cerebral ischemia referred for transesophageal echocardiography	Karas, M. G.; Francescone, S.; Segal, A. Z.; Devereux, R. B.; Roman, M. J.; Liu, J. E.; Hahn, R. T.; Kizer, J. R.	2007	American Journal of Cardiology	99	9	1306-11	Not MAC thrombus
441	Closed mitral valvulotomy	Karkola, P.; Kairaluoma, M. I.; Larmi, T. K. I.	1976	Annales Chirurgiae et Gynaecologiae	65(2)		132-137	Not MAC thrombus
442	Calciphylaxis after parathyroidectomy	Karmegam, S.; Shetty, A.	2017	Hemodialysis International	21(Supplement 2)		S62-S66	Not MAC thrombus

443	A Calcified Amorphous Tumor Originating in the Aortic Valve Cusp	Kasai, M.; Osako, M.; Tanino, T.; Maeshima, A.	2018	Annals of Thoracic Surgery	105(6)		e235-e237	Not MAC thrombus
444	TCTAP C-230 A Super-advanced Aged Female Case with Early Infective Endocarditis After Transcatheter Aortic Valve Implantation Corroborating with the Autopsy	Kataoka, T.	2019	Journal of the American College of Cardiology	73(15 Supplement)		S289-S290	Not MAC thrombus
445	Symptoms and natural history in patients with isolated severe calcific mitral stenosis	Kato, N.; Padang, R.; Scott, C. G.; Guerrero, M.; Pislaru, S. V.; Pellikka, P. A.	2020	European Heart Journal	41(SUP PL 2)		1997	Not MAC thrombus
446	Calcific cerebral embolism in systemic calciphylaxis	Katsamakis, G.; Lukovits, T. G.; Gorelick, P. B.	1998	Neurology	51	1	295-7	Not MAC thrombus
447	Right ventricular endocardiectomy for loeffler's endocarditis presenting as cardiogenic shock	Katz, J. S.; Garcia, M.; Goldstein, D.; Murthy, S.; Sobolev, M.	2016	Journal of the American College of Cardiology	1)		1066	Not MAC thrombus
448	Pathological evaluation of massive left atrial calcification 35 years after mitral-aortic valve replacement	Kawakami, R.; Hao, H.; Kimura, N.; Komatsu, S.; Kodama, K.; Hirota, S.	2015	Journal of Cardiology Cases	11	2	44-47	Rheumatic valve
449	Aortic valve reconstruction with autologous pericardium for dialysis patients	Kawase, I.; Ozaki, S.; Yamashita, H.; Uchida, S.; Nozawa, Y.; Matsuyama, T.; Takatoh, M.; Hagiwara, S.	2013	Interactive Cardiovascular & Thoracic Surgery	16	6	738-42	Not MAC thrombus
450	Spontaneous echo contrast in the descending aorta in patients without aortic dissection: associated clinical and echocardiographic characteristics	Kaymaz, C.; Ozdemir, N.; Kirma, C.; Ozkan, M.	2003	International Journal of Cardiology	90	03-Feb	147-52	Not MAC thrombus

451	Alkaptonuria and renal failure: A case report	Kazancioglu, R.; Taylan, I.; Aksak, F.; Durak, H.; Kumbasar, B.; Yenigun, M.; Sar, F.	2004	Journal of Nephrology	17(3)		441-445	Not MAC thrombus
452	Simultaneous bilateral retinal artery occlusions associated with a mitral valve mass	Kedar, S.; Biousse, V.; Newman, N. J.	2005	Journal of Neuro-Ophthalmology	25	3	215-6	Not MAC
453	Guidelines for the prevention of stroke in patients with stroke and transient ischemic attack: A guideline for healthcare professionals from the American Heart Association/American Stroke Association	Kernan, W. N.; Ovbiagele, B.; Black, H. R.; Bravata, D. M.; Chimowitz, M. I.; Ezekowitz, M. D.; Fang, M. C.; Fisher, M.; Furie, K. L.; Heck, D. V.; Johnston, S. C.; Kasner, S. E.; Kittner, S. J.; Mitchell, P. H.; Rich, M. W.; Richardson, D.; Schwamm, L. H.; Wilson, J. A.	2014	Stroke	45(7)		2160-2236	Not MAC thrombus
454	The role of Trans Thoracic Echo (TTE) in refining the future risk of thrombo embolism in patients presenting with ischemic stroke/TIA	Khan, I. U.; Hameed, A.; Greany, D.; Murphy, S.; Cosgrave, J.	2011	Irish Journal of Medical Science	10)		S331-S332	Not MAC thrombus
455	Renal failure with a heavy heart: Extensive cardiac calcification in a young patient with renal disease	Khan, S.; Kasouridis, I.	2019	European Heart Journal Cardiovascular Imaging	20(Supplement 1)		i294	Not MAC thrombus
456	Calcific aortic valve and spontaneous embolic stroke: a review of literature	Khetarpal, V.; Mahajan, N.; Madhavan, R.; Batra, S.; Mopala, P.; Sagar, A.; Rapolu, P.;	2009	Journal of the Neurological Sciences	287	02-Jan	May-32	Not MAC thrombus

		Nangia, S.; Afonso, L.					
457	Incidence, clinical impact and treatment outcomes of valve thrombosis after transcatheter mitral valve implantation	Kikoine, J.; Urena, M.; Brochet, E.; Nguyen, C.; Carrasco, J. L.; Ducrocq, G.; Fuchs, A.; Vahanian, A.; Iung, B.; Himbert, D.	2020	European Heart Journal	41(SUP PL 2)	2630	Valve intervention
458	Valve thrombosis after transcatheter mitral valve implantation	Kikoine, J.; Urena, M.; Chong-Nguyen, C.; Fuchs, A.; Carrasco, J.; Brochet, E.; Ducrocq, G.; Vahanian, A.; Iung, B.; Himbert, D.	2021	Archives of Cardiovascular Diseases Supplements	13(1)	70-71	Valve intervention
459	Recurrent sudden sensorineural hearing loss in a patient with aortic aneurysm	Kilickaya, M. M.; Aynali, G.	2016	Journal of Clinical and Analytical Medicine	7(5)	720-722	Not MAC thrombus
460	A novel technique-Prophylactic septal radiofrequency ablation to prevent left ventricular outflow tract obstruction with transcatheter mitral valve replacement (RADIO-TMVR)	Killu, A. M.; Guerrero, M.; Siontis, K. C.; El Sabbagh, A.; Eleid, M. F.; Alkhouli, M.; Rihal, C.; Asirvatham, S. J.	2020	Journal of Cardiovascular Electrophysiology	31(11)	3048-3055	Not MAC thrombus
461	Various findings of cardiac thrombi on MDCT and MRI	Kim, D. H.; Il Choi, S.; Choi, J. A.; Chang, H. J.; Choi, D. J.; Lim, C.; Cho, J. H.; Park, J. H.	2006	Journal of Computer Assisted Tomography	30(4)	572-577	Not MAC thrombus

462	Hutchinson-Gilford progeria syndrome with G608G LMNA mutation	Kim, H. K.; Lee, J. Y.; Bae, E. J.; Oh, P. S.; Park, W. I.; Lee, D. S.; Kim, J. I.; Lee, H. J.	2011	Journal of Korean Medical Science	26	12	1642-5	Not MAC thrombus
463	Mechanical decalcification of the aortic valve	King, R. M.; Pluth, J. R.; Giuliani, E. R.; Piehler, J. M.	1986	Journal of the American College of Cardiology	77(18 Supplement 1)		2848	Not MAC thrombus
464	Massive Left Atrial Calcification, the Case of a Heart of Stone	King, N.; Jackson, C.; Zalawadiya, S.	2021	Annals of Thoracic Surgery	42(3)		269-272	Not MAC thrombus
465	Predictors of development of moderate-to-severe, and severe mitral regurgitation following percutaneous transseptal mitral commissurotomy using Accura balloon-The need to look beyond MGH (Wilkins) score	Kini, P.; Dash, P. K.; Barooah, B.; Reeta, V.; Sola, S.	2015	Indian Heart Journal	1)		S1	Valve intervention
466	Triple papillary fibroelastomas in an asymptomatic patient	Kireyev, D.; Ashraf, M. H.; Wilson, M. F.	2012	Cardiovascular Journal of Africa	23(7)		e7-e9	Not MAC thrombus
467	Subvalvular calcification of mitral valve	Kirk, R. S.; Russell, J. G.	1969	British Heart Journal	31	6	684-92	Publication before 1985
468	Comparison of thoracic aortic atherosclerotic plaque by echocardiography in patients with stenotic tricuspid versus bicuspid aortic valves	Kitaoka, H.; Okawa, M.; Kuwabara, M.; Hamada, T.; Yamasaki, N.; Matsumura, Y.; Furuno, T.; Doi, Y.	2003	American Journal of Cardiology	91(7)		895-896	Not MAC thrombus
469	Mitral balloon valvuloplasty by Inoue technique. A new emerging indication	Kiwan, Y.; Kasri, R.; Langar, J.; Haddad, N.; Bousnina, A.	1995	Saudi Heart Journal	6(1)		73-82	Not MAC thrombus
470	Mitral annular calcification	Klink, T.; Heverhagen, J. T.	2015	New England Journal of Medicine	372(17)		e23	Not MAC thrombus

471	Massive thrombosis of left atrium in surgery of isolated mitral valve disease: Plasty or replacement?	Knyshov, G. V.; Popov, V. V.; Sytar, L. L.; Mnishenko, V. I.	2011	Interactive Cardiovascular and Thoracic Surgery	1)		S121	Not MAC thrombus
472	Clinical features, problems in medical management and therapeutic planning in severe valvular heart disease	Kobayashi, K.; Fujitani, K.; Maeda, K.	1984	Circulation	102	19 Suppl 3	III30-4	Not MAC thrombus
473	Ten-year experience of chordal replacement with expanded polytetrafluoroethylene in mitral valve repair	Kobayashi, J.; Sasako, Y.; Bando, K.; Minatoya, K.; Niwaya, K.; Kitamura, S.	2000	Japanese Circulation Journal	48	10	1109-17	Valve intervention
474	An unusual echocardigraphic image of massive mitral annular calcification extending into left atrial wall	Kocaturk, H.; Colak, M. C.; Bayram, E.; Yilmaz, M.	2008	Anatolian Journal of Clinical Investigation	2(1)		40-42	Not MAC thrombus
475	Quantification of mitral annular calcification in octogenarian patients. Innocent bystander or marker of pathology!	Kochar, G.; Jacobs, L. E.; Blondheim, D. S.; Kotler, M. N.	1991	Echocardiography	8(3)		329-340	Not MAC thrombus
476	A case of aortic bicuspid valve with thrombus formation without severe stenosis and calcification	Koitabashi, T.; Inomata, T.; Kaida, T.; Shinagawa, H.; Shibata, K.; Miyaji, K.; Izumi, T.	2012	Journal of Echocardiography	10(4)		148-150	Not MAC thrombus
477	Echocardiographic criteria for choosing the method of operation in mitral stenosis. [Russian]	Konstantinov, B. A.; Zaretskii, V. V.; Kuznetsova, L. M.; Dadabaev, A. D.	1985	Kardiologiia	25(11)		96-98	Not MAC thrombus
478	Fate of left-sided cardiac bioprosthesis valves in children	Kopf, G. S.; Geha, A. S.; Hellenbrand, W. E.; Kleinman, C. S.	1986	Archives of Surgery	121	4	488-90	Prosthetic valve
479	[Mitral restenosis and its surgical treatment]	Korolev, B. A.; Karov, V. V.	1981	Kardiologiia	21	11	21-May	Valve intervention

480	Direct access valve-in-valve implantation for management of complex valvulopathy	Kossar, A. P.; Borger, M.; George, I.	2019	Catheterization and Cardiovascular Interventions	93(7)		1385-1388	Prosthetic valve
481	Expression of matrix metalloproteinases 1, 2, 9, 12 in xenogenic tissues of epoxy-crosslinked bioprosthetic heart valves explanted due to dysfunction. [Russian]	Kostyunin, A. E.; Glushkova, T. V.	2020	Russian Journal of Cardiology	25(10)		49-53	Not MAC thrombus
482	Noninvasive evaluation of normal and abnormal prosthetic valve function	Kotler, M. N.; Mintz, G. S.; Panidis, I.; Morganroth, J.; Segal, B. L.; Ross, J.	1983	Journal of the American College of Cardiology	2	1	151-73	Prosthetic valve
483	[Surgical treatment of mitral stenosis]	Kozlov, V. A.	1978	Kardiologiia	18	9	Jun-32	Valve intervention
484	Role of transesophageal echocardiography in the evaluation of patients with retinal artery occlusion	Kramer, M.; Goldenberg-Cohen, N.; Shapira, Y.; Axer-Siegel, R.; Shmuely, H.; Adler, Y.; Weinberger, D.; Sagie, A.	2001	Ophthalmology	108	8	1461-4	Not MAC thrombus
485	Calcific embolus causing subarachnoid hemorrhage	Kranick, S. M.; Levine, J. M.; Cucchiara, B. L.	2009	Neurology	73	3	245-6	Not MAC thrombus
486	[Heart valves in rheumatic heart disease in the light of scanning electron microscopy]	Krymskii, L. D.; Nestaiko, G. V.; Iaroshinskii Iu, N.	1975	Kardiologiia	15	11	57-61	Rheumatic valve
487	A case of Carpentier-Edwards pericardial bioprosthesis in mitral position explanted 22 years after implantation	Kubota, S.; Wakasa, S.; Ooka, T.; Tachibana, T.; Shiiya, N.; Matsui, Y.	2010	Annals of Thoracic Surgery	90	5	1692-4	Prosthetic valve

488	Cardiac swinging calcified amorphous tumors in end-stage renal failure patients	Kubota, H.; Fujioka, Y.; Yoshino, H.; Koji, H.; Yoshihara, K.; Tonari, K.; Endo, H.; Tsuchiya, H.; Mera, H.; Soga, Y.; Taniai, S.; Sakata, K.; Sudo, K.	2010	Journal of Artificial Organs	13(1)		48-50	Calcified amorphous tumor, not MAC thrombus
489	Experience with homograft mitral valve replacement	Kumar, A. S.; Kumar, D. A.; Chander, H.; Saxena, A.	1998	Journal of Heart Valve Disease	7(2)		225-228	Valve intervention
490	Results of mitral valve repair in rheumatic mitral regurgitation	Kumar, A. S.; Talwar, S.; Saxena, A.; Singh, R.; Velayoudam, D.	2006	Journal of Thoracic & Cardiovascular Surgery	138	2	359-64	Rheumatic valve
491	Mitral valve replacement with the pulmonary autograft: midterm results	Kumar, A. S.; Talwar, S.; Gupta, A.	2009	Interactive Cardiovascular and Thoracic Surgery	5(4)		356-361	Valve intervention
492	[Diagnostic value of transesophageal echocardiography in diseases of the heart valve system]	Kupferwasser, I.; Mohr-Kahaly, S.; Wittlich, N.; Meyer, J.	1993	Herz	18	5	290-300	Not MAC thrombus
493	[Mitral valve replacement for a severely calcified mitral annulus]	Kuriyama, M.; Kioka, Y.; Tanabe, A.	2013	Kyobu Geka - Japanese Journal of Thoracic Surgery	66	10	894-7	Not MAC thrombus
494	Multimodal diagnosis of caseous calcification of the mitral annulus in a patient with suspected left ventricular tumor	Kurnicka, K.; Samul, J.; Piotrowska-Kownacka, D.; Wisniewska, M.; Lipinska, A.; Pruszczyk, P.	2017	Polish Archives of Internal Medicine	127(4)		281-282	Not MAC thrombus

495	Factor VII hyperactivity and mitral ring calcification in elderly women. [Japanese]	Kuroda, T.; Kario, K.; Nishinaga, M.; Yamasawa, M.; Kim, S.; Goto, T.; Fukazawa, H.; Nagou, N.; Kayaba, K.; Shimada, K.	1995	Journal of Cardiology	26(SUPPL. 1)		45-50	Not MAC thrombus
496	An important complication of Hancock mitral valve replacement in children	Kutsche, L. M.; Oyer, P.; Shumway, N.; Baum, D.	1979	Circulation	60	2 Pt 2	98-103	Prosthetic valve
497	A study on cerebral embolism in mitral stenosis	Kwon, O.; Kim, M. H.; Kim, K. S.; Bae, J. H.; Kim, M. S.; Song, J. S.	1986	Korean Journal of Internal Medicine	1	1	Jun-60	Not MAC thrombus
498	Cardiac masses in echocardiography: A pragmatic review	L'Angiocola, P. D.; Donati, R.	2020	Journal of Cardiovascular Echography	30(1)		14-May	Not MAC thrombus
499	Calcific deposits in stenotic mitral valves. Extent and relation to age, sex, degree of stenosis, cardiac rhythm, previous commissurotomy and left atrial body thrombus from study of 164 operatively-excised valves	Lachman, A. S.; Roberts, W. C.	1978	Circulation	57	4	808-15	Not MAC thrombus
500	Aortic valve calcification using multislice CT	Laissy, J. P.; Messika-Zeitoun, D.; Cueff, C.; Pasi, N.; Serfaty, J. M.; Vahanian, A.	2011	Imaging in Medicine	3(3)		313-320	Not MAC thrombus
501	Prevalence and involvement of different valves in rheumatic heart disease - An observational echocardiographic study in a tertiary care center, Bengaluru, India	Lalitha, S.; Sai, V.; Pasam, P.; Bhargavi, V.	2021	Journal of Clinical and Diagnostic Research	15(8)		OC63-OC66	Rheumatic valve
502	Calcific stenosis of the porcine heterograft	Lamberti, J. J.; Wainer, B. H.; Fisher, K. A.;	1979	Annals of Thoracic Surgery	28	1	28-32	Prosthetic valve

		Karunaratne, H. B.; Al-Sadir, J.					
503	Commentary: Thinking outside the (cardiac)box	Langer, N. B.; Takayama, H.	2019	Journal of Thoracic and Cardiovascular Surgery	157(6)	e363-e364	Not MAC thrombus
504	Transesophageal echocardiography in the selection of patients prior to percutaneous mitral valvuloplasty. Study of 71 consecutive patients. [Spanish]	Laraudogoitia, E.; Medina, A.; Ortega, J. R.; Coello, I.; Hernandez, E.; Melian, F.; Jimenez, F.; Morales, J.	1991	Revista espanola de cardiologia	44(9)	599-604	Not MAC thrombus
505	Calcific embolization with infective endocarditis involving the posterior mitral leaflet in a patient with underlying hypertrophic obstructive cardiomyopathy	Lather, N.; Niziolek, K.; Toth, P.; Harris, D. M.	2015	Journal of Thrombosis and Thrombolysis	39(2)	241-244	Not MAC thrombus
506	Left ventricular mass	Laymouna, R.; Elsharkawy, E.; Morsy, Y.; Elshafei, M.; Elgowelly, M.; Almaghraby, A.	2016	European Heart Journal Cardiovascular Imaging	17(Supplement 2)	ii114	Not MAC thrombus
507	Caseous Calcification of the Mitral Annulus Associated with Embolic Infarcts and Chronic Pericardial Calcification	Layoun, M.; Thoms, T.; Fuss, C.; Stecker, E.; Khan, A.	2019	Journal of the American College of Cardiology	73(9 Supplement 1)	2250	No thrombus on MAC
508	[Canalized Massive Thrombosis with Parietal Calcifications of the Left Auricle]	Ledamany, L.; Gouffault, J.; Guillou, M.	1964	Archives des Maladies du Coeur et des Vaisseaux	87(10)	1275-1280	Not MAC thrombus
509	[Valvuloplasty with balloon catheter in biologic prosthesis. Reality or illusion]	Ledesma Velasco, M.; Verdin Vazquez, R.; Acosta Valdez, J. L.; Munayer Calderon, J.; Salgado Escobar, J. L.; Arias	1989	Semaine des Hopitaux	40	792-9	Prosthetic valve

		Monroy, L.; Flores Mendoza, J.						
510	Value of quantification of mitral valve disease by transoesophageal echocardiography before percutaneous mitral commissurotomy. [French]	Le Feuvre, C.; Mercier, L. A.; Lachurie, M. L.; Metzger, J. P.; Burelle, D.; Petitclerc, R.; Vacheron, A.; Bonan, R.	1994	Archivos del Instituto de Cardiologia de Mexico	59	1	69-71	Not MAC thrombus
511	Left ventricular diastolic abnormalities other than valvular disease in antiphospholipid syndrome: An echocardiographic study	Lembo, M.; Tufano, A.; Nardo, A.; Buonauro, A.; Fazio, V.; Schiano-Lomoriello, V.; Santoro, C.; Cocozza, S.; Di Minno, G.; Trimarco, B.; Galderisi, M.	2016	European Heart Journal Cardiovascular Imaging	17(Supplement 2)		ii67	Not MAC thrombus
512	Morphological findings in 192 surgically excised native mitral valves	Leong, S. W.; Soor, G. S.; Butany, J.; Henry, J.; Thangaroopan, M.; Leask, R. L.	2006	Canadian Journal of Cardiology	22(12)		1055-1061	Not MAC thrombus
513	Cardiac valves in patients with Whipple endocarditis: microbiological, molecular, quantitative histologic, and immunohistochemical studies of 5 patients	Lepidi, H.; Fenollar, F.; Dumler, J. S.; Gauduchon, V.; Chalabreysse, L.; Bammert, A.; Bonzi, M. F.; Thivolet-Bejui, F.; Vandenesch, F.; Raoult, D.	2004	Journal of Infectious Diseases	190	5	935-45	Not MAC thrombus
514	Position-related factors in mitral and tricuspid bioprostheses degenerative changes	Leprince, P.; Nataf, P.; Bors, V.; Ramadan, R.; Dorent, R.; Jault, F.; Coignard, L.; Fontanel, M.; Pavie, A.; Cabrol,	1997	Journal of Cardiovascular Surgery	38	3	223-6	Prosthetic valve

		C.; Gandjbakhch, I.						
515	[Surgical mitral valvuloplasty in the treatment of mitral valve diseases]	Lessana, A.; Palsky, E.; Romano, M.; Carbone, C.; Lomama, E.; Herrera, M.; Iles, F.; Merad, K.; Feghoul, M.; Jegier, B.; et al.,	1990	Revue du Praticien	40	24	Sep-34	Valve intervention
516	Percutaneous balloon valvuloplasty in acquired mitral and aortic stenosis in the adult. [French]	Letac, B.; Cribier, A.; Berland, J.	1988	Schweizerische Medizinische Wochenschrift	118(46)		1673-1680	Not MAC thrombus
517	Antithrombotic therapy in valvular heart disease	Levine, H. J.; Pauker, S. G.; Salzman, E. W.	1986	Chest	89	2 Suppl	36S-45S	Not case of MAC thrombus
518	Antithrombotic therapy in valvular heart disease	Levine, H. J.; Pauker, S. G.; Salzman, E. W.; Eckman, M. H.	1992	Chest	102(4 SUPPL.)		434S-444S	Not case of MAC thrombus
519	Cerebral, myocardial and cutaneous ischemic necrosis associated with calcific emboli from aortic and mitral valve calcification in a patient with end-stage renal disease	Li, Y.; Muench, A.; McGregor, D. H.; Wiegmann, T. B.	2002	Journal of the American Society of Echocardiography	34(10)		1056-1066	Rheumatic valve
520	Association of Global Cardiac Calcification with Atrial Fibrillation and Recurrent Stroke in Patients with Embolic Stroke of Undetermined Source	Li, T. Y. W.; Yeo, L. L. L.; Ho, J. S. Y.; Leow, A. S.; Chan, M. Y.; Dalakoti, M.; Chan, B. P. L.; Seow, S. C.; Kojodjojo, P.; Sharma, V. K.; Tan, B. Y. Q.; Sia, C. H.	2021	Clinical Nephrology	57	6	468-73	Not MAC thrombus

521	Evaluation of 25[#] ultramicroporous expanded polytetrafluoroethylene mitral valve pulsatile flow in vitro. [Chinese]	Liang, Y.; Wang, W. J.; Cai, K. C.; Li, H. B.	2008	Journal of Clinical Rehabilitative Tissue Engineering Research	12(14)	2667-2670	Prosthetic valve
522	Deadly Pulmonary Hypertension Cured With a Stent	Lightsey, J. M.; Boler, M. T.; Winscott, J. G.	2020	JACC: Case Reports	2(10)	1515-1519	Not MAC thrombus
523	Calcification of cardiac valves and arterio venous fistula thrombosis,what stands behind?	Likaj, E.; Seferi, S.; Caco, G.; Petrela, E.; Barbullushi, M.; Idrizi, A.; Thereska, N.	2014	Nephrology Dialysis Transplantation	3)	iii255-iii256	Not MAC thrombus
524	Incidental cardiac abnormalities on non-electrocardiogram-gated multi-detector computed tomography imaging of the thorax and abdomen	Lim, K. C.; Chai, P.; Teo, L. S. L.	2011	Singapore Medical Journal	52(12)	906-913	Not MAC thrombus
525	Survivin expression in cardiac myxoma	Lin, Y. S.; Jung, S. M.; Wu, H. H.; Shiu, T. F.; Tzai, F. C.; Chu, J. J.; Lin, P. J.; Chu, P. H.	2011	Chang Gung Medical Journal	34(4)	360-366	Not MAC thrombus
526	Executive summary: Heart disease and stroke statistics-2010 update: A report from the american heart association	Lloyd-Jones, D.; Adams, R. J.; Brown, T. M.; Carnethon, M.; Dai, S.; De Simone, G.; Ferguson, T. B.; Ford, E.; Furie, K.; Gillespie, C.; Go, A.; Greenlund, K.; Haase, N.; Hailpern, S.; Ho, P. M.; Howard, V.; Kissela, B.; Kittner, S.; Lackland, D.; Lisabeth, L.;	2010	Circulation	121(7)	e46-e215	Not MAC thrombus

		Marelli, A.; McDermott, M. M.; Meigs, J.; Mozaffarian, D.; Mussolino, M.; Nichol, G.; Roger, V. L.; Rosamond, W.; Sacco, R.; Sorlie, P.; Stafford, R.; Thom, T.; Wasserthiel-Smoller, S.; Wong, N. D.; Wylie-Rosett, J.						
527	The Mitroflow pericardial valve. First five years of follow-up evaluation	Loisance, D.; Zouari, M.; Leandri, J.; Hillion, M. L.; Cachera, J. P.	1989	ASAIO Transactions	35	3	304-7	Not MAC thrombus
528	Chronic renal disease: Infective endocarditis, sepsis and then calciphylaxis: The final stepping stone in chronic renal disease?	Loughlin, E.; Bergin, E.	2015	Irish Journal of Medical Science	1)		S330	Not MAC thrombus
529	Retinal embolus in a patient with mechanical mitral prosthesis	Loureiro, M.; Bilhoto, M.; Saraiva, E.; Sepulveda, P.	2016	Ophthalmic Research	56(Supplement 1)		10	Prosthetic valve
530	Early results of a real-world series with two transapical transcatheter mitral valve replacement devices	Ludwig, S.; Kalbacher, D.; Schofer, N.; Schafer, A.; Koell, B.; Seiffert, M.; Schirmer, J.; Schafer, U.; Westermann, D.; Reichenspurner, H.; Blankenberg, S.; Lubos, E.; Conradi, L.	2021	Clinical Research in Cardiology	110(3)		411-420	Valve intervention

531	Calcified amorphous tumor: A rare cause of central retinal artery occlusion	Ma, J. H.; Gill, M. K.	2018	Biomaterials	275 (no pagination)			Calcified amorphous tumor, not MAC thrombus
532	A multi-in-one strategy with glucose-triggered long-term antithrombogenicity and sequentially enhanced endothelialization for biological valve leaflets	Ma, B.; Liu, K.; Luo, R.; Wang, Y.	2021	American Journal of Ophthalmology Case Reports	10		25-27	Prosthetic valve
533	A morphologic study of Carpentier-Edwards pericardial xenografts in the mitral position exhibiting primary tissue failure in adults in comparison with Ionescu-Shiley pericardial xenografts	Machida, H.; Ishibashi-Ueda, H.; Nakano, K.; Sasako, Y.; Kobayashi, J.; Bando, K.; Minatoya, K.; Imamura, H.; Kitamura, S.	2001	Journal of Thoracic & Cardiovascular Surgery	122	4	649-55	Prosthetic valve
534	How to avoid problems in redo coronary artery bypass surgery	Machiraju, V. R.	2004	Journal of Cardiac Surgery	19(4)		284-290	Not MAC thrombus
535	Rock-Hard Chronic Thrombotic Occlusion and Its Management in Endovascular Interventions	Madassery, S.	2018	Seminars in Interventional Radiology	35(5)		461-468	Not MAC thrombus
536	Echocardiographic predictors of thromboembolic events in patients with left ventricular non compaction: Pulmonary hypertension is a risk factor	Mahajan, N.; Ganguly, J.; Simegn, M.; Bhattacharya, P.; Shankar, L.; Madhavan, R.; Chaturvedi, S.; Ramappa, P.; Afonso, L.	2009	Journal of the American Society of Echocardiography	1)		551	Not MAC thrombus
537	The legend of a stone heart	Maia Rusu, M.; Ionescu, A.; Constantinescu, T.; Barascu, T.; Popescu, B. A.; Ginghina, C.; Jurcut, R.	2018	European Journal of Heart Failure	20(Supplement 1)		169-170	Not MAC thrombus

538	Mitral valve-in-valve, valve-in-ring, and valve-in-MAC: The Good, the Bad, and the Ugly	Maisano, F.; Taramasso, M.	2019	European Heart Journal	40(5)		452-455	Not MAC thrombus
539	Case 36-2020: A 72-year-old woman with dark urine and weakness	Malhotra, R.; Namasivayam, M.; Ghoshhajra, B. B.; Passeri, J. J.; Hoenig, P. A.; Stone, J. R.	2020	New England Journal of Medicine	383(21)		2066-2076	Rheumatic valve
540	Transthoracic Echocardiography: Pitfalls and Limitations as Delineated at Cardiac CT and MR Imaging	Malik, S. B.; Chen, N.; Parker, R. A., 3rd; Hsu, J. Y.	2017	Radiographics	37	2	383-406	Not MAC thrombus
541	Bacterial endocarditis of the mitral valve associated with annular calcification	Mambo, N. C.; Silver, M. D.; Brunsdon, D. F. V.	1978	Canadian Medical Association Journal	119(4)		323-326	Not MAC thrombus
542	Efficacy and saftey of Balloon mitral valvotomy in elderly patients aged more than 65 years	Mane, V.; Makhale, C. N.; Hiremath, M. S.; Durairaj, M.; Sathe, S.; Vaidya, S.; Momin, H.; Parikh, J.	2017	Catheterization and Cardiovascular Interventions	89(Supplement 2)		S193	Rheumatic valve
543	Ruptured caseous mitral annular calcification presenting as a mobile mass	Manikat, R.; Elmi, F.; Shirani, J.	2015	Cardiology (Switzerland)	2)		396	Not MAC thrombus
544	The clinical life history of explanted prosthetic heart valves	Marbarger, J. P., Jr.; Clark, R. E.	1982	Annals of Thoracic Surgery	34	1	22-33	Prosthetic valve
545	Coronary Thrombosis and Type A Aortic Dissection	Marchetti, M.; Scacciatella, P.; Di Rosa, E.; Rinaldi, M.; Marra, S.	2015	Journal of Cardiac Surgery	30(7)		583-585	Not MAC thrombus
546	[Open mitral commissurotomy. Indications and results]	Maronas, J. M.; O'Conner, F.; Rufilanchas, J. J.; Tellez, G.; Agosti, J.; Figuera, D.	1975	Archivos del Instituto de Cardiologia de Mexico	45	4	495-502	Valve intervention

547	Embolic strokes of undetermined sources: Data from a stroke unit registry	Masina, M.; Cicognani, A.; Lofiego, C.; Malservisi, S.; Parlangeli, R.; Lombardi, A.	2015	International Journal of Stroke	2)		265-266	Not MAC thrombus
548	Caseous calcification of the mitral valve annulus: a rare cause of bilateral cerebral and ocular embolisation	Matsou, A.; Symeonidis, C.; Dermenoudi, M.; Sachpekidis, V.	2018	BMJ Case Reports	18		18	Not MAC thrombus
549	Critical multi-organ emboli originating from collapsed, vulnerable caseous mitral annular calcification	Matsuyama, T. A.; Ishibashi-Ueda, H.; Ikeda, Y.; Nagatsuka, K.; Miyashita, K.; Amaki, M.; Kanzaki, H.; Kitakaze, M.	2012	Pathology International	62	7	496-9	Not MAC thrombus
550	Yield of Transthoracic Echocardiogram (TTE) in identifying Cardiac Source of Embolism (CSOE) in patients with Ischaemic Cerebrovascular Accident (ICVA)	Mazhar, J.; Lee, E.; Davis, M.	2011	Heart Lung and Circulation	2)		S198	Not MAC thrombus
551	Bilateral axillary artery inflow in the treatment of a rare case of pseudocoartaction of the aortic arch	Mazzola, A.; Gregorini, R.; De Curtis, G.; Ciocca, M.	2007	Interactive Cardiovascular and Thoracic Surgery	6(5)		652-653	Not MAC thrombus
552	Acute stroke in a patient with advanced uraemia: Should thrombolysis be given?	McCloskey, M.; Masengu, A.; Shields, J.; Wiggam, M. I.	2013	BMJ Case Reports	(no pagination)			Not MAC thrombus
553	Biological Equivalence of GGTA-1 Glycosyltransferase Knockout and Standard Porcine Pericardial Tissue Using 90-Day Mitral Valve Implantation in Adolescent Sheep	McGregor, C.; Salmonsmith, J.; Burriesci, G.; Byrne, G.	2021	Cardiovascular Engineering & Technology	24		24	Not MAC thrombus
554	Successful surgical repair in a patient with mitral stenosis, calcified left atrium and severe	McKenney, R. N.; Melvin, K.	1987	Canadian Journal of Surgery	30	4	264-5	Not MAC thrombus

	tricuspid regurgitation with a giant right atrium							
555	Cardiac Surgery and Heparin Induced Thrombocytopaenia (HIT): A Case Report and Short Review	McMeniman, W. J.; Chard, R. B.; Norrie, J.; Posen, J.	2012	Heart Lung and Circulation	21(5)		295-299	Not MAC thrombus
556	North American multicenter experience with the On-X prosthetic heart valve	McNicholas, K. W.; Ivey, T. D.; Metras, J.; Szentpetery, S.; Marra, S. W.; Masters, R. G.; Dilling, E. W.; Slaughter, M. S.; Mack, M. J.	2006	Journal of Heart Valve Disease	15	1	73-8; discussion 79	Prosthetic valve
557	Current Status and Future Direction of Transcatheter Mitral Valve Replacement	Meng, Z.; Zhang, E. L.; Wu, Y. J.	2018	Chinese Medical Journal	131(5)		505-507	Not MAC thrombus
558	Patients with indications of open mitral commissurotomy undergoing mitral valve replacement. [Spanish]	Mesa, A.; Rebollar, L.; Quijano, F.	1983	Archivos del Instituto de Cardiologia de Mexico	53(4)		337-342	Valve intervention
559	Delayed left atrial wall dissection after mitral valve replacement	Messaoud, I.; Deville, C.; Roudaut, R.	2000	Echocardiography	17(3)		259-261	Valve intervention
560	Evaluation of mitral stenosis in 2008	Messika-Zeitoun, D.; Lung, B.; Brochet, E.; Himbert, D.; Serfaty, J. M.; Laissy, J. P.; Vahanian, A.	2008	Archives of Cardiovascular Diseases	101(10)		653-663	Not MAC thrombus
561	Recurrent unilateral pleural effusions. an atypical presentation of rheumatic mitral valve stenosis	Meyers, M.; De Bruijn, S.; Verhelst, J.; Prihadi, E.	2018	Acta Clinica Belgica: International Journal of Clinical and Laboratory Medicine	73(Supplement 2)		65	Rheumatic valve

562	The stentless quadrileaflet bovine pericardial mitral valve: early clinical results	Middlemost, S. J.; Sussman, M.; Patel, A.; Manga, P.	1999	Journal of Heart Valve Disease	8	2	174-9	Prosthetic valve
563	Infective endocarditis on mitral annular calcification: A case report	Minardi, G.; Pino, P. G.; Sordi, M.; Pavaci, H.; Manzara, C.; Pulignano, G.; Natale, E.; Gaudio, C.	2009	Cases Journal	2(11) (no pagination)			Not MAC thrombus
564	Long-term pathological changes of expanded polytetrafluoroethylene (ePTFE) suture in the human heart	Minatoya, K.; Kobayashi, J.; Sasako, Y.; Ishibashi-Ueda, H.; Yutani, C.; Kitamura, S.	2001	Journal of Heart Valve Disease	10(1)		139-142	Not MAC thrombus
565	Undiagnosed Type IIIc Gaucher Disease in a Child With Aortic and Mitral Valve Calcification: Perioperative Complications After Cardiac Surgery	Mireles, S. A.; Seybold, J.; Williams, G.	2010	Journal of Cardiothoracic and Vascular Anesthesia	24(3)		471-474	Not MAC thrombus
566	Mitral reconstruction in patients with infective endocarditis. [Japanese]	Mizuno, T.; Amano, J.; Sakamoto, T.; Suzuki, A.; Sunamori, M.; Tanaka, H.; Arai, H.; Shirai, T.; Watanabe, M.; Sugano, T.	1996	[Zasshi] [Journal]	Nihon Kyobu Geka Gakkai. 44(10)		1840-1846	Not MAC thrombus
567	Mitral annulus calcareous brain emboli	Mohammadkhani, M.; Schaefer, P.; Koroshetz, W.; Hedley-Whyte, E. T.	2000	Neurology	54	4	Mar-02	Not MAC thrombus
568	Double outlet right ventricle with calcified rheumatic mitral stenosis	Mohan, J. C.; Arora, R.; Khalilullah, M.	1991	Indian Heart Journal	43	5	397-9	Rheumatic Not MAC thrombus

569	Caseous Mitral Annular Calcification Mimicking Left Ventricular Outflow Tract Mobile Mass on Echocardiography in Patient with Cerebral Embolism	Mohsen, A.; Barrientos, J.; Falco, V.; Jain, N.	2020	Journal of the American College of Cardiology	75(11)		3404	Not MAC thrombus
570	Valvular heart disease: Better knowledge of biology and pathophysiology. [French]	Monsuez, J. J.	2002	Archives des Maladies du Coeur et des Vaisseaux - Pratique		113	15-16	Not MAC thrombus
571	Calcific embolization after percutaneous mitral valvuloplasty. [French]	Montalescot, G.; Drobinski, G.; Thomas, D.; Bletry, O.; Delcourt, A.; Evans, J.; Isnard, R.; Kieffer, E.; Grosgogeat, Y.	1992	Archives des Maladies du Coeur et des Vaisseaux	85(6)		905-907	Valve intervention
572	[Transcatheter cerebral embolic protection in open heart surgery: our initial experience in Ancona, Italy]	Montecchiani, L.; Alfonsi, J.; Cefarelli, M.; Berretta, P.; Capestro, F.; Di Eusanio, M.	2020	Giornale Italiano di Cardiologia	21	11	896-899	Not MAC thrombus
573	3 dimensional echocardiographic evaluation of prosthetic valve dysfunction	More, R.; Prajapati, J.; Chaudhary, S.; Sahoo, S.; Shinde, A.; Deshmukh, J.	2015	Indian Heart Journal	1)		S8	Prosthetic valve
574	Morbidity and mortality in patients undergoing mitral valve replacement at a cardiovascular surgery referral service: A retrospective analysis	Moreira, J. L.; Barletta, P. H. A. A. S.; Baucia, J. A.	2021	Brazilian Journal of Cardiovascular Surgery	36(2)		183-191	Not MAC thrombus
575	[3 cases of mital valve diseases with left atrial calcification]	Mori, M.; Naito, Y.; Horiguchi, Y.; Danno, M.; Kawashima, Y.	1969	Kyobu Geka - Japanese Journal of Thoracic Surgery	22	6	437-42	Not MAC thrombus
576	Successful surgical management for aortic and mitral stenosis in homozygous familial	Morimoto, N.; Okada, K.; Okita, Y.	2011	General Thoracic and	59(7)		491-494	Not MAC thrombus

	hypercholesterolemia: A case report			Cardiovascular Surgery				
577	Mitral bioprosthetic valve stenosis in a patient with antiphospholipid antibody syndrome and systemic lupus erythematosus	Morisaki, A.; Hirai, H.; Sasaki, Y.; Hosono, M.; Sakaguchi, M.; Nakahira, A.; Seo, H.; Suehiro, S.	2012	General Thoracic & Cardiovascular Surgery	60	12	822-6	Prosthetic valve
578	Calcified amorphous tumors with excision in hemodialysis patients: report of 2 cases. [Japanese]	Morishima, A.; Sasahashi, N.; Ueyama, K.	2006	Kyobu geka	The Japanese journal of thoracic surgery. 59(9)		851-854	Calcified amorphous tumor, not MAC thrombus
579	Effect of percutaneous mitral balloon valvuloplasty on mean platelet volume among patients with mitral stenosis	Mostafa, A. S. H.; Mousa, W. F.; Emera, A.; Magdy, A.	2018	Journal of Invasive Cardiology	30(9)		E80	Rheumatic valve
580	Caseous calcification of the mitral valve complicated by embolization, mitral regurgitation, and pericardial constriction	Motwani, M.; Fairbairn, T. A.; Jogiya, R.; Greenwood, J. P.; Plein, S.	2012	European Heart Journal Cardiovascular Imaging	13(9)		792	Not MAC thrombus
581	Ischemic stroke due to calcific emboli from mitral valve annulus calcification [5] (multiple letters)	Mouton, P.; Biousse, V.; Crassard, I.; Bousson, V.; Bousser, M. G.; Stein, J. H.; Soble, J. S.	1997	Stroke	28(11)		2325-2326	No thrombus on MAC
582	Complications of the calcified mitral anulus fibrosus. [German]	Mueller, F.; Schneider, J.	1980	Schweizerische Medizinische Wochenschrift	110(34)		1233-1244	Publication before 1985
583	Value of transesophageal 3D echocardiography as an adjunct to conventional 2D imaging in preoperative evaluation of cardiac masses	Muller, S.; Feuchtner, G.; Bonatti, J.; Muller, L.; Laufer, G.; Hiemetzberger, R.; Pachinger, O.; Barbieri, V.; Bartel, T.	2008	Echocardiography	25	6	624-31	Not MAC thrombus

584	Aortic and mitral valve replacement with the Carpentier-Edwards pericardial bioprosthesis: 10-year results	Murakami, T.; Eishi, K.; Nakano, S.; Kobayashi, J.; Sasako, Y.; Isobe, F.; Kosakai, Y.; Kito, Y.; Kawashima, Y.	1996	Journal of Heart Valve Disease	5	1	Sep-45	Prosthetic valve
585	Immediate outcomes of treatment of severe mitral annular calcification. [Russian]	Muratov, R. M.; Sorcomov, M. N.; Sachkov, A. S.; Babenko, S. I.; Sleptsova, A. M.; Terekhov, M. I.	2021	Vestnik Transplantologi i i Iskusstvennykh Organov	23(2)		52-59	Valve intervention
586	Fifth double-valve replacement in a boy with atrioventricular canal and Shone's complex	Murthy, R. A.; Cocalis, M.; Lamberti, J. J.	2018	Innovations: Technology and Techniques in Cardiothoracic and Vascular Surgery	13(Supplement 3)		S61-S62	Not MAC thrombus
587	Outcomes in patients with triple vessel disease undergoing coronary artery bypass graft	Murugesan, G.; Lysander, A.	2016	Journal of the American College of Cardiology	1)		S24	Not MAC thrombus
588	Patent foramen ovale as a risk factor in strokes	Mutlu, H.; Kucukoglu, M. S.; Yigit, Z.; Kucukoglu, H.; Okcun, B.; Bavcic, A.; Uner, S.	1999	Cardiovascular Imaging	11(2)		77-81	Not MAC thrombus
589	Mitral tendon prolapsing into the left ventricular outflow tract	Myers, P. O.; Cikirikcioglu, M.; Lerch, R.; Didier, D.; Kalangos, A.	2007	Journal of Cardiovascular Surgery	48(6)		801-803	Not MAC thrombus
590	Transcatheter heart valve implantation for failing surgical bioprostheses: Technical considerations and evidence for valve-in-valve procedures	Mylotte, D.; Lange, R.; Martucci, G.; Piazza, N.	2013	European Heart Journal	36	21	1306-27	Previous valve intervention

591	Transcatheter heart valve failure: a systematic review	Mylotte, D.; Andalib, A.; Theriault-Lauzier, P.; Dorfmeister, M.; Girgis, M.; Alharbi, W.; Chetrit, M.; Galatas, C.; Mamane, S.; Sebag, I.; Buithieu, J.; Bilodeau, L.; de Varennes, B.; Lachapelle, K.; Lange, R.; Martucci, G.; Virmani, R.; Piazza, N.	2015	Heart	99(13)		960-967	Valve intervention
592	Calcified Cerebral Embolism Due to a Calcified Amorphous Tumor	Nagao, Y.; Nakajima, M.; Hirahara, T.; Wada, K.; Terasaki, T.; Nagamine, M.; Ando, Y.	2018	Journal of Stroke & Cerebrovascular Diseases	27	7	e115-e116	Not MAC
593	Long-term follow-up of patients with echocardiographically detected mitral anular calcium and comparison with age- and sex-matched control subjects	Nair, C. K.; Thomson, W.; Ryschon, K.; Cook, C.; Hee, T. T.; Sketch Sr, M. H.	1989	American Journal of Cardiology	63(7)		465-470	Not MAC thrombus
594	New insights into stentless porcine bioprosthesis failure	Nair, V.; Law, K. B.; Phillips, K.; Li, A. Y.; David, T. E.; Butany, J.	2010	Cardiovascular Pathology	21(3)		158-168	Prosthetic valve
595	Characterizing the inflammatory reaction in explanted Medtronic Freestyle stentless porcine aortic bioprosthesis over a 6-year period	Nair, V.; Law, K. B.; Li, A. Y.; Phillips, K. R. B.; David, T. E.; Butany, J.	2012	Laboratory Investigation	1)		83A	Prosthetic valve

596	Calcified amorphous tumor of the heart with mitral annular calcification: a case report	Nakamaru, R.; Oe, H.; Iwakura, K.; Masai, T.; Fujii, K.	2017	Journal of Medical Case Reports [Electronic Resource]	11	1	195	Calcified amorphous tumor, not MAC thrombus
597	Long-term results of open mitral commissurotomy for mitral stenosis with severe subvalvular changes: a ten-year evaluation	Nakano, S.; Kawashima, Y.; Hirose, H.; Matsuda, H.; Shimazaki, Y.; Sato, S.; Ohyama, C.	1984	Annals of Thoracic Surgery	37	2	159-63	Valve intervention
598	A case of cardiac calcified amorphous tumor (cardiac CAT) causing acute embolism in right common iliac artery	Nakashima, Y.; Terauchi, Y.; Noguchi, T.; Tanioka, K.; Kubo, T.; Yamasaki, N.; Kitaoka, H.	2015	Journal of Cardiology Cases	11	3	81-84	Calcified amorphous tumor, not MAC thrombus
599	Caseous Mitral Annular Calcification: An Unrecognized Cause of Cardioembolic Events	Nandar, P. P.; Zulqarnain, M.; Chaffee, R.	2020	Journal of the American College of Cardiology	75(11)		3145	Not MAC thrombus
600	Structural valve degeneration in the transcatheter mitral valve implantation era	Narayan, P.	2021	Journal of Cardiac Surgery	36(11)		4431-4433	Prosthetic valve
601	Emergency craniocerebral hypothermia in the management of open mitral commissurotomy. [Russian]	Naumenko, S. E.	1990	Anesteziologiia i reanimatologiia		3	63-66	Not MAC thrombus
602	Late follow-up of the flexible stent, low profile Liotta bioprosthesis	Navia, J. A.; Belzzitti, J.; Meletti, J.; Liotta, D.	1986	Zeitschrift fur Kardiologie	75 Suppl 2		254-7	Prosthetic valve
603	Integrated 18F-FDG PET/MR imaging in the assessment of cardiac masses: a pilot study	Nensa, F.; Tezgah, E.; Poeppel, T. D.; Jensen, C. J.; Schelhorn, J.; Kohler, J.; Heusch, P.; Bruder, O.;	2015	Journal of Nuclear Medicine	56	2	255-60	Not MAC

		Schlosser, T.; Nassenstein, K.						
604	Mitral annular calcification: clinical, pathophysiology, and echocardiographic review	Nestico, P. F.; Depace, N. L.; Morganroth, J.; Kotler, M. N.; Ross, J.	1984	American Heart Journal	107	5 Pt 1	989-96	No MAC thrombus
605	Mitral valve replacement with posterior transposition of the anterior mitral leaflet which covers and buttresses partially decalcified posterior mitral annular bed	Nezic, D.; Knezevic, A.; Borovic, S.; Jovic, M.	2012	European Journal of Cardio-thoracic Surgery	43(2)		450-451	Not MAC thrombus
606	Reply to Tavlasoglu et al	Nezic, D.; Borovic, S.; Jovic, M.	2013	European Journal of Cardio-Thoracic Surgery	41	5	1129-31	Not MAC thrombus
607	Valve repair in mitral regurgitation complicated by severe annulus calcification	Ng, C. K.; Punzengruber, C.; Pachinger, O.; Nesser, J.; Auer, H.; Franke, H.; Hartl, P.	2000	Annals of Thoracic Surgery	71	1	78-85	Not MAC thrombus
608	Valvuloplasty with glutaraldehyde-treated autologous pericardium in patients with complex mitral valve pathology	Ng, C. K.; Nesser, J.; Punzengruber, C.; Pachinger, O.; Auer, J.; Franke, H.; Hartl, P.	2001	Annals of Thoracic Surgery	70	1	Aug-53	Not MAC thrombus
609	Prosthetic mitral leaflet malfunction due to inappropriate size of implant	Ng, Y. T.; Chung, P. C.; Lau, W. M.	2008	Acta Anaesthesiologica Taiwanica: Official Journal of the Taiwan Society of Anesthesiologists	46	2	Sep-76	Not MAC thrombus
610	Massive cerebral embolism originated from ruptured infective mitral annular calcification in a chronic hemodialysis patient	Nishida, J.; Maeda, T.; Yuda, S.; Tanaka, M.; Hashimoto, A.;	2009	Echocardiography	26	1	107-8	Not MAC thrombus

		Tsuchihashi, K.; Shimamoto, K.						
611	Cardiac abnormalities in ischemic cerebrovascular disease studied by two-dimensional echocardiography	Nishide, M.; Irino, T.; Gotoh, M.; Naka, M.; Tsuji, K.	1983	Stroke	14	4	541-5	No thrombus on MAC
612	Cerebral Embolism Associated with Calcified Amorphous Tumor: A Review of Cerebral Infarction Cases	Nishiguchi, Y.; Matsuyama, H.; Shindo, A.; Matsuura, K.; Niwa, A.; Hirota, Y.; Fukuma, T.; Ito, H.; Kozuka, Y.; Tomimoto, H.	2021	Internal Medicine	60	14	2315-2319	Calcified amorphous tumor, not MAC thrombus
613	Factors affecting natural illnesses during the covid-19 pandemic-autopsy findings in a series of cases	Noone, P. H.; Kausar, A.; Dayananda, R.	2021	Indian Journal of Forensic Medicine and Toxicology	15(2)		467-470	Not MAC thrombus
614	Embolic Stroke	Ntaios, G.; Hart, R. G.	2017	Circulation	136(25)		2403-2405	Not MAC thrombus
615	Update on percutaneous mitral commissurotomy	Nunes, M. C.; Nascimento, B. R.; Lodi-Junqueira, L.; Tan, T. C.; Athayde, G. R.; Hung, J.	2016	Heart	102	7	500-7	Not MAC thrombus
616	The Medtronic Intact xenograft: An analysis of 342 patients over a seven-year follow-up period	O'Brien, M. F.; Stafford, E. C.; Gardner, M. A. H.; Pohlner, P. G.; Tesar, P. J.; Kear, L.; Smith, S. E.	1995	Annals of Thoracic Surgery	60(2 SUPPL.)		S253-S257	Prosthetic valve
617	Usefulness of transesophageal echocardiography in predicting mortality and morbidity in stroke patients without clinically known cardiac sources of embolus	O'Brien, P. J.; Thiemann, D. R.; McNamara, R. L.; Roberts, J. W.; Raska, K.; Oppenheimer, S. M.; Lima, J. A.	1998	American Journal of Cardiology	81	9	1144-51	No thrombus on MAC

618	Long-term echocardiographic follow-up of patients with a tricuspid bioprosthesis	Okada, Y.; Nasu, M.; Nishiuchi, S.; Shomura, T.	1990	ASAIO Transactions	36	3	M535-7	Prosthetic valve
619	Predictors for maintenance of sinus rhythm after cardioversion in patients with nonvalvular atrial fibrillation	Okcun, B.; Yigit, Z.; Kucukoglu, M. S.; Mutlu, H.; Sansoy, V.; Guzelsoy, D.; Uner, S.	2002	Echocardiography	19	5	351-7	Not MAC thrombus
620	Closed valvulotomy for calcific mitral stenosis	Olinger, G. N.; Rio, F. W.; Maloney, J. V., Jr.	1971	Journal of Thoracic & Cardiovascular Surgery	62	3	357-65	Valve intervention
621	Structural deterioration of the cryopreserved mitral homograft valve	Olivito, S.; Lalande, S.; Nappi, F.; Hammoudi, N.; D'Alessandro, C.; Fouret, P.; Acar, C.	2012	Journal of Thoracic and Cardiovascular Surgery	144(2)		313-320	Prosthetic valve
622	Percutaneous mitral valvuloplasty as a treatment of choice for mitral stenosis. Immediate results and long-term follow-up. [Spanish]	Olmos, A.; Gajardo, J.; Lecannelier, E.; Seguel, I.; Lama, A.; Otero, C.; Fasce, E.	1991	Revista medica de Chile	119(1)		27-32	Not MAC thrombus, mitral valve intervention
623	Left atrial thrombus in a patient without mitral valve disease or atrial fibrillation	Omoto, T.; Aoki, A.; Maruta, K.; Masuda, T.; Hirono, M.	2016	Journal of Cardiology Cases	14	4	115-118	No MAC
624	Six years clinical experience with dura mater cardiac valves	Ongcharit, C.; Ongcharit, C. N.; Sanpradit, M.; Kurowat, Y.; Manothaya, C.; Buranatham, C.	1983	Thoracic & Cardiovascular Surgeon	31	5	282-7	Prosthetic valve
625	Results of instrumental transventricular commissurotomy: a follow-up study	Orndahl, G.; Seeman, T.; Linder, E.; Varnauskas, E.	1966	Acta Medica Scandinavica	179	2	129-45	Valve intervention

626	Left atrial free-floating ball thrombus in a patient without mitral valve disease. [Japanese]	Oryoji, A.; Kawara, T.; Hara, H.; Aoyagi, S.; Kosuga, K.; Ohishi, K.	1993	[Zasshi] [Journal]	Nihon Kyobu Geka Gakkai. 41(4)	699-703	No MAC
627	2020 ACC/AHA guideline for the management of patients with valvular heart disease: A report of the American College of Cardiology/American Heart Association Joint Committee on Clinical Practice Guidelines	Otto, C. M.; Nishimura, R. A.; Bonow, R. O.; Carabello, B. A.; Erwin, J. P.; Gentile, F.; Jneid, H.; Krieger, E. V.; Mack, M.; McLeod, C.; O'Gara, P. T.; Rigolin, V. H.; Sundt, T. M.; Thompson, A.; Toly, C.; Beckman, J. A.; Levine, G. N.; Al-Khatib, S. M.; Armbruster, A.; Birtcher, K. K.; Ciggaroa, J.; Deswal, A.; Dixon, D. L.; Fleisher, L. A.; de las Fuentes, L.; Goldberger, Z. D.; Gorenek, B.; Haynes, N.; Hernandez, A. F.; Hlatky, M. A.; Joglar, J. A.; Jones, W. S.; Marine, J. E.; Mark, D.; Palaniappan, L.; Piano, M. R.; Spatz, E. S.; Tamis-Holland, J.; Wijeysundera, D. N.; Woo, Y. J.	2021	Journal of Thoracic and Cardiovascular Surgery	162(2)	e183-e353	Not MAC thrombus

628	New perspectives by imaging modalities for an old illness: Rheumatic mitral stenosis	Oz, T. K.; Tok, O. O.; Sade, L. E.	2020	Anatolian Journal of Cardiology	23(3)		128-140	Rheumatic disease
629	Ring annuloplasty and successful mitral valve repair in a staphylococcal endocarditis case with bilobular saccular mycotic aneurysm at cerebral artery and frontal region infarction. Secondary to septic emboli	Ozbek, C.; Yetkin, U.; Bademci, M.; Karahan, N.; Gurbuz, A.	2008	Archives of Medical Science	4(1)		94-99	Not MAC
630	Effect of fibroblast growth factor-2 on pannus formation in replacement prosthetic heart valves	Ozeren, M.; Demirpence, O.; Han, U.; Dogan, O. V.; Yucel, E.; Onal, B.	2004	Journal of Heart Valve Disease	13	4	676-80	Not MAC prosthetic valve
631	Early and mid-term clinical experience with extracellular matrix scaffold for congenital cardiac and vascular reconstructive surgery: A multicentric Italian study	Padalino, M. A.; Quarti, A.; Angeli, E.; Frigo, A. C.; Vida, V. L.; Pozzi, M.; Gargiulo, G.; Stellin, G.	2015	Interactive Cardiovascular and Thoracic Surgery	21(1)		40-49	Valve repair
632	Mitral valve reconstruction for active and healed endocarditis	Pagani, F. D.; Monaghan, H. L.; Deeb, G. M.; Bolling, S. F.	1996	Circulation	94	9 Suppl	II133-8	Not MAC thrombus
633	Thrombotic Risk and Antithrombotic Strategies After Transcatheter Mitral Valve Replacement	Pagnesi, M.; Moroni, F.; Beneduce, A.; Giannini, F.; Colombo, A.; Weisz, G.; Latib, A.	2019	Jacc: Cardiovascular Interventions	12	23-Jan	2388-2401	Valve intervention
634	One-stop-shop totally percutaneous approach for severe aortic and mitral regurgitation in cardiogenic shock	Pagnotta, P. A.; Sanz-Sanchez, J.; Regazzoli, D.; Ferrante, G.	2020	Catheterization and Cardiovascular Interventions	95(3)		411-413	Not MAC
635	Percutaneous Mitral Balloon Valvuloplasty for Patients with Rheumatic Mitral Stenosis	Palacios, I. F.; Arzamendi, D.	2012	Interventional Cardiology Clinics	1(1)		45-61	Rheumatic disease

636	Mitral and aortic calcifications are associated with cerebrovascular ischemic episodes in coronary patients	Palka, I.; Mostowik, M.; Gackowski, A.; Nessler, J.	2013	European Heart Journal	1)		60	MAC & thromboembolic events but no MAC thrombus
637	Retinal embolization of bicuspid aortic valve calcification	Palmiero, P.; Maiello, M.; Nanda, N. C.	2004	Echocardiography	21	6	541-4	Not MAC thrombus
638	Monocular blindness secondary to calcific embolization. An unusual presentation of rheumatic mitral valvular disease	Pantely, G. A.; Housman, L. B.; DeMots, H.; Rahimtoola, S. H.	1976	Chest	69	4	555-6	Rheumatic disease
639	A case of catastrophic calcific uremic arteriolopathy involving multiple organ systems in the setting of esrd	Papanagnou, A.; Ahmed, W.	2019	Journal of the American Society of Nephrology	30		1113	No proven MAC thrombus
640	[Left atrial massive thrombosis and calcification of the auricular wall as mitral valvotomy complication, Considerations on 17 cases surgically treated (author's transl)]	Pardini, S.; Bovolato, P.; Bonandi, L.; Dottori, V.; Mombelloni, G. T.	1981	Giornale Italiano di Cardiologia	11	10	1477-80	Valve intervention
641	Percutaneous mitral valvuloplasty using the double balloon technique: immediate results and determinant factors of increasing mitral regurgitation	Park, S. J.; Lee, W. K.; Shim, W. H.; Cho, S. Y.; Tahk, S. J.; Kim, S. S.	1991	European Heart Journal Cardiovascular Imaging	2)		ii181	Mitral valve intervention
642	Intrapulmonary shunt in cryptogenic stroke in older Patients: True cause of paradoxical embolism?	Park, D. G.; Hong, J. Y.; Kim, S. E.; Lee, J. H.; Han, K. R.; Oh, D. J.	2011	Korean Journal of Internal Medicine	6	2	Jul-51	Not MAC thrombus
643	Unusual collateral vessel from right subclavian vein to left atrium, a rare complication of superior vena cava obstruction	Parsaee, M.; Pouraliakbar, H.; Ghadrdoost, B.; Moosavi, J.; Behjati, M.	2018	Echocardiography	35	8	1233-1236	Not MAC thrombus
644	An Incidental Finding of a Cardiac Mass	Patel, J.; Sharma, H.; Patel, P.; Patel, C.; Bellary, S.; Al-khateeb, A.;	2019	Chest	156(4 Supplement)		A644-A645	Not MAC thrombus

		Kadri, M.; Miller, R.; Goldfarb, I.						
645	The value of transesophageal echocardiography in predicting immediate and long-term outcome of balloon mitral valvuloplasty: comparison with transthoracic echocardiography	Pavlides, G. S.; Hauser, A. M.; Dudlets, P. I.; Safian, R. D.; O'Neill, W. W.	1994	Journal of Interventional Cardiology	7	5	401-8	Not MAC thrombus
646	Evolution of percutaneous balloon aortic valvuloplasty in the treatment of patients with aortic stenosis	Pendyala, L. K.; Ben-Dor, I.; Waksman, R.	2012	Minerva Medica	103(6)		415-429	Not MAC thrombus
647	Clinical rentability of echocardiography in acute ischemic stroke	Perez Contreras, E.; Migone, G.; Vilades Medel, D.; Li, C.; Leta Petracca, R.; Marti Fabregas, J.; Carreras Costa, F.	2019	European Heart Journal Cardiovascular Imaging	20(Supplement 1)		i613	Not MAC thrombus
648	Medium term fate of dura mater valvular bioprostheses	Permanyer-Miralda, G.; Soler-Soler, J.; Casan-Cava, J. M.; Tornos-Mas, M. P.	1980	European Heart Journal	1	3	195-9	Prosthetic valve
649	Mitral valve re-replacement in a patient with osteogenesis imperfecta	Pfannmueller, B.; Borger, M. A.; Battellini, R. R.; Mohr, F. W.	2010	Thoracic and Cardiovascular Surgeon	58(8)		486-488	Not MAC thrombus
650	Stuyding the Role of 3D Transthoracic Echocardiography For the Evaluation of Rheumatic Mitral Stenosis Compare to 2D Transesophageal Echocardiography	Phan, H. T.	2018	Global Heart	13(4)		390	Rheumatic disease
651	Transcatheter Aortic Valves for Failing Surgical Mitral Prostheses and Mitral Annular Calcification: Good From Far But Far From Good? [*]	Piazza, N.; Pighi, M.; Martucci, G.	2017	JACC: Cardiovascular Interventions	10(19)		1943-1945	Not MAC thrombus

652	Echocardiographic evidence of systemic atherosclerosis and long-term outcomes in patients with non-hemorrhagic stroke	Pieper, J.; Ashamalla, M.; Yager, N.; Sedhom, D.; Ghate, K.; Nguyen, V.; Shkolnik, B.; Torosoff, M.	2015	Arteriosclerosis, Thrombosis, and Vascular Biology. Conference: American Heart Association's Arteriosclerosis, Thrombosis and Vascular Biology/Peripheral Vascular Disease	35	SUPP L. 1		Not MAC thrombus
653	Intracardiac masses. Tumors, vegetations, thrombi, and foreign bodies	Pietro, D. A.; Parisi, A. F.	1980	Medical Clinics of North America	64	2	239-51	Review; no case of MAC thrombus
654	Acute complications following balloon mitral valvuloplasty - Analysis of 1450 cases over 15-year period	Pillai, A. A.; Annapurni, A.; Ramasamy, C.	2015	Journal of the American College of Cardiology	1)		S81	Valve intervention
655	Assessment of Prosthetic Valve Function After TAVR	Pislaru, S. V.; Nkomo, V. T.; Sandhu, G. S.	2016	JACC: Cardiovascular Imaging	9(2)		193-206	Prosthetic valve
656	Severe Mitral Annular Calcification and Mitral Valve Surgery: An Algorithmic Approach to Management	Pizano, A.; Hirji, S. A.; Nguyen, T. C.	2020	Seminars in Thoracic and Cardiovascular Surgery	32(4)		630-634	Not MAC thrombus
657	Marked thrombosis and calcification of porcine heterograft valves	Platt, M. R.; Mills, L. J.; Estrera, A. S.; Hillis, L. D.; Buja, L. M.; Willerson, J. T.	1980	Circulation	62	4	862-9	Prosthetic valve
658	Prominent posterior mitral annular calcification causing embolic stroke and mimicking left atrial fibroma	Poh, K. K.; Wood, M. J.; Cury, R. C.	2007	European Heart Journal	28(18)		2216	No thrombus on MAC
659	Acquired nonrheumatic valvular and endocardial pathology	Pomerance, A.	1977	Pathology Annual	12(2)		151-187	Not MAC thrombus

660	Fisics-Incor bovine pericardial bioprostheses: 15 year results	Pomerantzeff, P. M.; Brandao, C. M.; Cauduro, P.; Puig, L. B.; Grinberg, M.; Tarasoutchi, F.; Cardoso, L. F.; Lerner, A.; Stolf, N. A.; Verginelli, G.; Jatene, A. D.	1998	Heart Surgery Forum	1	2	130-5	Prosthetic valve
661	Giant left atrium with calcified walls and thrombus in a patient with an old, normally functioning ball-in-cage mitral valve prosthesis	Popescu, B. A.; Lupescu, I.; Georgescu, S. A.; Ginghina, C.	2010	Circulation	122	24	e579-80	Prosthetic valve
662	[Surgical correction of the rheumatic concomitant mitral-aortal failure complicated by thrombosis of the left auricle]	Popov, V. V.	2002	Heart Surgery Forum	2)		S71	Rheumatic valve
663	Mitral valve replacement after previous closed mitral commissurotomy: Problems solved and unsolved	Popov, V. V.; Knyshov, G. V.; Trombovetskaya, O. M.	2010	Heart Surgery Forum	2)		S111	Valve intervention
664	Open mitral commissurotomy in surgery of isolated mitral valve disease: Problems solved and unsolved	Popov, V. V.; Knyshov, G. V.; Trombovetskaya, O. M.; Mnishenko, V. I.	2010	Klinicheskaia Khirurgiia		3	11-Sep	Not MAC thrombus
665	Patients-prosthesis mismatch after mitral valve replacement with small cavity of left ventricle	Popov, V. V.; Knyshov, G. V.; Trombovetskaya, O. M.; Zakharova, V. P.	2010	Interactive Cardiovascular and Thoracic Surgery	1)		S112	Mitral valve prosthesis
666	Associated risk-factors of high pulmonary hypertension for isolated mitral valve replacement	Popov, V. V.; Knyshov, G. V.; Trombovetskaya, O. M.; Zakharova, V. P.	2010	Interactive Cardiovascular and Thoracic Surgery	1)		S111	Not MAC thrombus
667	Combined mitral-aortic valve diseases: Risk-factors of surgery	Popov, V.; Knyshov, G.; Trembovetskaya,	2011	Interactive Cardiovascular	1)		S98-S99	Valve intervention

		O.; Beshlyaga, V.; Rybakova, O.		and Thoracic Surgery				
668	Surgical treatment of isolated mitral valve disease complicated with massive thromboses of left atrium	Popov, V.; Sytar, L.; Mnishenko, V.; Knyshov, G.	2011	Interactive Cardiovascular and Thoracic Surgery	1)		S24	No thrombus on MAC
669	[Surgical treatment of isolated mitral valve stenosis, complicated by massive thrombosis of left atrium]	Popov, V. V.; Mnishenko, V. I.	2014	Klinicheskaia Khirurgiia		7	May-34	Not MAC thrombus
670	Learning from the pulmonary veins	Porres, D. V.; Morenza, O. P.; Pallisa, E.; Roque, A.; Andreu, J.; Martinez, M.	2013	Radiographics	33	4	999-1022	Not MAC thrombus
671	Mitral annular calcification is an independent predictor of unfavourable short-term outcome of acute ischemic stroke	Potpara, T. S.; Djikic, D.; Polovina, M.; Marcetic, Z.; Peric, V.; Lip, G. Y. H.	2013	European Heart Journal Cardiovascular Imaging	2)		ii119	Not MAC thrombus
672	Diagnosis of a calcified intramural mass next to the posterior mitral valve: Review of 4 cases. [French]	Pouliquen, G.; Fresse-Warin, K.; Crochet, D.	2009	Journal de Radiologie	90(5 C1)		597-604	Not MAC thrombus
673	Calcification of the mitral annulus and its complications	Pounder, D. J.	1982	American Journal of Forensic Medicine & Pathology	3	2	109-13	Publication before 1985
674	Calcium embolism of the coronary arteries after percutaneous mitral balloon valvuloplasty	Powell, B. D.; Holmes, D. R., Jr.; Nishimura, R. A.; Rihal, C. S.	2001	Mayo Clinic Proceedings	76	7	753-7	Valve intervention
675	Caseous Calcification of the Mitral Annulus	Pradella, S.; Verna, S.; Addeo, G.; Oddo, A.; Miele, V.	2019	Journal of Radiology Case Reports	13	1	Jan-23	Not MAC thrombus

676	Simultaneous endovascular aneurysm repair and transcatheter aortic valve implantation in a patient with severe aortic stenosis and abdominal aortic aneurysm	Prandi, F. R.; Idone, G.; De Nardo, D.; Cammalleri, V.; Muscoli, S.; Romeo, F.	2019	European Heart Journal, Supplement	21(SUPPL J)		J208	No MAC thrombus
677	Contemporary criteria for the selection of patients for percutaneous balloon mitral valvuloplasty	Prendergast, B. D.; Shaw, T. R. D.; Iung, B.; Vahanian, A.; Northridge, D. B.	2002	Heart	87(5)		401-404	Not MAC thrombus
678	Computed Tomography as a Superior Diagnostic Test for Left Atrial Thrombus With Calcification	Pu, L.; Zhang, Y.; Liu, Y.; Liu, B.; Yang, Y.; Li, Y.	2019	Annals of Thoracic Surgery	108	3	e175-e177	Rheumatic disease
679	Images in cardiovascular medicine: caseous calcification of the mitral annulus	Pugliatti, P.; Piccione, M. C.; Ascenti, G.; Germano, A.; Recupero, A.; Oreto, G.; Carerj, S.	2013	Echocardiography	30	2	E30-2	Not MAC thrombus
680	Dura mater mitral and tricuspid bioprostheses: 30 years of follow-up	Puig, L. B.; Brandao, C. M.; Kawabe, L.; Verginelli, G.; Ramires, J. A.; de Oliveira, S. A.	2003	Revista do Hospital das Clinicas; Faculdade de Medicina Da Universidade de Sao Paulo	58	3	163-8	Prosthetic valve
681	Mitral annular calcification as a marker of complex aortic atheroma in patients with stroke of uncertain etiology	Pujadas, R.; Arboix, A.; Anguera, N.; Rafel, J.; Sagues, F.; Casanas, R.	2008	International Journal of Cardiology	95	03-Feb	129-34	No MAC thrombus
682	Specific cardiac disorders in 402 consecutive patients with ischaemic cardioembolic stroke	Pujadas Capmany, R.; Arboix, A.; Casanas-Munoz, R.; Anguera-Ferrando, N.	2004	Echocardiography	25	2	124-32	No MAC thrombus
683	[Prevalence and clinical significance of incidental cardiac	Quentin, M.; Kropil, P.; Steiner,	2011	Der Radiologe	51(1)		59-64	Not MAC thrombus

	findings in non-ECG-gated chest CT scans]. [German]	S.; Lanzman, R. S.; Blondin, D.; Miese, F.; Choy, G.; Abbara, S.; Scherer, A.						
684	Mitral annulus calcification and sudden death	Quick, E.; Byard, R. W.	2013	Journal of Forensic & Legal Medicine	20	4	204-6	No MAC thrombus
685	The relationship between the thrombotic and infectious complications of central venous catheters	Raad,, II; Luna, M.; Khalil, S. A.; Costerton, J. W.; Lam, C.; Bodey, G. P.	1994	JAMA	271	13	1014-6	Not MAC thrombus
686	Factors influencing thromboembolism after mitral prosthetic implants	Rabago, G.; Fraile, J.; Martinell, J.; Artiz, V.; Cortina, J.	1986	Zeitschrift fur Kardiologie	75 Suppl 2		341-4	Prosthetic valve
687	Mitral annulus calcification: Potential indicator of vascular disease and higher thromboembolic risk	Radu, S.; Al Shurbaji, S.; Mircea, G.; Ouatu, A.; Tanase, D. M.; Floria, M.	2020	European Heart Journal Cardiovascular Imaging	21(Supplement 1)		i27	Not MAC thrombus
688	Double valve replacement: A heroic and unique life-saving procedure in a lung-kidney transplant recipient; a case report	Rafiroiu, S.; Latifi, M.; Budev, M.; Tong, M. Z. Y.; Pettersson, G.; Johnston, D.	2017	Chest	152(4 Supplement 1)		A31	Not MAC thrombus
689	Acquired submitral aneurysms may be associated with mitral paravalvular leaks	Raghuram, P.; Sivakumar, K.; Sheriff, E. A.	2021	Asian Cardiovascular and Thoracic Annals	29(8)		822-825	Valve intervention not MAC
690	The Year in Valvular Heart Disease	Rahimtoola, S. H.	2004	Journal of the American College of Cardiology	43(3)		491-504	Review; not case of MAC thrombus
691	The year in valvular heart disease	Rahimtoola, S. H.	2005	Journal of the American College of Cardiology	45(1)		111-122	Review; not case of MAC thrombus

692	The year in valvular heart disease	Rahimtoola, S. H.	2014	Journal of the American College of Cardiology	63(19)		1948-1958	Review; not case of MAC thrombus
693	Immediate echocardiographic and hemodynamic outcome of percutaneous transvenous mitral commissurotomy in elderly bangladeshi severe symptomatic mitral stenosis patients	Rahman, T.	2016	BMJ Case Reports	2017 (no pagination)			Rheumatic valve
694	Mitral valve prosthesis implanted in the atrial position in a patient with extensive calcification extending from epicardium to mitral annulus	Rahman, S. G.; Rehman, A.	2017	Catheterization and Cardiovascular Interventions	2)		S152	Not thrombus on MAC
695	Ptmc in elderly: A challenging case with complications	Rahman, T.; Rahman, A.	2018	Journal of the American College of Cardiology	71(16 Supplement 1)		S286-S287	Not MAC thrombus
696	Atorvastatin attenuates aortic valve disease via a tissue stem cell niche	Rajamannan, N.	2010	Arteriosclerosis , Thrombosis and Vascular Biology	29(7)		e114	Not MAC thrombus
697	Myocardial infarction due to embolism of the right coronary ostium from chronic rheumatic mitral valvular disease	Rajasekhar, A.; Swaroop, B. N.; Raju, B. S.	1996	Indian Heart Journal	48	2	165-8	Rheumatic valve
698	Aortic Valve Replacement	Rajput, F. A.; Zeltser, R.	2020	StatPearls Publishing	1		1	Not MAC thrombus
699	Calcific emboli in infective endocarditis	Ramachandran, P.; Drogemuller, G.; Burrow, J.	2017	Journal of Clinical Neuroscience	42		223-224	Not MAC thrombus
700	Calcific retinal embolism as an indicator of severe unrecognised cardiovascular disease	Ramakrishna, G.; Malouf, J. F.; Younge, B. R.; Connolly, H. M.; Miller Jr, F. A.	2005	Heart	91(9)		1154-1157	Not MAC thrombus

701	The pivotal role of echocardiography in cardiac sources of embolism	Rana, B. S.; Monaghan, M. J.; Ring, L.; Shapiro, L. S.; Nihoyannopoulos, P.	2011	European Journal of Echocardiography	12(10)		i25-i31	Not MAC thrombus
702	Reassessment of usefulness of porcine heterografts in mitral position in children	Rao, P. S.; Solymar, L.; Fawzy, M. E.; Guinn, G.	1991	Pediatric Cardiology	12(3)		164-169	Prosthetic valve
703	Frequency of mitral annular calcification in patients on hemodialysis estimated by 2-dimensional echocardiography	Raos, V.; Jeren-Strujic, B.; Antos, M.; Horvatin-Godler, S.	1996	Acta Medica Croatica	50	05-Apr	179-83	Not MAC thrombus
704	Double-Chamber Left Atrium After Mitral Valve Surgery	Raut, M. S.; Hanjoora, V. M.; Chishti, M. A.; Sharma, A.	2020	Journal of Cardiothoracic and Vascular Anesthesia	34(1)		297-298	Not MAC thrombus
705	Retinal artery embolization: A rare presentation of calcific aortic stenosis	Reimers, C. D.; Williams, R. J.; Berger, M.; Wisnicki, H. J.; Tranbaugh, R. F.	1996	Clinical Cardiology	19(3)		253-254	Not MAC thrombus
706	The third generation Carpentier-Edwards bioprosthesis: early results	Relland, J.; Perier, P.; Lecointe, B.	1985	Journal of the American College of Cardiology	6	5	1149-54	Prosthetic valve
707	New Ventricular Septal Defects Following Balloon-Expandable Transcatheter Aortic Valve Replacement	Rene, A. G.; Jagasia, D.; Wickramasinghe, S. R.; Desai, N.; Szeto, W.; Vallabhajosyula, P.; Li, R. H.; Silvestry, F. E.; Giri, J.; Jha, S.; Herrmann, H. C.; Anwaruddin, S.	2016	Journal of Invasive Cardiology	28(7)		E59-E65	Not MAC thrombus
708	Circulatory arrest due to retrograde embolization of a transapically implanted aortic	Reske, A.; Ibrahim, K.;	2016	A and A Case Reports	6(7)		193-195	Not MAC thrombus

	valve prosthesis with subsequent inversion and left ventricular outflow occlusion	Reske, A. W.; Kappert, U.						
709	Liquefactive necrosis of the mitral ring	Restrepo, G.; Ahumada, S.; Trespalacios, E.	2013	Revista Colombiana de Cardiologia	20(5)		320-324	Not MAC thrombus
710	Valve failure with the Ionescu-Shiley bovine pericardial bioprosthesis: Analysis of 2680 patients	Reul Jr, G. J.; Cooley, D. A.; Duncan, J. M.	1985	Journal of Vascular Surgery	2(1)		192-204	Prosthetic valve
711	Results of aortic valve replacement with the supra-annular Sorin Bicarbon Overline prosthesis	Reyes, G.; Badia, S.; Alvarez, P.; Kallmeyer, C.; Rodriguez, S.; Sarraj, A.; Bustamante, J.	2012	Journal of Heart Valve Disease	21(3)		358-363	Not MAC thrombus
712	Reoperation for Mitral Stenosis	Richardson, J. P.; Sutherland, H. D.	1964	Medical Journal of Australia	1	8	367-71	Previous valve intervention
713	Mitral valve replacement with heterologous aortic valves	Richardson, J. P.; Clarebrough, J. K.; Simpson, W. L.	1969	Medical Journal of Australia	1		942-4	Not MAC thrombus
714	[Echocardiographic discoveries in 102 patients with vascular cerebral accidents]	Ricou, F.; Gabathuler, J.; Aebischer, N.; Rohr, J.; Lerch, R.; Rutishauser, W.	1987	Archives des Maladies du Coeur et des Vaisseaux	80	7	1151-7	Not MAC thrombus
715	Spontaneous calcific emboli from calcific mitral annulus fibrosus	Ridolfi, R. L.; Hutchins, G. M.	1976	Archives of Pathology & Laboratory Medicine	100	3	117-20	Publication before 1985
716	Role of echocardiography in mitral commissurotomy with the Inoue balloon	Roberts, J. W.; Lima, J. A.	1994	Catheterization & Cardiovascular Diagnosis	Suppl 2		69-75	Not MAC thrombus

717	Complications of Radiofrequency Ablation for Supraventricular Tachycardia in the Wolff-Parkinson-White Syndrome Associated With Noncompaction Cardiomyopathy	Roberts, W. C.; Grayburn, P. A.; Hall, S. A.	2018	American Journal of Cardiology	121	11	1442-1444	Not MAC thrombus
718	Emboligenic calcifications on the posteromedial mitral valve chordae. [French]	Rochiccioli, J. P.; Rodriguez, F.; Mordant, B.; Dalous, P.; Glock, Y.; Puel, P.; Bernadet, P.	1988	Archives des Maladies du Coeur et des Vaisseaux	81(6)		787-789	Not MAC thrombus
719	Open mitral valvulotomy	Roe, B. B.; Edmunds, L. H., Jr.; Fishman, N. H.; Hutchinson, J. C.	1971	Annals of Thoracic Surgery	12	5	483-91	Not MAC thrombus
720	Potential cardioembolic sources in an elderly population without stroke. A transthoracic and transoesophageal echocardiographic study in randomly selected volunteers	Roijer, A.; Lindgren, A.; Rudling, O.; Wallin, L.; Olsson, S. B.; Johansson, B. B.; Eskilsson, J.	1996	Scandinavian Cardiovascular Journal	31	6	329-37	Not MAC thrombus
721	Cardiac changes in stroke patients and controls evaluated with transoesophageal echocardiography	Roijer, A.; Lindgren, A.; Algotsson, L.; Norrving, B.; Olsson, B.; Eskilsson, J.	1997	European Heart Journal	17	7	1103-11	Not MAC thrombus
722	Valvular and coronary heart disease in systemic inflammatory diseases	Roldan, C. A.	2008	Heart	94(8)		1089-1101	Not MAC thrombus
723	Aortic Atherosclerosis in Systemic Lupus Erythematosus	Roldan, P. C.; Ratliff, M.; Snider, R.; Macias, L.; Rodriguez, R.; Sibbitt, W.; Roldan, C. A.	2014	Rheumatology. Suppl	5			Not MAC thrombus

724	[Results of closed mitral commissurotomy after 20-25 years]	Romanov, E. I.; Karov, V. V.	1992	Kardiologiia	32	6	Jun-44	Valve intervention
725	Caseous calcification of the mitral annulus-A clinical case	Rosario, M.; Braz, A. L.; Quadrado, C.	2013	European Journal of Internal Medicine	1)		e21	Not MAC thrombus
726	Pathology of the formalin-treated heterograft porcine aortic valve in the mitral position	Rose, A. G.	1972	Thorax	27	4	401-9	Not MAC thrombus
727	Valvular heart disease. The national society journals present selected research that has driven recent advances in clinical cardiology	Rosenhek, R.	2012	Archivos de Cardiologia de Mexico	82(2)		181-194	Not MAC thrombus
728	Melas syndrome presenting with acute congestive heart failure: a rare genetic cause of dilated-hypertrophic cardiomyopathy	Rossi, M.; Luppi, M.; Damiani, G.; Matteo, F.; De Ponti, R.	2019	European Heart Journal, Supplement	21(SUPPL J)		J131-J132	Not MAC thrombus
729	Relation between mitral annular calcium and complex intraaortic debris	Rubin, D. C.; Hawke, M. W.; Plotnick, G. D.	1993	American Journal of Cardiology	71	13	1251-2	Not MAC thrombus
730	Endothelin receptor antagonists for the treatment of pulmonary artery hypertension	Rubin, L. J.	2012	Life Sciences	91(13-14)		517-521	Not MAC thrombus
731	Too Early to Fail, Too Sick to Replace. Early Bioprosthetic Valve Failure Treated with a Valve in Valve Transcatheter Procedure	Ruiz, C. R.; Price, M.; Mohan, R.; Romero, S.; Wesbey, G.	2021	Journal of the American College of Cardiology	77(18 Supplement 1)		2385	Prosthetic valve
732	Caseous calcification of the mitral annulus (Ccma) as potential cardioembolic source: Should anticoagulation be used	Ruiz Escribano Menchen, L.; Corrales Arroyo, M. J.; Franco Salinas, A.; Villanueva Ruiz, F.; Quiros Illan, L.; Gallardo Alcaniz, M. J.; Del Real	2020	International Journal of Stroke	15(1 SUPPL)		508	Not MAC thrombus

		Francia, M. D. L. A.						
733	"Cord-like" mobile left atrial mass related to a caseous calcification of the mitral annulus	Rusch, A.; Boehnel, C.; Weber, A.; Sebastian, L.; Rickli, H.; Buser, M.	2020	Kardiovaskulare Medizin	23(SUPPL 28)		61S-62S	Not MAC thrombus
734	Mitral annulus calcification and cerebral ischaemia	Russell, J. G.	1979	Lancet	2	8149	962	Not MAC thrombus
735	"Patch-glue" annular reconstruction for mitral valve replacement in severely calcified mitral annulus	Ruvolo, G.; Speziale, G.; Voci, P.; Marino, B.	1997	Annals of Thoracic Surgery	63	2	570-1	Not MAC thrombus
736	Characterization of cardiac masses with T1 mapping	Saba, S. G.; Bandettini, P. W.; Shanbhag, S. M.; Spottiswoode, B. S.; Kellman, P.; Arai, A. E.	2015	Journal of Cardiovascular Magnetic Resonance. Conference: 18th Annual SCMR Scientific Sessions. Nice France. Conference Publication:	17	SUPPL. 1		Not MAC thrombus
737	The impact of commissural morphology on clinical outcome in patients undergoing percutaneous balloon mitral valvuloplasty	Sadaka, M. A.; Elsharkawy, E. M.; Ali, R. A.; Rashwan, M. A.	2012	Egyptian Heart Journal	64(4)		233-240	Not MAC thrombus
738	Caseous calcification of the mitral annulus associated with stroke: Report of two cases	Sagnier, S.; Poli, M.; Oysel-Mestre, M.; Corneloup, O.; Debruxelles, S.; Renou, P.; Rouanet, F.; Sibon, I.	2015	Revue Neurologique	171(2)		157-160	Not MAC thrombus
739	Central nervous system damage following surgery using cardiopulmonary bypass--a	Sakakibara, Y.; Shiihara, H.; Terada, Y.; Ino, T.;	1991	Japanese Journal of Surgery	21	1	25-31	Not MAC thrombus

	retrospective analysis of 1386 cases	Wanibuchi, Y.; Furuta, S.						
740	Antithrombotic therapy in valvular heart disease	Salem, D. N.; Levine, H. J.; Pauker, S. G.; Eckman, M. H.; Daudelin, D. H.	1998	Chest	119(1 SUPPL.)		207S-219S	Review; not case of MAC thrombus
741	Antithrombotic therapy in valvular heart disease	Salem, D. N.; Daudelin, D. H.; Levine, H. J.; Pauker, S. G.; Eckman, M. H.; Riff, J.	2001	Chest	114	5 Suppl	590S-601S	Review; not case of MAC thrombus
742	Valvular and structural heart disease: American College of Chest Physicians Evidence-Based Clinical Practice Guidelines (8th Edition)	Salem, D. N.; O'Gara, P. T.; Madias, C.; Pauker, S. G.	2008	Chest	133	6 Suppl	593S-629S	Not MAC thrombus
743	Mitral valve replacement in severely calcified mitral valve annulus: a 10-year experience	Salhiyyah, K.; Kattach, H.; Ashoub, A.; Patrick, D.; Miskolczi, S.; Tsang, G.; Ohri, S. K.; Barlow, C. W.; Velissaris, T.; Livesey, S.	2017	European Journal of Cardio-Thoracic Surgery	52	3	440-444	Not MAC thrombus
744	Multi-modal treatment of calciphylaxis with sodium-thiosulfate, cinacalcet and sevelamer including long-term data	Salmhofer, H.; Franzen, M.; Hitzl, W.; Koller, J.; Kreymann, B.; Fend, F.; Hauser-Kronberger, C.; Heemann, U.; Berr, F.; Schmaderer, C.	2013	Kidney and Blood Pressure Research	37(4-5)		346-359	Not MAC thrombus
745	A 20-year experience with mitral valve repair with artificial chordae in 608 patients	Salvador, L.; Mirone, S.; Bianchini, R.; Regesta, T.; Patelli, F.; Minniti, G.; Masat, M.;	2008	Journal of Thoracic & Cardiovascular Surgery	135	6	1280-7	Valve intervention

		Cavarretta, E.; Valfre, C.						
746	Mitral valve replacement in the presence of massive calcification	Sampathkumar, A.	2013	Journal of Thoracic and Cardiovascular Surgery	146(5)		1300-1301	No thrombus on MAC
747	Guidelines for the Use of Echocardiography in the Evaluation of a Cardiac Source of Embolism	Saric, M.; Armour, A. C.; Arnaout, M. S.; Chaudhry, F. A.; Grimm, R. A.; Kronzon, I.; Landeck, B. F.; Maganti, K.; Michelena, H. I.; Tolstrup, K.	2016	Journal of the American Society of Echocardiography	29(1)		1-42	Not MAC thrombus
748	Calcified apical cardiomyopathy: A rare form of endomyocardial fibrosis	Sbrana, F.; Coceani, M.; Masci, P. G.; Pasanisi, E. M.; Rovai, D.	2015	Journal of Cardiovascular Medicine	16(Supplement 2)		S79-S80	Not MAC thrombus
749	SFAAT: the study of nonrheumatic chronic atrial fibrillation in the Trieste area. Results of an enrollment study. [Italian]	Scardi, S.; Mazzone, C.; Goldstein, D.; Pandullo, C.; Poletti, A.; Humar, F.; Pivotti, F.; De Santis, C.	1995	Giornale italiano di cardiologia	25(2)		173-182	Not MAC thrombus
750	[Stratification of the thromboembolic risk in patients with non-rheumatic atrial fibrillation: assessment of left atrial dysfunction]	Scardi, S.; Pandullo, C.; Mazzone, C.; Goldstein, D.; Zecchin, M.	1996	Giornale Italiano di Cardiologia	26	3	273-85	Not MAC thrombus
751	Device's dehiscence 3 months after implant for mitral perivalvular leak	Scatteia, A.; Losi, M. A.; Parrella, L. S.; Rapacciuolo, A.; Puglia, R.; Prastaro, M.; Buonauro, A.; Esposito, F.; Betocchi, S.	2012	Gazzetta Medica Italiana Archivio per le Scienze Mediche	171(3)		373-376	Prosthetic valve

752	Aortic valve repair versus pericardial valve replacement	Schaff, H. V.; Block, P. C.	2006	ACC Cardiosource Review Journal	15(10)		14-Nov	Not MAC thrombus
753	[Transesophageal, anatomic and Doppler echocardiography. Technic, indications, interpretation]	Scheuble, C.; Castillo-Fenoy, A.	1989	Annales de Cardiologie et d Angeiologie	38	7 Pt 2	463-76	Not MAC thrombus
754	Histology of debris captured by a cerebral protection system during transcatheter valve-in-valve implantation	Schmidt, T.; Schluter, M.; Alessandrini, H.; Akdag, O.; Schewel, D.; Schewel, J.; Thielsen, T.; Kreidel, F.; Bader, R.; Romero, M.; Ladich, E.; Virmani, R.; Schafer, U.; Kuck, K. H.; Frerker, C.	2016	Heart	102	19	1573-80	Prosthetic valve
755	Comparing the diagnostic value of Echocardiography In Stroke (CEIS) - results of a prospective observatory cohort study	Schnieder, M.; Chebbok, M.; Didie, M.; Wolf, F.; Badr, M.; Allam, I.; Bahr, M.; Hasenfuss, G.; Liman, J.; Schroeter, M. R.	2021	BMC Neurology	21(1) (no pagination)			Not MAC thrombus
756	Long-term failure rate and morphologic correlations in porcine bioprosthetic heart valves	Schoen, F. J.; Collins, J. J., Jr.; Cohn, L. H.	1983	American Journal of Cardiology	51	6	957-64	Prosthetic valve
757	Point-of-care Ultrasound in the Evaluation of Mitral Valve Regurgitation and Mitral Annular Calcification	Schoenberg, B.; Alkhattabi, M.; Lahham, S.	2020	Clinical Practice & Cases in Emergency Medicine	4	4	628-629	No thrombus on MAC;
758	Endomyocardial nodular calcification as a cause of heart failure	Segura, A. M.; Radovancevic, R.; Connelly, J. H.; Loyalka, P.;	2011	Cardiovascular Pathology	20(5)		e185-e188	Not MAC thrombus

		Gregoric, I. D.; Buja, L. M.						
759	Right Ventricular Outflow Tract Reconstruction With the Bovine Jugular Vein Graft: 5 Years' Experience With 133 Patients	Sekarski, N.; van Meir, H.; Rijlaarsdam, M. E. B.; Schoof, P. H.; Koolbergen, D. R.; Hruda, J.; von Segesser, L. K.; Meijboom, E. J.; Hazekamp, M. G.	2007	Annals of Thoracic Surgery	84(2)		599-605	Prosthetic valve
760	A complicated case of giant left main coronary artery aneurysm: Apical diverticular aneurysm	Sekuri, C.; Tumuklu, M.; Yuksel, A.; Danaotlu, Z.; Satcan, A.	2014	American Journal of Cardiology	1)		S120	Not MAC thrombus
761	Direct ultrasound-guided puncture of vertebral artery V2 segment during mechanical thrombectomy	Semeraro, V.; Gasparrini, F.; Vidali, S.; Gandini, R.	2021	BMJ Case Reports	14(1) (no pagination)			Not MAC thrombus
762	Management of a Mobile Floating Carotid Plaque Responsible for Calcified Cerebral Emboli: A Double Sword of Damocles	Senemaud, J.; Bounkong, G.; Seddik, L.; Jaziri, A.; Touma, J.	2020	EJVES Vascular Forum	47		69-72	Not MAC thrombus
763	Relationship between mitral annular calcification and severity of carotid atherosclerosis in patients with symptomatic ischemic cerebrovascular disease	Seo, Y.; Ishimitsu, T.; Ishizu, T.; Sakane, M.; Maeda, H.; Fujita, K.; Kamezaki, T.; Watanabe, S.; Yamaguchi, I.	2005	Asian Cardiovascular & Thoracic Annals	24	5	461-3	Not MAC thrombus
764	Cardiac calcified amorphous tumor in a hemodialysis patient	Seo, H.; Fujii, H.; Aoyama, T.; Sasako, Y.	2016	Journal of Cardiology	46(1)		17-24	No thrombus on MAC
765	Calcific mitral stenosis in the hemodialysis patient	Sequeira, A.; Morris, L.; Patel, B.; Duvall, L.; Gali, D.; Menendez, D.; Alexander, G.	2014	Hemodialysis International	18	1	212-4	No thrombus on MAC

766	Particularities of pathological changes in placenta in patients with rheumatic mitral valvulopathy	Serbenco, A.; Sinitsina, L.; Stepan, B.	2013	Journal of Perinatal Medicine. Conference: 11th World Congress of Perinatal Medicine	41	SUPPL. 1		Not MAC thrombus
767	Cardiac calcifications: Beyond the coronaries	Sethi, V.; Dennie, C.; Penna, E.; Peterson, R.	2012	Journal of Thoracic Imaging	27(3)		W76	Not MAC thrombus
768	Mitral valve repair or replacement in elderly people	Sfeir, P. M.; Jebara, V. A.; Ayoub, C. M.	2006	Current Opinion in Anaesthesiology	19(1)		82-87	Not MAC thrombus
769	Left brachial artery: One more way to percutaneous insertion of IMPELLA 2.5L circulatory support for high-risk percutaneous coronary intervention - A case report	Sganzerla, P.; Cinelli, F.; Capoferri, A.; Rondi, M.	2020	European Heart Journal - Case Reports	4(6) (no pagination)			Not MAC thrombus
770	Transcatheter AVR - Does this technology have merit and in whom? An anesthesiologist's perspective	Shanewise, J.	2013	Applied Cardiopulmonary Pathophysiology. Conference: 28th Annual Meeting of the European Association of Cardiothoracic Anaesthesiologists, EACTA	17	2	22-26	Not MAC thrombus
771	Aortic valve repair for aortic stenosis in adults	Shapira, N.; Lemole, G. M.; Fernandez, J.; Daily, P. O.; Dembitsky, W. P.; O'Yek, V.; Haghighi, P.; Stewart, J.; Marsh, D. G.; Bloor, C. M.	1990	Annals of Thoracic Surgery	50	1	110-20	Not MAC thrombus

772	Gastric calciphylaxis in a patient with a functioning renal allograft	Shapiro, C.; Coco, M.	2007	Clinical Nephrology	67(2)		119-125	Not MAC thrombus
773	Transthoracic echocardiographic findings in patients with acute retinal arterial obstruction: A retrospective review	Sharma, S.; Naqvi, A.; Sharma, S. M.; Cruess, A. F.; Brown, G. C.	1996	Journal of the American College of Cardiology	75(11)		3153	Not MAC thrombus
774	Secondary pulmonary arterial hypertension: Treated with endothelin receptor blockade	Sharma, S.; Kashour, T.; Philipp, R.	2005	Journal of the American College of Cardiology	73(9 Supplement 1)		1608	Not MAC thrombus
775	Prevalence and Predictors of Cardiac Pathology on Transthoracic Echocardiography among Patients Admitted for Acute Ischemic Stroke	Sharma, R.; Schwamm, L.; Sanborn, D.	2019	Texas Heart Institute Journal	32(3)		405-410	Not MAC thrombus
776	The Mysterious Mitral Valve Mass: A Case of Valvular Myxoma	Sharma, A.; Kumar, M.; Ricci, A.; Silverman, D.	2020	Archives of Ophthalmology	114(10)		1189-1192	Not MAC thrombus
777	Calcified left atrial myxoma with floppy mitral valve	Sharratt, G. P.; Grover, M. L.; Monro, J. L.	1979	British Heart Journal	42	5	608-10	Not MAC thrombus
778	X-ray appearance and clinical significance of left atrial wall calcification	Shaw, D. R.; Chen, J. T.; Lester, R. G.	1976	Investigative Radiology	11	6	501-7	Not MAC thrombus
779	A case of a mitral valve blood cyst	Shehata, M. S.; Abd El Gawad, H. S.; Abayazeed, R. A.; Elsharkawy, E.; Abd El Hay, M. A.	2019	European Heart Journal Cardiovascular Imaging	20(Supplement 1)		i291	Not MAC thrombus
780	Aortic valve replacement with bovine pericardium in patients with aortic valve regurgitation a single-center experience	Sheng, W.; Zhao, G.; Chao, Y.; Sun, F.; Jiao, Z.; Liu, P.; Zhang, H.; Yao, X.; Lu, F.; Zhan, H.; Zhou, J.; Song, T.; Tao, L.; Liu, C.	2019	International Heart Journal	60(6)		1344-1349	Not MAC thrombus

781	Cerebral embolism	Sherman, D. G.; Dyken, M. L.; Fisher, M.; Harrison, M. J.; Hart, R. G.	1986	Chest	89	2 Suppl	82S-98S	Not MAC thrombus
782	Closed mitral commissurotomy in the surgical treatment of complicated mitral stenosis. [Russian]	Shevchenko, I. L.; Shikhverdiev, N. N.	1994	Vestnik khirurgii imeni I	I. Grekova. 152(1-2)		10-Jun	Not MAC thrombus
783	Diagnosis and surgical treatment of mitral valvular disease with left atrial thrombus (Japanese). [Japanese]	Shimizu, T.; Iyomasa, Y.; Abe, T.	1974	Journal of the Japanese Association for Thoracic Surgery	22(12)		1147-1157	Publication before 1985
784	Significance of echocardiographic evaluation for transcatheter aortic valve implantation	Shirakawa, K.; Murata, M.	2020	Cardiovascular Intervention and Therapeutics	35(1)		85-95	Not MAC thrombus
785	Added value of 3D echo in diagnosing and monitoring transcatheter valve in mac procedure in a patient with severe mitral stenosis	Siciliano, A.; Albuquerque, F. N.; Albuquerque, D. C.; Brito Junior, F. S.; Felix, A. S.; Iso, M. A.; Garcia, R. R.; Mansur Filho, J.; Alcantara, M. L.	2020	European Heart Journal Cardiovascular Imaging	21(Supplement 1)		i116	Not MAC thrombus
786	Calciphylaxis of mitral valve with infective endocarditis	Sidhu, R.; Joolhar, F.; Win, T.; Talai-Shahir, M.; Heidari, A.	2019	Journal of Investigative Medicine	67(1)		142	Not MAC thrombus
787	A second look at autopericardial mitral annuloplasty	Sidiki, A. I.; Faybushevich, A. G.; Lishchuk, A. N.	2020	Cor et Vasa	62(1)		37-43	Valve intervention
788	Echocardiographic examination of the posterior atrioventricular groove	Silbiger, J. J.	2014	Echocardiography	31	2	223-33	No MAC thrombus
789	Advances in Rheumatic Mitral Stenosis: Echocardiographic,	Silbiger, J. J.	2021	Journal of the American Society of	34(7)		709-722.e1	Rheumatic valve

	Pathophysiologic, and Hemodynamic Considerations			Echocardiography				
790	The significance of mitral defect in embolus development in the peripheral arterial system (Slovak). [Slovak]	Simkovic, I.; Vanzurova, E.; Hubka, M.	1975	Bratislavske Lekarske Listy	63(4)		389-394	Not MAC thrombus
791	Resolution of atrial thrombus with adjusted dose warfarin therapy in patient with nonvalvular atrial fibrillation	Simsek, H.; Dogan, A.; Sahin, M.; Gumrukcuoglu, H. A.; Akdag, S.	2011	International Journal of Cardiology	2)		S132	Not MAC thrombus
792	Combined rendezvous approach with the Direct Flow Medical aortic valve prosthesis to treat aortic and mitral stenosis	Sinning, C.; Conradi, L.; Deuschl, F. G.; Schofer, N.; Hakmi, S.; Schafer, U.	2016	International Journal of Cardiology	214		284-285	Not MAC thrombus
793	Closed mitral valvulotomy after the age of fifty	Skagen, K.; Hansen, J. F.; Olesen, K. H.	1978	Scandinavian Journal of Thoracic and Cardiovascular Surgery	12(2)		85-89	Not MAC thrombus
794	Repeated prosthesis of the mitral valve with correction of tricuspid insufficiency and removal of giant calcified thrombus from the left atrium 18 years after mitral valve prosthesis. [Russian]	Skopin, I. I.; Faminskii, D. O.; Sudarikov, V. F.; Khasan, A.; Filippov, A. A.	1991	Grudnaia i serdechno-sosudistaia khirurgiia / Ministerstvo zdravookhraneniia SSSR [i] Vsesoiuznoe nauchnoe obshchestvo khirurgov		12	58-59	Prosthetic valve
795	Valvotomy in calcific mitral stenosis	Smith, G. H.; Belcher, J. R.	1970	Circulation. Conference: American Heart Association Scientific Sessions, AHA	140	Supplement 1		Valve intervention
796	Open mitral valvotomy. Effect of preoperative factors on result	Smith, W. M.; Neutze, J. M.; Barratt-Boyes, B. G.; Lowe, J. B.	1981	Journal of Cardiology Cases	21	1	07-May	Valve intervention

797	"High Risk" features of mitral annular calcification on echocardiography are associated with cerebral infarction	Smith, A. A.; Steffens, J. D.; Baxa, A. J.; Reyes-Castro, J.; Rodin, H.; Simegn, M. A.; Asinger, R. W.	2019	British Heart Journal	32	2	198-202	Not MAC thrombus
798	Calcific embolus resulting in ST elevation myocardial infarction: A rare complication of mitral annular calcification	Smith, A. A. H.; Wananu, M. K.; Bachour, F.	2020	Journal of Thoracic & Cardiovascular Surgery	82	5	738-51	Not MAC thrombus
799	Sodium-Thiosulfate Induced Life-Threatening Metabolic Acidosis Limiting Treatment of Calciphylaxis	Sohal, R.; George, T.	2020	The American Journal of Case Reports	21		e919926	Prosthetic valve
800	Echocardiographic detection of left atrial mobile calcium debris of trido valve surgery -A case report	Song, Y.; Shim, J. K.; Sun, J. M.; Lee, B.; Kwak, Y. L.	2014	Korean Journal of Anesthesiology	66(4)		314-316	Not MAC thrombus
801	Echocardiographic assessment of heart valve prostheses	Sordelli, C.; Severino, S.; Ascione, L.; Coppolino, P.; Caso, P.	2014	Journal of Cardiovascular Echography	24(4)		103-113	Prosthetic valve
802	[Pathology of calcareous emboli during calcified valvular diseases of the left side of the heart]	Soulie, P.; Caramanian, M.; Soulie, J.	1969	Semaine des Hopitaux	45	40	2445-54	Not MAC thrombus
803	Huge pseudoaneurysm after mitral valve replacement	Sousa, C.; Pinho, T.; Almeida, P.; Martins, E.; Pinho, P.; Maciel, M. J.	2014	Heart Lung and Circulation	23(3)		e105-e106	Valve intervention
804	Association between mitral annular calcification and carotid atheroma	Soylu, M.; Demir, A. D.; Arda, K.; Uzun, Y.; Goksel, S.	2001	Angiology	52	3	201-4	Not MAC thrombus
805	Caseous calcification of the mitral annulus: not always a benign condition!	Spapen, J.; Scott, B.	2017	The International Journal of	33	5	683-685	Not MAC thrombus

				Cardiovascular Imaging				
806	Experiences with the Carpentier techniques of mitral valve reconstruction in 103 patients (1980-1985)	Spencer, F. C.; Colvin, S. B.; Culliford, A. T.; Isom, O. W.	1985	Journal of Thoracic & Cardiovascular Surgery	90	3	341-50	Not MAC thrombus
807	Calcified Amorphous Tumour of the Mitral Annulus Imaged With Transoesophageal Three-Dimensional Echocardiography: A Rare Cause of Systemic Embolism	Squeri, A.; Gaudenzi, E.; Pardeo, A.; Tripodi, A.	2021	Heart Lung and Circulation	30(2)		e50-e51	Calcified amorphous tumor, not MAC thrombus
808	Calcified tumefaction in the left atrium associated with aortic and mitral valve insufficiency. [Serbian]	Stanic, R.; Cvetkov, R.; Blazic, M.; Popi, J.	1981	Medicinski pregled	34(9-10)		459-464	Not MAC thrombus
809	Calcific coronary embolization associated with cardiac valve replacement. Necropsy X ray study	Steiner, I.; Hlava, A.; Prochazka, J.	1976	Ceskoslovenska Patologie	21	2	87-95	Valve intervention
810	Spontaneous calcific coronary embolisation: necropsy x-ray study	Steiner, I.; Hlava, A.	1980	Journal of Clinical Pathology	33	10	984-9	Not MAC thrombus
811	[Calcification in the heart. I. Spontaneous coronary embolism due to calcified material]	Steiner, I.	1985	British Heart Journal	38(8)		816-820	Not MAC thrombus
812	Aortic valve allografts for mitral valve replacement	Stinson, E. B.; Griepp, R. B.; Bieber, C. P.; Shumway, N. E.	1975	Surgery	77	6	861-7	Valve intervention
813	[Early and late results following surgical management of mitral valve stenosis (report on 700 operated patients)]	Storch, H. H.; Trede, M.; Schmitz, W.	1968	Bruns Beitrage fur Klinischen Chirurgie	216	7	587-96	Valve intervention
814	Toothpaste tumor - To be or not to be operated?	Strambu, L.; Lazar, A.; Mihalcea, I.; Strambu, V.	2017	Clujul Medical	90(Supplement 4)		S21	Not MAC thrombus

815	Chameleonic appearance of caseous calcification of the mitral valve - still a problem for its appropriate management	Streian, C. G.; Lascu, A.; Sosdean, R.; Dima, C. N.; Grosu, F.; Costache, A.; Motoc, A. G. M.	2020	Romanian Journal of Morphology & Embryology	61	2	545-550	Not MAC thrombus
816	Calcification of the branches of the external carotid artery detected by panoramic radiography: a case report	Suarez-Cunqueiro, M. M.; Duker, J.; Liebehenschel, N.; Schon, R.; Schmelzeisen, R.	2002	Oral Surgery Oral Medicine Oral Pathology Oral Radiology & Endodontics	94	5	636-40	Not MAC thrombus
817	Multimodality imaging for assessment of coronary embolus	Sultan, A.; Goela, A.; Tweedie, E.; Awan, K.; Lavi, S.	2015	Canadian Journal of Cardiology	31	3	364.e5-7	Not MAC thrombus
818	Three mechanisms of early failure of transcatheter aortic valves: Valve thrombosis, cusp rupture, and accelerated calcification	Summers, M. R.; Cremer, P. C.; Jaber, W. A.	2017	Journal of Thoracic and Cardiovascular Surgery	153(5)		e87-e93	Prosthetic valve
819	Multiple coronary emboli from a calcified rheumatic aortic valve	Suri, R.; Jeresaty, R. M.	1994	Circulation	90(1)		623	Rheumatic valve
820	A rapidly growing cardiac calcified amorphous tumour diagnosed after coronary artery bypass graft surgery: a case report	Suzue, T.; Sawayama, Y.; Suzuki, T.; Nakagawa, Y.	2021	European Heart Journal. Case Reports	5	8	ytab243	No thrombus on MAC
821	The Ball valve experience over three decades	Swanson, J. S.; Starr, A.	1989	Annals of Thoracic Surgery	48(4 SUPPL.)		S51-S52	Prosthetic valve
822	Guidelines	Szczerbo-Trojanowska, M.	2019	CardioVascular and Interventional Radiology	42(3 Supplement)		S144-S145	Not MAC thrombus
823	[Late occlusive thrombosis of mitral prosthesis with sinus rhythm. Report of two surgically treated patients]	Tabone, X.; Berdah, J.; Le Feuvre, C.; Baubion, N.; Heulin, A.; Walter, A.; Vacheron, A.	1990	Annales de Cardiologie et d Angeiologie	39	8	467-70	Prosthetic valve

824	Five to 15 year study of mitral commissurotomy (Japanese). [Japanese]	Taira, A.; Nishimura, M.; Amako, H.	1976	Journal of the Japanese Association for Thoracic Surgery	24(9)		1127-1133	Valve intervention
825	A case of bioprosthetic mitral valvular dysfunction due to pannus-formation. [Japanese]	Takagi, H.; Terada, Y.; Shimoyama, Y.; Ino, T.; Wanibuchi, Y.; Furuta, S.	1990	Kyobu geka	The Japanese journal of thoracic surgery. 43(7)		569-572	Prosthetic valve
826	The Role of Cardiac Computed Tomography in Valve Disease and Valve Intervention Planning	Takigami, A. K.; Ghoshhajra, B.; Hedgire, S.	2021	Current Treatment Options in Cardiovascular Medicine	23(1) (no pagination)			Not MAC thrombus
827	Autologous right atrial wall patch for closure of atrial septal defects	Talwar, S.; Choudhary, S. K.; Mathur, A.; Kumar, A. S.	2007	Annals of Thoracic Surgery	84	3	913-6	Not MAC thrombus
828	[Percutaneous valvuloplasty in mitral stenosis]	Tamburino, C.; Russo, G.; Di Paola, R.; Drago, A.; Aiello, R.; Greco, G.; Felis, S.; Castania, G.; Deste, W.; Calvi, V.; et al.,	1993	Cardiologia	38	1	17-Jul	Not MAC thrombus
829	Over 10 years clinical outcomes in patients with mitral stenosis with unilateral commissural calcification treated with catheter balloon commissurotomy: single-center experience	Tanaka, S.; Watanabe, S.; Matsuo, H.; Segawa, T.; Iwama, M.; Hirose, T.; Takahashi, H.; Ono, K.; Warita, S.; Kojima, T.; Minatoguchi, S.; Fujiwara, H.	2008	Journal of Cardiology	51	1	33-41	Not MAC thrombus
830	Pulmonary hypertension in hospitalized end-stage renal disease patients	Taweesedt, P.; Chukiat, C.; Edwards, P.;	2016	Nephrology	21(Supplement 2)		38-39	Not MAC thrombus

		Disthabanchong, S.						
831	Use of tornus for a lesion that is easily balloon crossable but no expandible despite high pressure	Temiz, A.; Bostan, M.	2014	Erciyes Tip Dergisi	36(1)		43-46	Not MAC thrombus
832	10-year results of On-X bileaflet mechanical heart valve in the aortic position: low target INR regimen in Japanese	Teshima, H.; Ikebuchi, M.; Miyamoto, Y.; Tai, R.; Sano, T.; Kinugasa, Y.; Irie, H.	2017	General Thoracic and Cardiovascular Surgery	65(8)		435-440	Not MAC thrombus
833	A Review of JACC Journal Articles on the Topic of Cardiac Imaging: 2011-2012	The, Editors	2013	Journal of the American College of Cardiology	62(14)		e29-e141	Not MAC thrombus
834	Computed Tomography for Structural Heart Disease and Interventions	Theriault-Lauzier, P.; Spaziano, M.; Vaquerizo, B.; Buithieu, J.; Martucci, G.; Piazza, N.	2015	Interventional Cardiology Review	10(3)		149-154	Not MAC thrombus
835	Pathological substrates of thrombus formation after heart valve replacement with the Hancock bioprosthesis	Thiene, G.; Bortolotti, U.; Panizzon, G.; Milano, A.; Gallucci, V.	1980	Journal of Thoracic and Cardiovascular Surgery	80(3)		414-423	Prosthetic vale
836	Degenerative, non-atherosclerotic cardiovascular disease in the elderly: a clinico-pathological survey	Thiene, G.; Valente, M.	1990	Aging-Clinical & Experimental Research	2	3	231-44	Not MAC thrombus
837	Evaluation of embolic cardiac disease after cerebral ischemic events. [French]	Thomas, D.; Lascault, G.; Drobinski, G.; Lechat, P.; Isnard, R.; Evans, J.; Grosgogeat, Y.	1991	Archives des Maladies du Coeur et des Vaisseaux	84(11 SUPPL.)		1689-1697	Not MAC thrombus
838	A case of right middle cerebral artery atendonectomy' following mitral valve replacement surgery	Thomas, M. C.; Almandoz, J. E. D.; Todd, A. J.; Young, M. L.;	2017	BMJ Case Reports	2017 (no pagination)			Prosthetic valve

		Fease, J. L.; Scholz, J. M.; Milner, A. M.; Mulder, M.; Kayan, Y.						
839	Heme oxygenase derived carbon monoxide and iron mediated plasmatic hypercoagulability in a patient with calcific mitral valve disease	Thompson, J. L., 3rd; Nielsen, V. G.; Castro, A. R.; Chen, A.	2015	Journal of Thrombosis & Thrombolysis	39	4	532-5	Not MAC thrombus
840	Medtronic mosaic porcine bioprosthesis satisfactory early clinical performance	Thomson, D. J.; Jamieson, W. R.; Dumesnil, J. G.; Busse, E. F.; Peniston, C. M.; Metras, J.; Abel, J. G.; Sullivan, J. A.; Parrott, J. C.	1998	Annals of Thoracic Surgery	66	6 Suppl	S122-5	Prosthetic valve
841	Fibroelastoma as a very rare primal heart tumor	Tomaszewski, A.; Topyla, W.; Wojtkowska, A.; Tomaszewski, M.; Stettner-Leonkiewicz, D.; Wysokinski, A.; Czekajska-Chehab, E.	2017	European Heart Journal Cardiovascular Imaging	18(Supplement 3)		iii168	Not MAC thrombus
842	Development of non-bacterial thrombotic endocarditis after percutaneous transvenous mitral commissurotomy for severely calcified mitral stenosis	Tomimoto, S.; Ito, S.; Suzuki, T.; Mishima, A.; Suzumura, H.; Takeda, Y.; Yamada, Y.; Horio, T.; Goto, A.; Suzuki, S.; Fukutomi, T.; Itoh, M.	2000	Japanese Heart Journal	41	3	411-6	Not MAC thrombus
843	Evaluation of valvular heart diseases with computed tomography	Tomoda, H.; Hoshiai, M.; Matsuyama, S.	1982	Japanese Circulation Journal	46	4	402-6	Not MAC thrombus

844	Evaluation of transesophageal echocardiography in detecting cardiac sources of emboli in ischemic stroke patients	Toodeji, M. A.; Izadi, S.; Shariat, A.; Nikoo, M. H.	2015	Medical Journal of the Islamic Republic of Iran	29		237	Not MAC thrombus
845	Valvular heart disease in women. [Spanish]	Tornos, P.	2006	Revista Espanola de Cardiologia	59(8)		832-836	Not MAC thrombus
846	Cardiac sources of embolism in cerebral ischemia. [Greek]	Toumanidis, S. T.	1995	Hellenic Journal of Cardiology	36(5)		479-487	Not MAC thrombus
847	[Successful postoperative percutaneous cardiopulmonary support(PCPS) for the right-heart failure in a patient with recurrent mitral valve stenosis and a calcified left atrium; report of a case]	Toyama, S.; Fukasawa, M.; Kawahara, Y.; Suzuki, K.; Takahara, S.; Tanaka, S.	2013	Kyobu Geka - Japanese Journal of Thoracic Surgery	66	12	1092-5	Not MAC thrombus
848	From Mitral Annular Calcification to Calcified Amorphous Tumor	Toyokawa, N.; Okura, H.; Saito, Y.	2018	Internal Medicine	57	3	443	Calcified amorphous tumor, not MAC thrombus
849	Outcomes of Mitral Valve Replacement Over 20 Years	Tsang, C. F.; Fong, L.; Ho, E.; Akhunji, Z.; Grant, P.; Wolfenden, H.	2021	Heart Lung and Circulation	30(Supplement 1)		S5	Valve intervention
850	Implantation of three transcatheter aortic valves for embolization of two valves caused by under-expansion: a case report	Tsuda, M.; Shutta, R.; Nishino, M.; Tanouchi, J.	2021	European Heart Journal - Case Reports	5(1) (no pagination)			Not MAC thrombus
851	Accessory mitral valve in an elderly man on maintenance hemodialysis	Tsujimoto, K.; Mizuno, R.; Yoshida, H.; Morishima, R.; Shinki, Y.; Okamoto, Y.; Fujimoto, S.	2009	Journal of Echocardiography	7	4	80	Not MAC thrombus
852	Choice of surgical technique and results of surgical operations in	Tsukerman, G. I.; Kosach, G. A.;	1974	Vestn.Akad.Med.Nauk.Sssr	29	6	63-67	Not MAC thrombus

	defects of the mitral valve (Russian). [Russian]	Semenovsky, M. L.						
853	Experience gained with acquired defects of the aortic valve (Russian). [Russian]	Tsukerman, G. I.; Semenovsky, M. L.; Mikhina, V. S.	1975	Grudnaya Khirurgiya	17(1)		16-Sep	Not MAC thrombus
854	Giant left atrial thrombus despite anticoagulation with apixaban in a patient with mitral stenosis and atrial fibrillation	Turek, L.; Sadowski, M.; Janion-Sadowska, A.; Kurzawski, J.; Andrychowski, J.	2021	American Journal of Case Reports	22(1) (no pagination)			Not MAC thrombus
855	Surgery of the mitral valve	Turner, R. W.	1967	British Heart Journal	29	5	641-5	Valve intervention
856	Immediate and long-term outcome of percutaneous mitral valvotomy in patients 65 years and older	Tuzcu, E. M.; Block, P. C.; Griffin, B. P.; Newell, J. B.; Palacios, I. F.	1992	Circulation	85	3	963-71	Valve intervention
857	Assessment of planimetric mitral valve area using 16-row multidetector computed tomography in patients with rheumatic mitral stenosis	Ucar, O.; Vural, M.; Cetin, Z.; Gokaslan, S.; Gursoy, T.; Pasaoglu, L.; Koparal, S.; Aydoglu, S.	2011	Journal of Heart Valve Disease	20(1)		13-17	Rheumatic valve
858	Should we excise extensive mitral annular calcification or not?	Ulular, O.; Oc, B.; Yildirim, S.; Oc, M.	2011	Heart Surgery Forum	1)		S70	Not MAC thrombus
859	Importance of Finding Embolic Sources for Patients with Embolic Stroke of Undetermined Source	Umemura, T.; Nishizawa, S.; Nakano, Y.; Saito, T.; Kitagawa, T.; Miyaoka, R.; Suzuki, K.; Yamamoto, J.	2019	Journal of Stroke and Cerebrovascular Diseases	28(7)		1810-1815	Not MAC thrombus
860	Ultrasonic decalcification of calcified valve and annulus during heart valve replacement	Unal, M.; Sanisoglu, I.; Konuralp, C.; Akay, H.; Orhan, G.; Aydogan, H.;	1996	Texas Heart Institute Journal	23	2	Jul-85	Not MAC thrombus

		Aka, S. A.; Eren, E. E.						
861	Cardiac surgery in patients with a porcelain aorta in the era of transcatheter valve implantation	Urbanski, P. P.; Raad, M.; Wagner, M.; Heinz, N.; Reents, W.; Diegeler, A.	2013	European Journal of Cardio-thoracic Surgery	44(1)		48-53	Not MAC thrombus
862	Results of transcatheter mitral valve implantation in patients with bioprosthesis or annuloplasty failure or mitral annulus calcification	Urena, M.; Himbert, D.; Brochet, E.; Lecomte, M.; Carrasco, J. L.; Ghodbane, W.; Alkhoder, S.; Raffoul, R.; Cimadevilla, C.; Abtan, J.; Messika-Zeitoun, D.; Iung, B.; Nataf, P.; Vahanian, A.	2017	European Heart Journal	39	28	2679-2689	Not MAC thrombus
863	Clinical and haemodynamic outcomes of balloon-expandable transcatheter mitral valve implantation: a 7-year experience	Urena, M.; Brochet, E.; Lecomte, M.; Kerneis, C.; Carrasco, J. L.; Ghodbane, W.; Abtan, J.; Alkhoder, S.; Raffoul, R.; Iung, B.; Nataf, P.; Vahanian, A.; Himbert, D.	2018	European Heart Journal	38(Supplement 1)		847	Valve intervention
864	Cardiac valvular disease and stroke	Usher, B. W.	1993	Neurologic Clinics	11	2	391-8	Not MAC thrombus
865	Percutaneous mitral valvuloplasty. [French]	Vahanian, A.; Michel, P. L.; Cormier, B.; Acar, J.	1990	Polski Przeglad Kardiologiczny	5(3)		257-261	Valve intervention
866	Immediate and mid-term results of percutaneous mitral commissurotomy	Vahanian, A.; Michel, P. L.; Cormier, B.; Ghanem, G.;	1991	Revue du Praticien	50(15)		1679-1683	Valve intervention

		Vitoux, B.; Maroni, J. P.; Cazaux, P.; Acar, J.						
867	Valvular stenosis: Treatments by percutaneous dilatation. [French]	Vahanian, A.; Luxereau, P.; Brochet, E.	2000	Schweizerische Medizinische Wochenschrift	120(45)		1687-1693	Valve intervention
868	15 years experience with percutaneous mitral commissurotomy. [Polish]	Vahanian, A.	2003	European Heart Journal	12 Suppl B		Sep-84	Valve intervention
869	Cardiac Causes of Stroke	Vahedi, K.; Amarenco, P.	2000	Current Treatment Options in Neurology	2	4	305-318	Not MAC thrombus
870	Experimental Evaluation of a Novel Percutaneous Transseptal Catheter-Based Mitral Valve Replacement Technology	Vahl, T. P.; Grogan, A.; Cheng, Y.; Yi, G.; Von Oepen, R.; Khalique, O. K.; Wallace, D. T.; Modine, T.; Granada, J. F.	2020	Circulation: Cardiovascular Quality and Outcomes.				Not MAC thrombus
871	Concurrent Rheumatic Mitral Stenosis with Sickling Hemoglobinopathy	Vaideeswar, P.; Singh, H.; Singaravel, S.	2017	IHJ Cardiovascular Case Reports (CVCR)	1(3)		119-121	Rheumatic valve
872	Pathology of the Pericarbon bovine pericardial xenograft implanted in humans	Valente, M.; Ius, P.; Bortolotti, U.; Talenti, E.; Bottio, T.; Thiene, G.	1998	Journal of Heart Valve Disease	7	2	180-9	Prosthetic valve
873	Emergency valve-preserving operations in mitral stenosis under conditions of craniocerebral hypothermia. [Russian]	Valyka, E. N.; Shchukin, V. S.; Naumenko, S. E.	1988	Grudnaya Khirurgiya	30(5)		14-Oct	Not MAC thrombus
874	Detection of Major Cardioembolic Sources in Real-World Patients with Ischemic Stroke or Transient Ischemic Attack of Undetermined Cause	Van Der Maten, G.; Reimer, J. M. B.; Meijs, M. F. L.; Von Birgelen, C.; Brusse-Keizer, M.	2021	Cerebrovascular Diseases Extra	11(1)		22-28	Not MAC thrombus

		G. J.; Den Hertog, H. M.						
875	[Silent Calcified Mitral Stenosis. Pulmonary Infarct]	Varelade, Seijas; Lahoz,	1965	Revista Clinica Espanola	96		124	Not MAC thrombus
876	Native aortic valve endocarditis complicated by splenic infarction and giant mitral-aortic intervalvular fibrosa pseudoaneurysm- a case report and brief review of the literature	Varga, A.; Tilea, I.; Tatar, C. M.; Iancu, D. G.; Jiga, M. A.; Dumbrava, R. A.; Pop, M.; Suciu, H.	2021	Diagnostics	11(2) (no pagination)			Not MAC thrombus
877	Left atrial calcification detected by two-dimensional echocardiography	Vargas-Barron, J.; Sanchez-Ugarte, T.; Keirns, C.; Santana-Gonzalez, A.; Vazquez-Sanchez, J.	1988	Journal of Cardiovascular Ultrasonography	7(2)		161-163	RHD
878	The role of echocardiography in patients after ischemic stroke	Vavrova, J.	2018	European Journal of Neurology	25(Supplement 1)		1	Not MAC thrombus
879	[Calcified mitral stenosis: comparative study of long term results with valvular substitution and mitral commissurotomy]	Vazquez Rodriguez, C.; Quijano Pitman, F.; Sanchez Gonzalez, A.; Rebollar y Pliego, L.	1969	Archivos del Instituto de Cardiologia de Mexico	39	5	643-56	Valve intervention
880	Spontaneous left atrial dissection caused by mitral valve endocarditis	Vefali, H.; Durkin, M.; Singh, A.; Agrawal, S.; Longo, S.; Averbach, M.; Shirani, J.	2016	Cardiology (Switzerland)	134(Supplement 1)		417	Not MAC thrombus
881	Open mitral commissurotomy	Vega, J. L.; Fleitas, M.; Martinez, R.; Gallo, J. I.; Gutierrez, J. A.; Colman, T.; Duran, C. M.	1981	Annals of Thoracic Surgery	31	3	266-70	Not MAC thrombus

882	Clinical experience with porcine xenografts in the mitral position	Vejlsted, H.; Rasmussen, K.; Albrechtsen, O.	1984	Scandinavian Journal of Thoracic & Cardiovascular Surgery	18	1	Jun-33	Prosthetic valve
883	Closed-heart mitral valve surgery. [Italian]	Venere, G.; Barberis, L.; Lijoi, A.	1977	Minerva medica	68(16)		1087-1090	Not MAC thrombus
884	3D assessment of mitral annular dynamics in mitral stenosis: Acute alterations after percutaneous transvenous mitral commissurotomy	Venkateshvaran, A.; Sola, S.; Dash, P. K.; Annappa, C.; Manouras, A.; Winter, R.; Brodin, L. A.; Govind, S. C.	2014	European Heart Journal Cardiovascular Imaging	2)		ii206	Not MAC thrombus
885	Reconstruction of atrioventricular valves with photo-oxidized bovine pericardium	Verbrugghe, P.; Meuris, B.; Flameng, W.; Herijgers, P.	2009	Interactive Cardiovascular & Thoracic Surgery	9	5	775-9	Not MAC thrombus
886	The occurrence and management of left atrial thrombi in mitral valve surgery	Verska, J. J.; Ludington, L. G.; Walker, W. J.; Mundall, S.; Brewer, IIIrd L. A.	1974	Journal of Cardiovascular Surgery	15(5)		516-521	Not MAC thrombus
887	Long-term (1 to 4 years) results with heart valve prostheses (102 consecutive cases)	Vidne, B.; Salomon, J.; Eshkol, D.; Levy, M. J.	1969	Israel Journal of Medical Sciences	5	4	896-903	Prosthetic valve
888	Multimodality imaging of exuberant mitral annular calcification in a patient presenting with transient ischaemic attack	Vijayan, S.; Fielding, P.; Kahan, B.; Ionescu, A.	2011	Journal of the Royal College of Physicians of Edinburgh	41	4	316-8	No thrombus on MAC
889	Clinical and echocardiographic features of two large left atrial parietal thrombi	Vincelj, J.; Biocic, S.; Bergovec, M.; Biocina, B.; Husedzinovic, I.; Korusic, A.	1999	Acta Medica Croatica	53	1	Jul-45	Not MAC thrombus

890	Acute myocardial infarction due to left main embolization of calcified tissue from mitral valve subapparatus	Vitela Rodriguez, J. A.; Jimenez Diaz, V. A.; Chantada de la Fuente, D.; Hernandez Hernandez, E.; Fernandez Barbeira, S.; De Miguel Castro, A. A.; Baz Alonso, J. A.; Iniguez Romo, A.	2017	Journal of Cardiology Cases	15(6)		194-196	Calcified amorphous tumor, not MAC thrombus
891	Antithrombotic therapy in native heart valve disease	Voller, H.	2004	Journal of Heart Valve Disease	13	3	325-8	Not MAC thrombus
892	Mitral anular calcification: An age-stratified case-control study	Voyles, W. F.; Smalling, R.; Teague, S. M.; Anderson, J.; Johnsen, G.; Thadani, U.	1990	American Journal of Noninvasive Cardiology	4(6)		352-357	Not MAC thrombus
893	Early experience with the ionescu-shiley pericardial xenograft valve. Accelerated calcification in children	Walker, W. E.; Duncan, J. M.; Frazier, O. H., Jr.; Livesay, J. J.; Ott, D. A.; Reul, G. J.; Cooley, D. A.	1983	Ajnr: American Journal of Neuroradiology	35	8	1515-9	Prosthetic valve
894	Calcified cerebral emboli, a "do not miss" imaging diagnosis: 22 new cases and review of the literature	Walker, B. S.; Shah, L. M.; Osborn, A. G.	2014	Journal of Thoracic & Cardiovascular Surgery	86	4	570-5	Not MAC thrombus
895	Cardiovascular disease in the very elderly. Analysis of 40 necropsy patients aged 90 years or over	Waller, B. F.; Roberts, W. C.	1983	American Journal of Cardiology	51(3)		403-421	Not MAC thrombus
896	Patterns of failure in Hancock pericardial bioprostheses	Walley, V. M.; Rubens, F. D.; Campagna, M.; Pipe, A. L.; Keon, W. J.	1991	Journal of Thoracic & Cardiovascular Surgery	102	2	187-94	Prosthetic valve

897	Midterm outcomes of rheumatic mitral repair versus replacement	Wang, Y. C.; Tsai, F. C.; Chu, J. J.; Lin, P. J.	2008	Journal of the American College of Cardiology	69(16 Supplement 1)		S158-S159	Rheumatic valve
898	Left main bifurcation lesion with culotte stenting	Wang, W. T.; Wu, T. C.	2017	Annals of Thoracic Surgery	112(6)		2111-2112	Not MAC thrombus
899	Transcatheter Mitral Valve Replacement in Mitral Annular Calcification	Wang, Y.; Hu, X.	2021	International Heart Journal	49(5)		565-576	Not MAC thrombus
900	Transesophageal echocardiographic monitoring during vascular surgery in a patient with unanticipated critical aortic stenosis	Wasson, N.; Matyal, R.	2014	Journal of Cardiothoracic and Vascular Anesthesia	28(5)		1426-1428	Valve intervention
901	Not All Immobile Bioprosthetic Valve Cusps Are Thrombosed	Waterbury, T. M.; Raphael, C. E.; Padang, R.; Eleid, M. F.; Holmes, D. R.; Rihal, C. S.; Pislaru, S. V.	2017	JACC: Cardiovascular Interventions	10(12)		e117-e118	Prosthetic valve
902	Calcified atrial thrombus: complication of central venous hyperalimentation	Watts, M. A.; Innes, B. J.	1984	Texas Heart Institute Journal	11	1	Sep-76	Not MAC thrombus
903	First redo heart valve replacement: a 10-year analysis	Weerasinghe, A.; Edwards, M. B.; Taylor, K. M.	1999	Circulation	99	5	655-8	Valve intervention
904	Surgical management of caseous calcification of the mitral annulus	Wehman, B.; Dawood, M.; Ghoreishi, M.; Cheema, F.; Jones, J. W.; Kane, M. A.; Ward, C. W.; Gammie, J. S.	2015	Annals of Thoracic Surgery	99(6)		2231-2233	Not MAC thrombus
905	Commentary: An ounce of prevention	Wei, L. M.; Badhwar, V.	2021	JTCVS Techniques	6		59-60	Not MAC thrombus

906	Stroke prevention: cardiac and carotid-related stroke	Wein, T. H.; Bornstein, N. M.	2000	Neurologic Clinics	18	2	321-41	Not MAC thrombus
907	Coronary sequelae of mitral stenosis	Wiedemann, S.; Stolte, D.; Simonis, G.	2009	European Heart Journal	30	22	2816	Not MAC thrombus
908	Coronary arterial embolism due to valvular debris after percutaneous valvuloplasty of calcific mitral stenosis	Wiegand, V.; Tebbe, U.; Helmchen, U.; Kreuzer, H.	1988	Clinical Cardiology	11	11	793-6	Valve intervention
909	A huge left atrial thrombus in patient with severe mitral stenosis	Wierzbowska-Drabik, K.; Marszal-Marciniak, M.; Mozdzan, M.; Kurpesa, M.	2011	Polski Przeglad Kardiologiczny	13(4)		277-279	Rheumatic valve
910	Mobile components associated with rapidly developing mitral annulus calcification in patients with chronic renal failure: review of mobile elements associated with mitral annulus calcification	Willens, H. J.; Ferreira, A. C.; Gallagher, A. J.; Morytko, J. A.	2003	Echocardiography	20	4	363-7	No thrombus on MAC
911	Tissue valves in young patients--a recipe for disaster	Williams, M. A.	1991	Journal of Cardiac Surgery	6	4 Suppl	620-3	Prosthetic valve
912	Cardiovascular disease in patients with retinal arterial occlusion	Wilson, L. A.; Warlow, C. P.; Russell, R. W.	1979	Lancet	1	8111	292-4	Not MAC thrombus
913	Embolism frequency in cases of mitral stenosis. [German]	Wink, K.; Schweiger, M.; Reindell, H.	1975	Medizinische Klinik	70(42)		1675-1681	Not MAC thrombus
914	Calcific retinal emboli and collateral shunting in a woman with rheumatic heart disease	Winterkorn, J. M.	1995	Archives of Ophthalmology	113	11	1464-5	Rheumatic disease
915	Tumors of the cardiac valves: imaging findings in magnetic resonance imaging, electron beam computed tomography, and echocardiography	Wintersperger, B. J.; Becker, C. R.; Gulbins, H.; Knez, A.; Bruening, R.;	2000	European Radiology	10	3	443-9	Not MAC thrombus

		Heuck, A.; Reiser, M. F.						
916	Neonatal Enterovirus Myocarditis With Severe Dystrophic Calcification: Novel Treatment With Pocapavir	Wittekind, S. G.; Allen, C. C.; Jefferies, J. L.; Rattan, M. S.; Madueme, P. C.; Taylor, B. N.; Moore, R. A.	2017	Journal of Investigative Medicine High Impact Case Reports	5	3		Not MAC thrombus
917	Congenital disorders of glycosylation and intellectual disability	Wolfe, L. A.; Krasnewich, D.	2013	Developmental Disabilities Research Reviews	17(3)		211-225	Not MAC thrombus
918	New device angioplasty: The impact on restenosis	Wong, C. S.; Leon, M. B.; Popma, J. J.	1993	Coronary Artery Disease	4(3)		243-253	Not MAC thrombus
919	A challenging case of paravalvular leakage closure	Wong, C. Y.	2016	European Heart Journal Cardiovascular Imaging	17(Supplement 2)		ii214	Not MAC thrombus
920	Thrombotic calcific mitral stenosis. Morphology of the calcific mitral valve	Wooley, C. F.; Baba, N.; Kilman, J. W.; Ryan, J. M.	1974	Circulation	49	6	1167-74	Not MAC
921	The Role of Echocardiography During Mitral Valve Percutaneous Interventions	Wunderlich, N. C.; Beigel, R.; Siegel, R. J.	2013	Cardiology Clinics	31(2)		237-270	Not MAC thrombus
922	Left ventricular diverticulum mimicking cardiac tumor	Wybraniec, M. T.; Wrobel, W.; Myszor, J.; Mizia-Stec, K.	2017	Echocardiography	34	10	1548-1551	Not MAC thrombus
923	Ubiquitin-positive foam cells are identified in the aortic and mitral valves with atherosclerotic involvement	Yamada, T.; Satoh, S.; Sueyoshi, S.; Mitsumata, M.; Matsumoto, T.; Ueno, T.; Uehara, K.; Mizutani, T.	2009	Journal of Atherosclerosis & Thrombosis	16	4	472-9	Not MAC thrombus

924	A case of chronic multiple pulmonary thromboembolism associated with mitral stenosis. [Japanese]	Yamakado, T.; Osata, S.; Hayashi, T.	1981	Medical Journal of Kinki University	6(2)		215-221	Rheumatic valve
925	Cardiac calcified amorphous tumor stuck in the aortic valve that mimicked a chameleon's tongue: report of a case	Yamamoto, M.; Nishimori, H.; Wariishi, S.; Fukutomi, T.; Kond, N.; Kihara, K.; Tashiro, M.; Tanioka, K.; Orihashi, K.	2014	Surgery Today	44	9	1751-3	Calcified amorphous tumor, not MAC thrombus
926	Cardiac calcified amorphous tumour associated with multiple myeloma	Yamanaka, T.; Fukatsu, T.; Uchimuro, T.; Takanashi, S.	2020	BMJ Case Reports	13(4) (no pagination)			Calcified amorphous tumor, not MAC thrombus
927	A waving horn on the big mitral annulus calcification: Caseous calcification of the mitral annulus with abscess formation	Yang, L. T.; Wu, H. Y.; Luo, C. Y.; Tsai, W. C.	2014	European Heart Journal	1)		1207	Not MAC thrombus
928	Mitral valve repair for isolated libman-sacks endocarditis in a patient with primary antiphospholipid syndrome	Ye, T.; Wang, J.; Liao, S.	2021	International Heart Journal	62(1)		181-185	Not MAC thrombus
929	Giant Caseous Calcification on Tricuspid Annulus Mimicking Cardiac Metastasis in a Patient with Colon Cancer	Yesin, M.; Toprak, C.; Kalcik, M.; Bayam, E.; nanir, M.; Ozkan, M.	2015	Echocardiography	32(12)		1885-1886	Not MAC thrombus
930	Severe calcific chronic constrictive tuberculous pericarditis	Yetkin, U.; Ilhan, G.; Calli, A. O.; Yesil, M.; Gurbuz, A.	2008	Texas Heart Institute Journal	35(2)		224-225	Not MAC thrombus
931	Multiple Late Complications After Takeuchi Repair of Anomalous Left Coronary Artery From the Pulmonary Artery	Yokohama, F.; Toh, N.; Kotani, Y.; Watanabe, N.; Takaya, Y.; Akagi, T.; Kasahara, S.; Ito, H.	2021	JACC: Case Reports	3(5)		731-735	Not MAC thrombus

932	Stenosis of the bicuspid aortic valve with systemic lupus erythematosus	Yoshikai, M.; Muraya, J.; Fujita, H.	2006	Japanese Journal of Thoracic and Cardiovascular Surgery	54(1)		16-18	Not MAC thrombus
933	Cardiac calcified amorphous tumors in a patient with hemodialysis for diabetic nephropathy	Yoshimura, S.; Kawano, H.; Minami, T.; Tsuneto, A.; Nakata, T.; Koga, S.; Ikeda, S.; Hayashi, T.; Maemura, K.	2017	Internal Medicine	56(22)		3057-3060	Calcified amorphous tumor, not MAC thrombus
934	On a case of refractory congestive heart failure	Yousef, N.	1986	Revista Medico-Chirurgicala a Societatii de Medici Si Naturalisti Din Iasi	90	2	385-6	Not MAC thrombus
935	Tumors and tumor-like lesions of the heart valves	Yuan, S. M.; Jing, H.; Lavee, J.	2009	Rare Tumors	1	2	e35	Not MAC thrombus
936	Idiopathic massive myocardial calcification: A rare cause of heart failure	Zagnoni, S.; Riva, L.; Pallotti, M. G.; Coutsoumbas, G. V.; Casella, G.; Pavesi, P. C.; Di Pasquale, G.	2014	Giornale Italiano di Cardiologia	2)		e34-e35	Not MAC thrombus
937	Mechanical thrombectomy for calcified middle cerebral artery embolus	Zeigler, G.; Elangovan, C.; Niazi, M.; El-Ghanem, M.; Kalapos, P.; Ibrahimi, M.	2019	Annals of Neurology	86(Supplement 24)		S49-S50	Not proven MAC thrombus
938	[Operative risks in commissurotomies for mitral stenosis]	Zenker, R.; Seidel, W.; Ruckert, U.	1965	Munchener Medizinische Wochenschrift	107	37	1741-7	Not MAC thrombus
939	Ischemic colitis as a complication of acute myocardial infarction	Zhang, R.; Sun, J. P.; Chong, J.; Liu,	2015	International Journal of Cardiology	185		50-51	Not MAC thrombus

		B.; Wang, F.; Yu, C. M.						
940	Early bioprosthesis failure: Report of three cases and literature review	Zhao, A.; Ben, Z.; Fan, Y.; Xiaohong, L.; Zhiyun, X.	2015	Heart Surgery Forum	18(2)		E59-E62	Prosthetic valve
941	Surgical procedure in complicated mitral stenosis. [Russian]	Zorin, A. B.; Roostar, L. A.; Novikov, V. K.; Bui, H. T.	1982	Vestnik khirurgii imeni I	I. Grekova. 128(1)		53-57	Not MAC thrombus
942	The porcine aortic valve bioprosthesis: a significant alternative	Zuhdi, N.	1976	Annals of Thoracic Surgery	21	6	573-5	Not MAC thrombus
943	Artificial chordae	Zussa, C.	1995	Journal of Heart Valve Disease	4 Suppl 2		S249-54; discussion S254	Not MAC thrombus
944	Mitral valve replacement in the first 5 years of life	Zweng, T. N.; Bluett, M. K.; Mosca, R.; Callow, L. B.; Bove, E. L.	1989	Annals of Thoracic Surgery	47	5	720-4	Not MAC thrombus
